# MXene‐Based Room‐Temperature NO_2_ Gas Sensors: A Meta‐Analysis

**DOI:** 10.1002/advs.77530

**Published:** 2026-09-10

**Authors:** Alexander Khort, Jerome Geyer‐Klingeberg, Andreas Rathgeber, Suelen Barg

**Affiliations:** ^1^ Department of Chemistry KTH Royal Institute of Technology Stockholm Sweden; ^2^ Institute of Materials Resource Management University of Augsburg Augsburg Germany

**Keywords:** 2D materials, gas sensors, meta‐analysis, meta‐regression, MXene, nitrogen dioxide

## Abstract

The rapidly developing field of MXene‐based room‐temperature NO_2_ gas sensors is characterized by significant heterogeneity in material synthesis, treatment methods, and sensor design. This variation complicates the identification of best technological practices and hinders direct comparison of reported performance metrics. This study addresses this challenge by performing the first comprehensive meta‐analysis of 1243 individual sensor measurements from 61 published, peer‐reviewed papers. The analysis establishes comparable key performance metrics for quantitative synthesis, including normalized sensing response, breakpoint, and sensitivity, and identifies a wide range of explanatory variables, with modifier type, intercalation, synthesis approach, surface state, and post‐synthesis treatment being the most influential factors, and a smaller contribution from morphology, while device‐level parameters such as electrode material are secondary. The resulting multidimensional analysis yields a quantitative assessment of structure–property–performance drivers and exposes systematic gaps in current sensor design. Beyond consolidating a fragmented literature, this work establishes a methodological blueprint for translating data heterogeneity into generalized design rules, accelerating the rational development of high‐performance MXene‐based chemical sensors.

## Graphical abstract

This study presents the first comprehensive meta‐analysis of MXene‐based NO_2_ sensors, decoding 32 study characteristics across 61 peer‐reviewed studies. By isolating materials chemistry as the primary performance driver over device‐level parameters, the authors establish a methodological blueprint and a predictive structure–function map to accelerate the rational development of high‐performance, room‐temperature NO_2_ sensors.

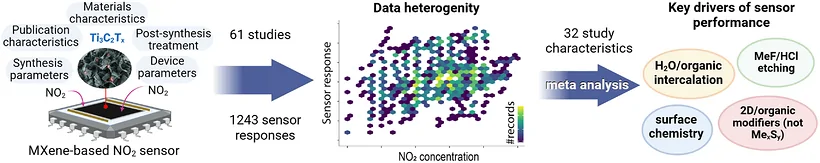

## 1 Introduction

Sensors, and gas sensors in particular, are essential components in the transition toward Industry 5.0 and the digital society of the future. The use of precise, low‐cost, highly efficient, and energy‐saving integrated sensors enables both global and local monitoring of industrial processes, early disease diagnosis, food quality control, air quality control, and environmental assessment. For instance, the effective monitoring and controlling of toxic gases, such as nitrogen dioxide (NO_2_), in the local atmosphere and industrial processes is a critical challenge. As a major atmospheric pollutant generated by industrial processes and vehicle emissions, NO_2_ poses significant health risks even at very low concentrations, with effects ranging from respiratory inflammation to adverse cardiovascular effects [1]. To mitigate these hazards, the WHO has established stringent air quality guidelines, recommending an annual mean concentration of no more than 10 µg/m^3^ (5.3 ppb) or an hourly mean of no more than 200 µg/m^3^ (∼106 ppb) [2]. This highlights the urgent need for advanced sensing materials and devices capable of reliably detecting and quantifying NO_2_ and other toxic gases in both ambient air and industrial processes. Furthermore, alignment with the key principles of future industry and society requires a focus on miniaturization, energy efficiency, enhanced sensitivity, selectivity, precision, long‐term operational stability without signal degradation, and environmental and biological safety throughout the entire life cycle of sensors and their components. These requirements, along with specific application‐driven demands, determine the most suitable sensor technologies and materials. Among the available alternatives, semiconductor‐based sensors have gained popularity in the sensing industry due to their simple structure, high sensing performance, compatibility with industrial processes, and ability to incorporate diverse sensing materials [3, 4, 5].

Within this context, two‐dimensional (2D) materials have emerged as a particularly attractive class of sensing nanomaterials [6, 7, 8]. Their key advantages include a high specific surface area available for gas adsorption, tunable surface chemistry for selective gas detection, favorable and adjustable electrical properties, compatibility with other sensing materials (e.g., oxides and metals), and relatively simple fabrication processes.

Among various functional 2D materials and structures, such as graphene (G), graphene oxide (GO), reduced graphene oxide (rGO), and transition metal dichalcogenides (TMDs), MXenes, a family of 2D transition metal carbides, nitrides, and carbonitrides, offer a unique combination of functional properties [9]. Their 2D layered structure, with outer transition metal layers and surfaces terminated by functional groups such as ─O, ─OH, ─F, and ─Cl, enables high surface reactivity and tunability. This rich surface chemistry, combined with a large surface area, facilitates strong gas adsorption and rapid, easily detectable charge transfer between the material and the adsorbed gas molecules. Importantly, unlike conventional semiconductor oxide gas sensors (e.g., WO_3_, ZnO, In_2_O_3_), MXene‐based sensors can be designed to operate at room temperature or even be self‐powered, making them highly energy‐efficient [7]. This makes MXenes promising candidates for the development of gas sensors for both oxidizing (e.g., NO_2_) and reducing (e.g., H_2_, NH_3_) gases, especially when high sensitivity at low (sub‐ppm to low ppm) gas concentrations is required, for instance, in the case of monitoring toxic gases.

The rapid growth of research on MXene‐based sensors has led to a vast and methodologically diverse body of literature, making it difficult to draw overarching conclusions about the optimal practices for sensor design, performance evaluation, and data reporting [10]. For instance, one study might report exceptional sensor response using a Ti_3_C_2_T*_x_* composite with a specific oxide, while another might achieve faster response and recovery times by modifying the MXene surface with a specific treatment [7, 11]. Further variations in synthesis protocols (e.g., etching methods), sensor fabrication (e.g., substrate and electrode materials), and testing conditions (e.g., humidity, temperature) contribute additional layers of complexity [12, 13]. This heterogeneity, which is a complex interplay of various design characteristics, makes it challenging to identify the critical factors that drive sensor performance.

To address this gap, this study presents the first comprehensive meta‐analysis on MXene‐based NO_2_ sensors and one of the first meta‐regression‐based heterogeneity analyses in materials science. Meta‐analysis is a statistical methodology designed specifically to synthesize diverse research results on the same topic in a systematic and quantitative manner [14, 15]. It allows a researcher to pursue two principal goals. The first is to estimate a genuine underlying effect by aggregating the results from all included primary studies, which helps to clarify the overall picture by averaging out the statistical noise inherent in individual experiments. The second objective is to investigate the sources of heterogeneity in the reported results. Instead of viewing the variation between studies as a limitation, meta‐analysis treats it as a source of information [16]. For instance, in the context of MXene‐based sensors, this allows a researcher to quantitatively assess the impact of key material characteristics (e.g., the presence of in situ oxides), synthesis protocols (e.g., the etching method), and sensor fabrication details (e.g., electrode materials) on the reported sensing performance. While the methods of meta‐analysis have roots in multiple fields, its modern application as a tool for systematic, evidence‐based reviews was largely pioneered within medical research [17], and has since become a cornerstone of research practice across a wide range of disciplines, such as psychology [18], economics [19], and business and management [20]. Its application within materials science is a more recent but growing trend [21, 22, 23, 24, 25].

Building on a systematic literature search that identified 61 articles providing 1243 individual measurements, the influence of a wide range of factors on sensor performance was analyzed. For this, 32 moderator variables were manually coded and grouped into categories reflecting material synthesis, sensor design, and experimental conditions. By applying state‐of‐the‐art meta‐analytic methods [26, 27, 28, 29], this research aims to (i) identify the aggregated sensor response across the primary literature, (ii) use heterogeneity analysis to determine which factors are most influential in optimizing sensor performance, and (iii) provide a methodological blueprint for conducting advanced meta‐analysis on the highly heterogeneous technical data characteristics of materials science.

## 2 Results and Discussion

### 2.1 Overall Sensor Response

**FIGURE 1:**
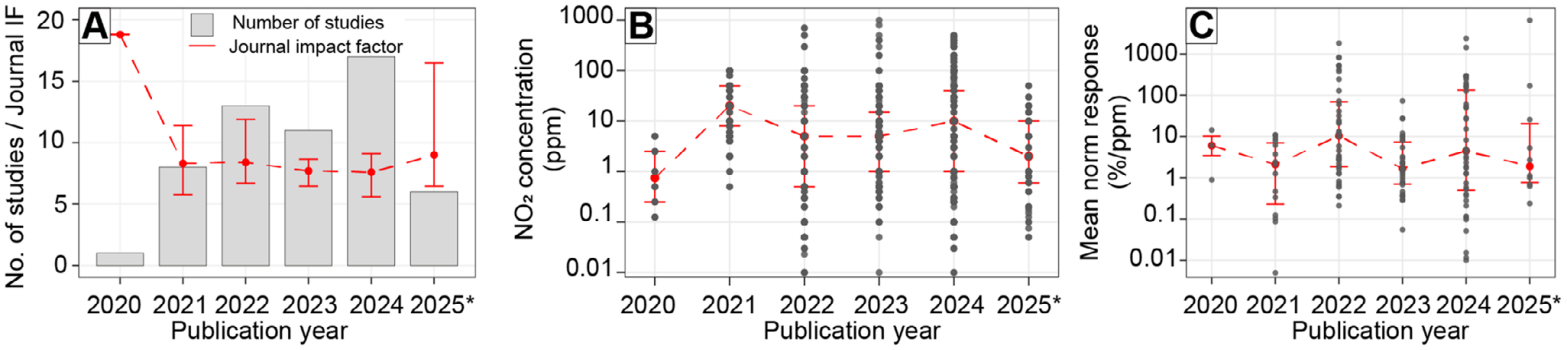
Publication‐year trends: (A) Number of studies per publication year, with the journal impact factor for that year overlaid (red: median with IQR). (B) NO_2_ concentration (ppm) on a logarithmic *y*‐axis versus publication year; gray points are individual measurements, red dots/whiskers show the per‐year median and IQR. (C) Mean normalized response per sample on a logarithmic *y*‐axis versus publication year; gray points are individual samples, red dots/whiskers show the per‐year median and IQR. *For 2025, the data are limited to papers published by October 3, 2025.

Figure 1 illustrates the dynamics of the research landscape by showing publication trends and median journal impact factor (Figure 1A), NO_2_ concentration studied in papers over time (Figure 1B), and mean normalized sensor response over time (Figure 1C). The number of studies published per year shows a strong upward trend since 2020, confirming the field's rapid expansion. This growth in the number of publications is correlated with a decrease and flattening in the median journal impact factor. The trend is consistent with the increasing maturity of the research field, resulting in a shift from publishing in broader, higher‐impact journals to more specialized, field‐specific journals. However, the per‐year medians of both the NO_2_ concentration (Figure 1B) and the mean normalized response (Figure 1C) have remained volatile and flat, rather than showing a clear trend in concentration range and sensor performance. This contrasts with the increasing number of publications, and confirms the focus on relatively high NO_2_ concentrations (>1 ppm). This focus is likely related to the persistent challenge of achieving consistently high sensor performance and establishing clear field development directions amidst escalating research effort, and highlights the necessity of a more in‐depth heterogeneity analysis.

**FIGURE 2:**
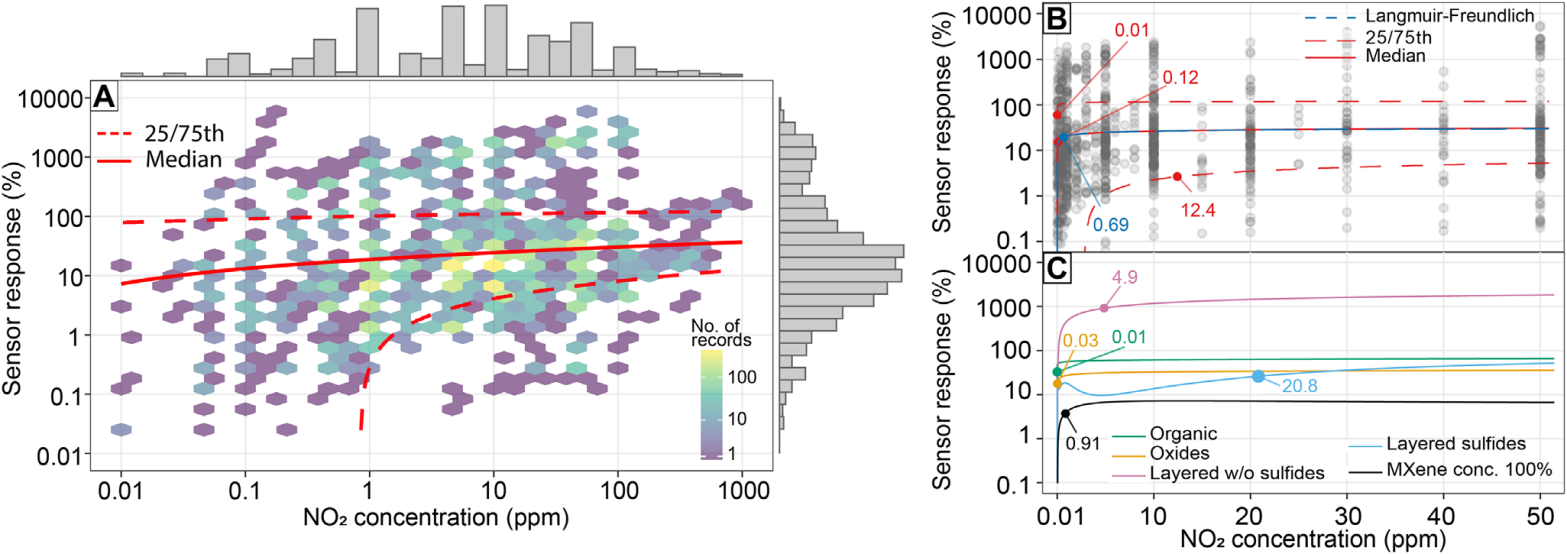
Distribution of MXene‐based NO_2_ sensor performance and meta‐analytic curves. (A) Sensor responses to NO_2_ (*r* = 1180 records, *n *= 56 studies) shown as a hex‐binned density plot on log–log axes over 0.01–1000 ppm; hexagon color indicates record count. The solid red curve is the conditional median, the dashed red curves are the 25th and 75th quantiles; marginal histograms show the univariate distributions. (B) Conditional median (solid red) with 25th/75th quantiles (dashed red) over 0.01–50 ppm (linear *x*‐axis, log *y*‐axis); gray points are individual observations and the blue dashed curve is the Langmuir–Freundlich (LF) fit. Red points mark the *C*_50_ (half‐maximum) of the median, the LF fit, and the 25th/75th quartile curves. (C) Meta‐analytical median curves for five MXene subgroups (pure MXene and oxide‐, organic‐, layered‐sulfide‐, and layered‐non‐sulfide‐modified materials) over 0.01–50 ppm (linear *x*‐axis, log *y*‐axis); dots mark the *C*_50_ points. All median curves were estimated using quantile GAMs in log NO_2_ with penalized cubic splines.

Figure 2A visualizes the distribution of MXene‐based NO_2_ sensor performance by a hexagonal binning plot of the 1180 sensor response measurements from the set of 56 primary studies (reduced from the full set of 61 studies after exclusion of outlier studies; see Section 4). Figure S1 shows an alternative visualization with the distribution of responses for different NO_2_ bins.

The red line highlights the smoothed conditional median trend, estimated using a quantile generalized additive model (qGAM), while marginal histograms show the univariate frequency distributions. The plot reveals a nonlinear, saturating MXene sensor response, with the response increasing with NO_2_ concentration before leveling off, as expected for solid‐state semiconductor sensors. At low concentrations, the median response is modest but increases around the 0.1–1 ppm range, reaching about 15%–20% at 1 ppm and rising further toward a maximum response of *R_max_* = 37%, with half‐maximum attained at *C_50_* = 0.94 ppm. The incremental response per concentration doubling changes little between 1 and 100 ppm: using the fitted median curve, the change in response between a given concentration and twice that concentration is 1.7 percentage points at 1 ppm and 1.9 percentage points at 100 ppm. The high‐concentration side does not fully plateau within the observed range; the median reaches 90% of its maximum near *x_90_* = 272 ppm.

Critically, Figure 2A underscores the substantial heterogeneity in reported MXene‐based NO_2_ sensor performance. The wide vertical spread of hex‐bins across the full concentration range, frequently spanning nearly an order of magnitude at any given NO_2_ level, reflects considerable inter‐study and inter‐sensor variability. For example, the interquartile (25%–75%) envelope around the median near *C*_50_ spans approximately 100 percentage points, corresponding to roughly one order of magnitude in response intensity. The marginal histograms illustrate that most measurements cluster within the 1–30 ppm NO_2_ and 10%–20% response interval, while data become sparse at both very low (<0.1 ppm) and very high (>500 ppm) concentrations. This strongly right‐skewed data distribution confirms a predominant focus on mid‐range NO_2_ detection in current MXene research, a range that is roughly one to two orders of magnitude (∼10–300 times) higher than the WHO's recommended maximum safe short‐term exposure level. This trend highlights a limitation in MXene‐based sensor technology and suggests the need for data‐driven research focused on improving low‐concentration NO_2_ detection.

To provide a refined representation of the aggregated sensor behavior, Figure 2B presents the meta‐analytic median curve in the range of 0.01 to 50 ppm. The solid red line depicts the smoothed conditional median. For robustness, the dashed lines illustrate the smoothed 25th and 75th percentile response curves, which reflect the inherent heterogeneity and dispersion of the raw data by showing the full range within which the central 50% of observations fall at any given concentration. In this range, the median attains *R_max_* = 31% and reaches half‐maximum at *C_50_* = 0.12 ppm. We benchmarked several parametric forms (Langmuir, Hill–Langmuir, Logistic, Weibull, Freundlich, and Langmuir–Freundlich) against the qGAM median on the same 0.01–50 ppm grid. The three‐parameter Langmuir–Freundlich isotherm provided the best quantitative fit (RMSE = 0.29 percentage points), reproducing the overall shape but with a higher saturation level (*R_max_* = 39%) and a right‐shifted midpoint (*C_50_* = 1/*K* = 0.69 ppm) relative to the nonparametric median. This indicates that a Langmuir–Freundlich isotherm is an adequate approximation to capture the aggregated MXene sensing behavior, as the model describes complex multilayered adsorption and the presence of heterogeneous surfaces with a variety of adsorption sites. The differences between the empirical median curve and the model curve may arise from the variation in the nonparametric median and the presence of additional structural complexity, related to material‐ and study‐level heterogeneity.

The aggregated median curve in Figure 2B confirms the nonlinear relationship inherent to MXene sensor response, with the response rising steeply at low concentrations and saturating at higher ones. At its half‐maximum (*C*_50_ = 0.12 ppm), the local linear slope is 21.9 percentage points per ppm (equivalent to an increase of about 6.0 percentage points for each 10‐fold rise in NO_2_ concentration). This corresponds to an elasticity of 0.17, indicating that a 1% change in concentration produces about a 0.17% change in response near *C*_50_. This highlights the high responsiveness of MXene‐based sensors with unsaturated surfaces in this critical range, particularly for detecting trace levels of NO_2_, where health impacts are significant. As NO_2_ concentration increases, the response gradually plateaus, notably beginning around 10–20 ppm, indicating sensing material surface saturation. This saturation behavior indicates reaching a state of dynamic equilibrium, which depends on a specific surface‐gas interaction mechanism, and may be related to complete or near‐complete occupation of the most energetically favorable adsorption sites on the MXene/MXene‐hybrid surfaces. This defines the practical upper boundary for quantitative sensing applications, as beyond this point only qualitative or semi‐quantitative detection is possible under typical operating conditions.

While the median curve establishes a central tendency, the persistent spread between the 25th and 75th percentile dashed lines serves as a robustness check, confirming that the observed trend is not merely an artifact of the median. This spread further shows the substantial variability among sensors from different studies at each concentration. The sustained dispersion, even post‐aggregation, reinforces the conclusion regarding the inherent heterogeneity in reported performances. It suggests that while MXenes collectively exhibit a strong overall response to NO_2_, specific design choices, material modifications, or experimental conditions profoundly influence the exact shape and magnitude of the response curve, leading to a wide range of observed sensitivities and saturation points. To illustrate this, spearate meta‐analytical sensing response curves for different subgroups of MXene‐based materials are shown in Figure 2C. The figure clearly demonstrates distinct differences in the aggregated sensing response behavior among these subgroups. For instance, pure MXene exhibits a low median response (*R_max_* = 7%) and quickly reaches saturation at *C_50_* = 0.91 ppm, making it the lowest‐performing subgroup in this analysis. In contrast, the meta‐analytical curves for MXenes modified with oxides, organic, and layered non‐sulfide materials exhibit similar saturation trends but significantly higher response levels, reaching *R_max_ *= 36% at *C_50_* = 0.03 ppm for oxide‐modified materials, *R_max_* = 67% at *C_50 _* < 0.01 ppm (below the modeled range) for organic‐modified materials, and an exceptional *R_max_* = 1834% at *C_50_* = 4.9 ppm for layered non‐sulfide materials (a value supported by only two studies and therefore indicative rather than robust).

In contrast to the other subgroups, the meta‐analytical curve for materials modified with layered sulfides is non‐monotonic. After a higher response at very low concentrations, it passes through a shallow minimum at intermediate concentrations before rising again, reaching its half‐maximum only at a comparatively high *C*_50_ = 20.8 ppm and a saturated response of around 52%. While such a trend could reflect a genuine physical behavior that individual studies (typically covering a narrower concentration range) may overlook, the shapes of these subgroup curves must be interpreted with caution wherever the underlying data are sparse. The qGAM smoothing is strongly influenced by noise or single extreme measurements in sparse regions, so non‐monotonic features that are not seen in individual studies may be artifacts of uneven data coverage, which is a limitation that cannot be fully controlled for in the bivariate subgroup analysis.

### 2.2 Key Sensor Performance Metrics

**FIGURE 3:**
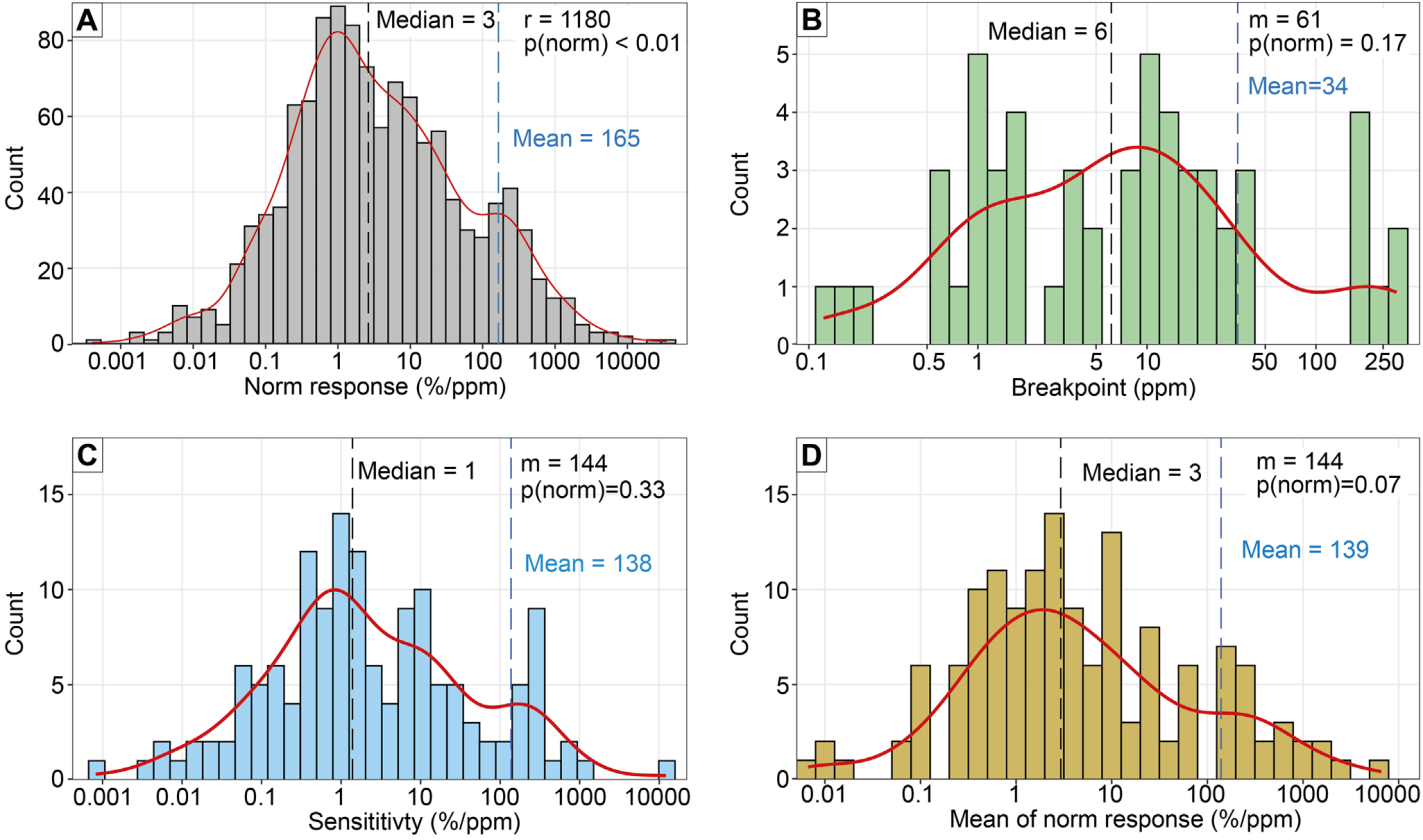
Distributions of the four meta‐analytic effect sizes. (A) Normalized response (record level), (B) Breakpoint (sample level), (C) Sensitivity (sample level), and (D) Mean normalized response (sample level). Dark dashed lines mark medians; blue dashed lines mark arithmetic means. Kernel density curves are overlaid in red for visualization. p(norm) refers to the *p*‐value from a Shapiro–Wilk test of normality performed on log‐transformed data.

To quantitatively characterize sensor performance and enable systematic comparison across studies, we identified four key sensing performance metrics: normalized sensor response, breakpoint, sensitivity (curve slope before breakpoint), and mean normalized sensor response. Figure 3 shows the distribution of these calculated effect size parameters.

Across all records, the normalized response (Figure 3A) spans several orders of magnitude and is strongly right‐skewed, with a mean (165) far exceeding the median (3). This widespread and pronounced positive skew indicates a high prevalence of exceptional sensor outcomes, warranting investigation into whether the published literature disproportionately represents highly successful results. Such a pattern may be consistent with publication selection bias (the file‐drawer problem) [30, 31, 32], whereby the most favorable results are preferentially reported, and the perceived average performance of the technology is thereby inflated.

The distribution of breakpoint values (Figure 3B) is also broad (median = 6 ppm, mean = 34 ppm). A lower breakpoint would imply earlier saturation, whereas a higher breakpoint indicates a wider linear or highly responsive range before saturation, which is generally desirable for practical applications requiring detection across broader concentration ranges. Statistically, the breakpoint variable, when analyzed on a logarithmic scale, is approximately normal (*p* = 0.17), signifying variability in the NO_2_ concentrations at which MXene sensors begin to exhibit saturation or a change in their sensing mechanism. This result is notable because the approximate log‐normality on its own would be unremarkable, as the sensitivity and mean normalized response are also approximately log‐normal (Figure 3); what distinguishes the breakpoint is the comparatively moderate raw‐scale skew (mean/median ratio of about 6, vs. more than 40 for the response‐based metrics), which suggests that researchers report the full, genuine operational range of the sensors rather than only the most favorable saturation points.

The sensor sensitivity (Figure 3C) values also vary widely (median = 1, mean = 138), with a pronounced upper tail pointing to a subset of exceptionally steep response curves. Statistically, the sensitivity variable, when analyzed on a logarithmic scale, behaves as a log‐normal distribution (*p* = 0.33). The right‐skewed nature of the distribution, with a tail extending toward significantly higher values, indicates that while many samples exhibit moderate sensitivity, a notable subset demonstrates exceptionally steep response curves. We cannot rule out some degree of selection bias (e.g., under‐reporting of low sensitivities), but the observed skew is also compatible with genuine cross‐study heterogeneity. To distinguish selective reporting from genuine heterogeneity, we applied regression‐based publication‐bias tests [31, 32]. The FAT–PET scatter plot (Figure S2) shows a mild positive slope. On the raw scale, the regression test indicates a strong small‐study effect (bias coefficient = 17.377, *p* < 0.01) and a mean beyond bias that is essentially zero in magnitude (0.002, *p* < 0.05). The most precise estimates (top 10% lowest standard errors) have a mean of 0.059 on the raw scale, but this decile is selected by absolute precision, which is itself proportional to the estimated sensitivity, so the contrast with the overall mean is not by itself evidence of selective reporting. On the log scale, the bias coefficient is not significant (*p* = 0.75), and the mean beyond bias is modest but positive (0.956, *p* < 0.01). On that scale, the most precise estimates are not smaller than the uncorrected geometric mean (2.767 vs. 2.259), so the small‐study pattern is confined to the raw scale. Including NO_2_ range as a moderator leaves the publication‐bias terms unaffected: none of their interactions with this variable are significant. The bias‐corrected mean is moderated by range, consistent with the saturation documented in Figure 2, since samples tested over wider ranges reach concentrations at which the normalized response is mechanically lower. Taken together, these results indicate that the very large sensitivities observed in some studies, while they may reflect innovative breakthroughs, are not representative of the overall evidence base. The raw‐scale results are consistent with selective reporting, although the log‐scale tests do not indicate it. Full results of the test are presented in Table S1.

For the distribution of the mean normalized response (Figure 3D), the distribution is again right‐skewed (median = 3, mean = 139), consistent with occasional very large pre‐breakpoint responses. The Shapiro–Wilk test indicated approximately normal distribution for the log‐transformed data (*p* = 0.07). The median suggests a typical efficiency level; however, the wide range, particularly the presence of values extending to the higher end of the scale, highlights instances where MXene sensors achieved remarkably high responses relative to the NO_2_ concentration. This metric, similar to the sensitivity, underscores the inherent potential for developing highly efficient MXene‐based sensors.

To better understand the interconnections between key sensing parameters, we further analyzed the relationships between them. Figure S3A shows the dependence of the log sensitivity on the log mean normalized response. The analysis revealed a strong linear fit with a slope of 1.053 (statistically significant at the 1% level), indicating a very strong positive association. This relationship is expected and serves as an additional robustness test for the meta‐analysis. Figure S3B presents the dependence of the log mean normalized response on the log breakpoint, which provides further insights into sensor behavior. In this case, the data are more scattered, and a linear fit yields a slope of −0.535 (statistically significant at the 1% level), indicating a weak negative correlation. While a negative correlation is expected, such a low slope implies that the breakpoint is a less straightforward and more complex parameter, with its variation depending on multiple factors.

Collectively, the wide ranges in these parameters across collected samples underscore that sensor performance is not uniform and is likely influenced by a multitude of underlying material, fabrication, and experimental factors. Identifying these influential factors will be the focus of our subsequent heterogeneity analysis. The right‐skewed distributions are consistent with, though not conclusive evidence of, publication bias, as studies with high‐performing samples may be more likely to be published.

Additionally, the stability slope, response time, recovery time, and relative humidity values were coded, and their distributions (Figure S4) and correlations with the normalized response (Figure S5) were analyzed. It should be noted that, in the case of the stability slope, the number of individual observations was very low, and the time periods over which stability was reported varied significantly across studies (Figure S4A). This insufficiency precluded any meaningful statistical analysis. The stability slope values cluster near zero (median = −0.022, mean = −0.63; Figure S4A), and their correlation with the normalized response is weak and not statistically significant (slope = −0.610, *t* = −1.65; Figure S5A).

For both response and recovery times, while the number of reported observations was substantial (Figure S4B,C), the data quality was insufficient. Many observations represented multiple data points from the same sensor at different gas concentrations. More critically, the methodology for calculating these time intervals was often unspecified or inconsistent. In some papers, sensing response was measured at a fixed time interval regardless of whether the sensor had reached saturation, and recovery time was reported without the signal decreasing to the accepted 10% above baseline.

Despite these data irregularities, the reported values show a similar trend for both response and recovery times: increasing time correlated with a decreased normalized response, showing a strong negative correlation with slopes of −1.416 and −2.113, respectively (Figure S5B,C). In the case of response time, the slope being on the order of −1 may indicate that the sensing response at the saturation point is primarily dependent on the intrinsic characteristics of the sensing materials (e.g., specific surface area, conductivity), sensor construction, and environmental conditions, rather than on the exposure time.

Relative humidity, in contrast, was reported more consistently and shows a modest but statistically significant positive bivariate association with the normalized response (slope = 0.238 per 10%RH, *t* = 1.98; Figures S4D and S5D). Because this is an unadjusted, pairwise association, it does not isolate the humidity effect from correlated design factors. A within‐sample comparison, which removes all between‐sensor differences, shows no systematic humidity effect (Figure S9); this is discussed further in Section 2.7.

Based on the low number of reported stability observations and the significant methodological and reporting inconsistencies observed across all three kinetic parameters, the stability slope, response time, and recovery time were excluded from the subsequent core meta‐analysis.

### 2.3 Subgroup Analysis

Preliminary analysis confirmed significant heterogeneity across the reported results. This variability is expected given the wide range of synthesis methods, material modifications, and measurement conditions employed in the literature. Therefore, a comprehensive moderator subgroup analysis is essential to decompose this heterogeneity and isolate the specific factors driving sensor performance.

**FIGURE 4:**
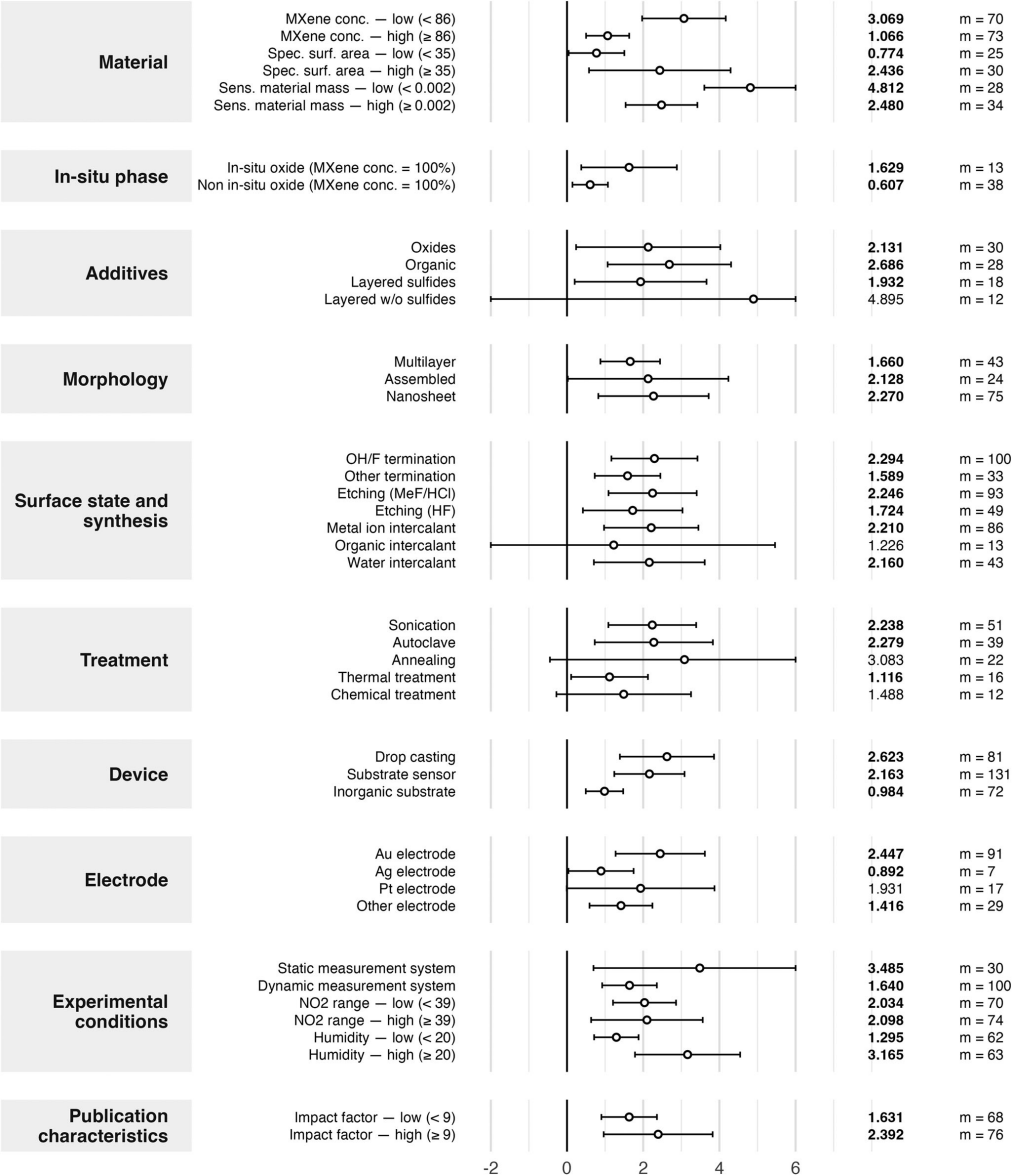
Subgroup analyses for sensor sensitivity. The figure illustrates the estimates of the subgroup means for the natural logarithm of sensor sensitivity (at the sample level). The mean values were calculated using weighted least squares with weights proportional to the number of data points per sample (giving greater influence to observations from larger samples). 95% confidence intervals are truncated to −2 to 6 and calculated with cluster‐robust standard errors that account for within‐study correlations. Subgroups with fewer than three observations were excluded. Estimated mean values are shown next to the graphs. Bold values are statistically significant. Variable descriptions can be found in Table S2.

The results of the subgroup analysis are illustrated in Figure 4 and Figure S6. The values for the outcome variables (normalized response, breakpoint, sensitivity, mean normalized response) were log‐transformed prior to analysis to reduce strong right‐skewness (see Figure 3). Subgroups with fewer than three observations were excluded to avoid unstable estimates. Bolded means indicate subgroup means that differ significantly from zero on the log scale (*p* < 0.05), that is, confidence intervals that do not cross zero. The reported subgroup means reflect weighted averages of log‐transformed input data. These subgroup contrasts are unadjusted bivariate comparisons, several of which rest on few samples; they are therefore descriptive, and independent effects are established in the multivariate meta‐regression (Section 2.5).

#### 2.3.1 Material

Low MXene concentration sensors exhibited a significantly higher mean log‐transformed sensitivity (3.069 vs. 1.066 for high concentration). This pattern was consistent across materials with high specific surface area, which also demonstrated a substantially higher mean log‐transformed sensitivity (2.436 vs. 0.774 for low area) (Figure 4). A similar positive trend was noted for the mean log‐transformed normalized response and mean normalized response in these concentration and specific surface area subgroups: for low versus high MXene concentration, the means were 2.942 and 3.509 versus 1.154 and 1.437, and for high versus low specific area, they were 2.434 and 2.697 versus 1.062 and 1.037, respectively (Figure S6). Considering that gas sensing is a surface‐interaction‐based phenomenon, an increase in the mass of the material on a similar‐sized sensing substrate is expected to increase the thickness of the sensing layer. This can lower the sensing characteristics because the excessive material hinders the access of gas molecules to the surface area and can also potentially suppress charge transfer. This effect is quantified by comparing the log‐transformed means of normalized and mean normalized response values for low material mass (4.617 and 5.416) against high material mass (2.559 and 2.891), respectively. The log‐transformed sensitivity showed a similar effect, with a value of 4.812 for lower material mass sensors versus 2.480 for higher.

On the other hand, the means for the log‐transformed breakpoint show the opposite trend across the MXene concentration and specific surface area subgroups: higher concentration (2.699 vs. 2.311) and lower surface area are associated with higher breakpoints. Sensors with materials with a low specific surface area are characterized by the highest log‐transformed mean breakpoint value among not only these pairs but also one of the highest among all investigated subgroups, at 4.189. For sensing material mass, in contrast, the low‐mass group retains a somewhat higher breakpoint (2.152 vs. 1.652), that is, in the same direction as for sensitivity, although the high‐mass estimate is not statistically significant. Crucially, while numerical differences exist among these breakpoint subgroups, the wide overlap of their confidence intervals suggests that these differences are not robustly differentiated in this bivariate setting. Since a high breakpoint and a high sensitivity are both desirable for maximizing sensor response and sensitivity, the creation of sensors with both these parameters as high as possible is highly desirable. This ambiguity, which may result from factors confounding the simple subgroup comparisons, highlights the necessity of the subsequent multivariate meta‐regression, which can simultaneously test all factors to robustly determine the drivers of the breakpoint.

#### 2.3.2 In Situ Oxidation and Additives

The role of the additives is generally related to an increase in surface area available for adsorption, a change in the value and mechanism of conductivity, and the formation of vacancies and surface‐adsorbed species, like chemisorbed oxygen. Subgroup analysis revealed that the presence of in situ oxides on the surface of pure MXene (MXene concentration is 100%) had a moderate but imprecisely estimated positive effect on sensor performance. Specifically, averaging results across samples shows that in situ oxidized MXene sensors exhibit a higher mean log‐transformed sensitivity (1.629 vs. 0.607) compared to non‐oxidized MXene. This positive association also holds for the log‐transformed normalized response (1.633 vs. 0.653), while the breakpoint is markedly lower (1.072 vs. 3.713; note that the in situ oxide breakpoint estimate is based on only *m* = 5 samples and is not statistically significant), indicating an earlier saturation point. The positive effect on sensing characteristics is typically associated with the enhanced formation of nanoscale TiO_2_/Ti_3_C_2_T_x_ heterojunctions, the presence of electron‐unsaturated dangling Ti bonds, and an increase in oxygen vacancies. The latter serve as electron donors and increase the number of chemisorbed oxygen species. It should be underlined, however, that the effect of the in situ MXene oxidation in the subgroup is restricted to pure MXenes (concentration = 100 wt.%), isolating the effect from theinfluence of other modifiers. The largest effect on sensor sensitivity among modified MXenes was observed with layered non‐sulfide materials. These materials produced the highest log‐transformed sensitivity (mean of 4.895, though not statistically significant), which far exceeded that of organic (2.686), layered sulfides (1.932), and oxides (2.131). The layered non‐sulfides group also showed the highest means for both log‐transformed and mean normalized responses (Figure S6), followed by organic and layered sulfide additives. The breakpoint effect, however, was highest for oxide‐modified MXenes (2.729), while the lowest was for layered non‐sulfides (1.477). It should be noted that the mean values for all parameters in the layered non‐sulfides group had a very high standard error.

#### 2.3.3 Morphology

Analysis of the structural characteristics revealed a direct relationship between morphology and sensor sensitivity. The sensitivity parameter was lowest for multilayered MXene structures (1.660) and highest for nanosheet morphology (2.270). The poor performance of multilayered MXenes is primarily attributed to the limited accessibility of the active surface area for gas molecules and the potential presence of unetched MAX phase or reaction by‐products. Consistent with the sensitivity trend, 2D and 2D‐like morphologies (nanosheets and flakes) exhibited a higher overall sensing response, with nanosheets having log‐transformed means of 2.178 and 2.679 for normalized and mean normalized response. Regarding the breakpoint, nanosheets also produced the highest value (2.917), compared to lower values for multilayered (1.693) and assembled MXenes (1.551).

#### 2.3.4 Surface State and Synthesis

The choice of intercalant led to distinct effects on sensor performance. The presence of a metal intercalant or only water resulted in comparable sensitivity, showing a similar trend to the mean log‐transformed normalized response (2.210 and 2.160, respectively). The sensitivity effect was noticeably lower for organic molecules (1.226). Conversely, organic molecules produced the highest breakpoint (4.696), followed by metal ions (2.147) and water (1.458). It should be noted that the mean values for MXenes with organic intercalants showed a very high standard error for all parameters, limiting the robustness of these findings. The overall performance of metal and water intercalants is comparable to that of native OH/F termination groups, common results of standard synthesis (e.g., the MILD method) that require no additional treatment.

Methods utilizing in situ HF formation from a MeF/HCl mixture generally resulted in higher sensitivity compared to direct HF etching. Specifically, the log‐transformed mean sensitivity was higher for MeF/HCl (2.246) than for HF (1.724). This enhanced sensitivity is supported by higher normalized and mean normalized response values (2.194 and 2.727, respectively, vs. 1.794 and 1.966 for direct HF). However, sensors fabricated with direct HF‐obtained materials exhibited a markedly higher log‐transformed breakpoint (4.270 vs. 2.109 for MeF/HCl). The superior breakpoint of HF‐etched materials may be related to a higher probability of forming structural defects.

#### 2.3.5 Treatment

Regarding treatment techniques, annealing resulted in the highest log‐transformed mean sensitivity (3.083, though this estimate is not statistically significant) among all techniques, which is likely due to the formation of in situ oxides and a more uniform surface. This high sensitivity is supported by the highest values for normalized response (2.881) and mean normalized response (3.661). However, the wide variety of annealing parameters (temperature, atmosphere, time) leads to a high standard error (the distribution of the mean normalized response value over annealing temperature is shown in Figure S7). While high sensitivity is achieved, annealing decreases the breakpoint (2.391), compared, for instance, to chemical treatment (4.137), thereby limiting the effective concentration range of these sensors. Conversely, autoclave treatment provided an optimal balance of high sensitivity (2.279) and a substantial breakpoint (3.515), which, combined with strong normalized (2.504) and mean normalized responses (2.630), indicates its suitability for the modification of MXenes with other nanomaterials. In contrast, thermal treatments other than annealing (e.g., plasma, laser) and chemical treatment generally had a weaker impact compared to annealing, showing markedly lower log‐transformed normalized and mean normalized responses and sensitivity.

#### 2.3.6 Device, Electrode, Experimental Conditions

On the device level, the choice of electrode material is critical for maximizing sensitivity. Analysis showed that sensors with gold electrodes demonstrated the highest sensitivity (2.447) among all materials, which is supported by the highest overall normalized and mean normalized response (2.482 and 2.898, respectively). This performance surpasses that of platinum (sensitivity: 1.931, normalized response: 2.008), silver (sensitivity: 1.029, normalized response: 0.877), and other electrode materials (sensitivity: 1.416, normalized response: 1.539), though the platinum and silver subgroups are small (*m* = 17 and *m* = 7) and the platinum sensitivity estimate is not statistically significant. Gold electrodes also exhibited a relatively high breakpoint (2.538), making them an excellent choice for sensors requiring high sensitivity over a broad range of NO_2_ concentrations.

Experimental conditions also appear as influential factors. Statistical analysis shows that higher humidity is associated with higher log‐transformed means for sensitivity compared to low humidity (3.165 vs. 1.295), with similar trends for normalized response and mean normalized response (2.924 vs. 1.406, and 3.671 vs. 1.643, respectively), while the values for breakpoint are comparable (2.344 vs. 2.760). Humidity has a well‐known effect on the sensing response of semiconductor gas sensors and is commonly associated with changing surface charge distribution via proton conduction (Grotthuss mechanisms) or/and oxygen ion displacement. At the same time, this effect is generally considered parasitic, as humidity hinders true sensing response and requires careful consideration during sensor practical application; the positive bivariate association observed here therefore likely reflects, at least in part, confounding with correlated design factors, and a within‐sample comparison confirms that it does not persist once between‐sensor differences are removed (Figure S9; Section 2.7).

Finally, the type of measurement setup significantly impacts the measured sensitivity. Static measurements yielded substantially higher log‐transformed means for the sensitivity parameter (3.485) compared to dynamic measurements (1.640). This enhanced sensitivity is mirrored in the normalized response and mean normalized response (static: 3.268 and 3.974; dynamic: 1.687 and 1.994). Although the breakpoint was comparable (static: 2.481 vs. dynamic: 2.504), the vast difference in measured sensitivity and response between the two setup types indicates that experimental configuration is a major source of variance. Under ideal conditions, the setup should not affect the measured sensor parameters and should be designed to exclude external factors like gas flow. However, due to a lack of standardization and if the setup is poorly designed, these external effects may not be accounted for and can influence the measurements.

#### 2.3.7 Publication Characteristics

The log‐normalized sensitivity in journals with an impact factor equal to or above the median impact factor of all journals in the sample is 2.392 as compared to the sensitivity observed from lower‐impact journals (1.631). This difference suggests that the quality or visibility of the publication outlet, as measured by impact factor, is associated with the reported sensor performance, as studies with higher‐performing sensors tend to be published in journals with a higher impact factor. This also indirectly supports the possibility of publication bias (Figure 3 and the publication‐bias tests in Figure S2 and Table S1) with a general tendency to report and accept studies with higher‐performing sensors for publication.

Overall, it was revealed that lower MXene concentration, higher surface area, and lower sensing materials mass are crucial for achieving improved sensor response and sensitivity. The type of additive plays a substantial role, with layered non‐sulfide materials showing the largest, though statistically not robust, overall effect on sensing response and sensitivity. 2D or 2D‐like nanosheet morphology generally produced a high sensing response, similar to the positive effects of metal and water intercalants, and OH/F termination groups. In terms of processing, annealing was found to be the most effective treatment for enhancing sensing response and sensitivity; though its estimates were not statistically significant, it tended to decrease the breakpoint and was associated with a high distribution of results, possibly due to a wide variety of annealing parameters. Autoclave treatment offered a good balance of high sensing response, breakpoint, and sensitivity. At the device level, sensors with gold electrodes consistently demonstrated the highest performance in terms of sensing response and sensitivity. Finally, the measurement setup itself was found to be a variable, with static measurements yielding higher response values than dynamic ones, underscoring the need for more standardized experimental protocols in the field.

### 2.4 Detailed Analysis

Due to the high complexity and variability of the materials compositions used in sensors, detailed statistical analysis of their impact on sensor performance can reveal additional trends and provide important insights for selecting the most effective material formulations. First, Figure S8 shows the distribution of shares of different modifier groups used in published papers over time. The use of modified MXenes increased markedly from 0% in 2020 to 63% in 2021, peaking at 100% in 2022, before decreasing to 47% in 2024. Clear differences between modifier classes could also be seen. Oxide modifiers dominated from 2021 to 2024, reaching their highest share in 2022, whereas in 2025 the research focus shifted toward organic‐modified MXenes.

**FIGURE 5:**
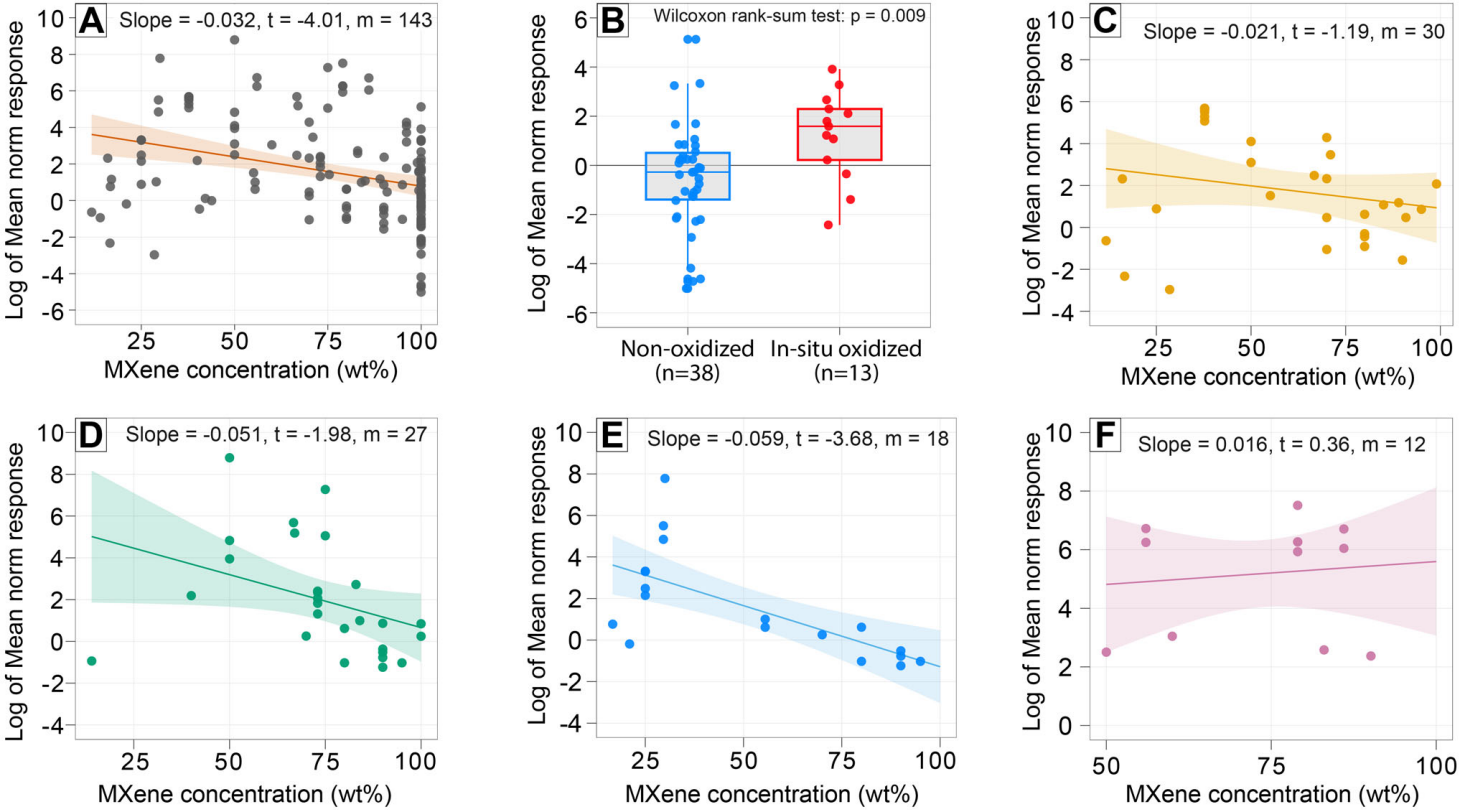
The effect of sensing material composition on the mean normalized response. (A) Logarithm of the mean normalized response per sample versus MXene concentration (all modifiers). Points are individual samples. Solid lines show ordinary least‐squares fits with 95% CIs. Legend boxes report the OLS slope, *t*‐statistic, and sample size. (B) Comparison of the two groups of sensing materials at 100 wt.% with and without in situ oxidation using a Wilcoxon rank‐sum test. Logarithm of the mean normalized response per sample versus MXene concentration with (C) metal oxides, (D) organic materials, (E) layered sulfides, and (F) layered non‐sulfides as modifiers.

Figure 5 illustrates the effect of sensing material composition on the mean normalized response, allowing for an assessment of how continuous composition features influence sensing characteristics. As shown in Figure 5A, by performing a linear fit of the combined data from all samples, we reveal a trend: the mean normalized response decreases as MXene concentration increases, with a significant fitted slope of −0.032 (*t* = −4.01). This finding suggests that overall, pure MXene has a lower sensing response compared to modified MXene composites.

A more detailed analysis of specific subgroups confirms this. The formation of in situ oxides on the MXene surface in the case of the nonmodified MXene (MXene concentration is 100%) clearly increases the mean normalized response (Figure 5B). This finding, though related to the beneficial effects of composite materials, differs in that in situ oxide formation represents an intrinsic surface modification of the base MXene, while the materials discussed here pertain to extrinsic additives in a composite. For samples modified with metal oxides, organic materials, and layered sulfides, the mean normalized response likewise declines as MXene concentration rises—that is, the additive‐rich compositions perform better—with fitted slopes of −0.021, −0.051, and −0.059, respectively (Figure 5C–E). Of these, only the layered‐sulfide trend is statistically significant (*t* = −3.68); the organic trend is marginal (*t* = −1.98), and the oxide trend is not significant (*t* = −1.19). For layered non‐sulfides, by contrast, the fitted slope is slightly positive (0.016) but not statistically significant (*t* = 0.36), so no reliable concentration trend can be inferred for this subgroup (Figure 5F). It is important to note, however, that the effect of modifiers is complex. For example, semiconductor oxides like WO_3_, In_2_O_3_, and ZnO are known for their high sensing response at elevated temperatures. Therefore, using MXene‐oxide composites at room temperature may degrade their properties and result only in a marginal performance increase, but these composites could be highly useful for applications requiring elevated temperatures. Additionally, while an increase in the concentration of layered non‐sulfides shows no clear dependence of the mean normalized response on concentration, these composites as a whole still show a higher sensing response than pure MXenes. The observation that the mean performance of a composite group exceeds the mean performance of pure MXenes, despite a negative bivariate concentration trend, is an indication of confounding. This necessity to isolate the pure additive effect from other factors validates the subsequent use of the multivariate meta‐regression in the next section.

Overall, the presented data indicate that introducing additives generally plays a positive role in increasing the mean normalized response of MXene‐based gas sensors. However, the choice of a specific modifier and its concentration must be carefully considered, as an increase in MXene concentration often leads to a decrease in sensor response. These results also suggest a complex role for MXene materials in the overall sensing mechanism. Beyond acting as matrices for more sensitive components, MXenes contribute to gas molecule adsorption and charge accumulation and transfer, highlighting their multifunctional role in the sensing process.

**FIGURE 6:**
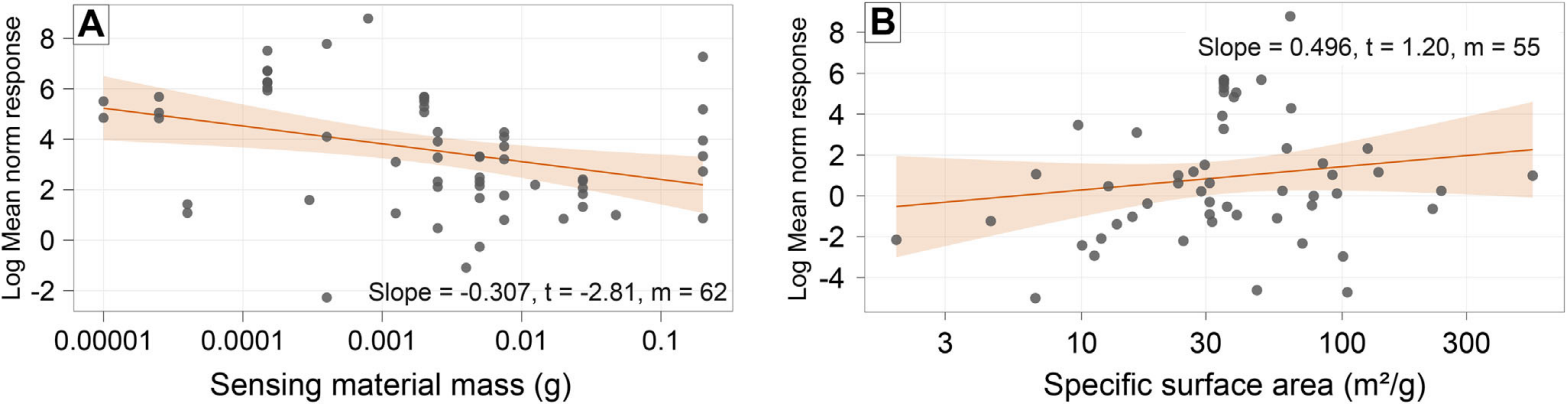
Natural logarithm of the mean normalized response per sample versus material properties. (A) Sensing material mass (g). (B) Specific surface area (m^2^/g). Points are individual samples. For samples with a breakpoint before saturation, the mean is computed from responses below the breakpoint. The solid line is an ordinary least‐squares fit of log(response) on log(*x*) with a 95% confidence band.

The results of a detailed investigation of the effects of sensing material mass and specific surface area on the sensing response are presented in Figure 6.

From the presented data, it can be seen that for both parameters there is high variance in mean normalized response across changing material mass (Figure 6A) and specific surface area (Figure 6B). However, a linear fit of the data allows us to see general trends. In the case of material mass, mean normalized response values in general tend to decrease along with mass increase, with a significant slope of the fitted line of −0.307 (*t* = −2.81). As it was mentioned before, the observed trend may be related to a decreasing available surface for gas molecule adsorption with increasing mass and possible suppression of the charge transfer. In the case of the surface area, its increase leads to an increase in mean normalized response with the slope of the fitted line of 0.496, although this trend is not statistically significant (*t* = 1.20), which is directionally consistent with an important role of the surface area available for gas adsorption in the sensing mechanism.

### 2.5 Meta‐Regression Analysis and Bayesian Model Averaging

The primary BMA and meta‐regression analyses were applied to the log‐normalized response, our only outcome variable at the record level. To balance the number of observations with the number of moderators, we excluded moderator variables with a high degree of missing data. Specifically, we dropped specific surface area, sensing material mass, and inorganic substrate. Additionally, we intentionally excluded experimental condition variables such as NO_2_ range and humidity; the role of humidity is examined separately in Section 2.7 (Figure S9). Because silver electrodes were reported too infrequently to support a stable separate estimate, they were merged into the other electrode category. The substrate sensor dummy was removed entirely due to near‐zero variation within the estimation sample. These exclusions resulted in a final sample of 756 sensor responses from 35 studies, which represents approximately 64% of the analytic record set.

**FIGURE 7:**
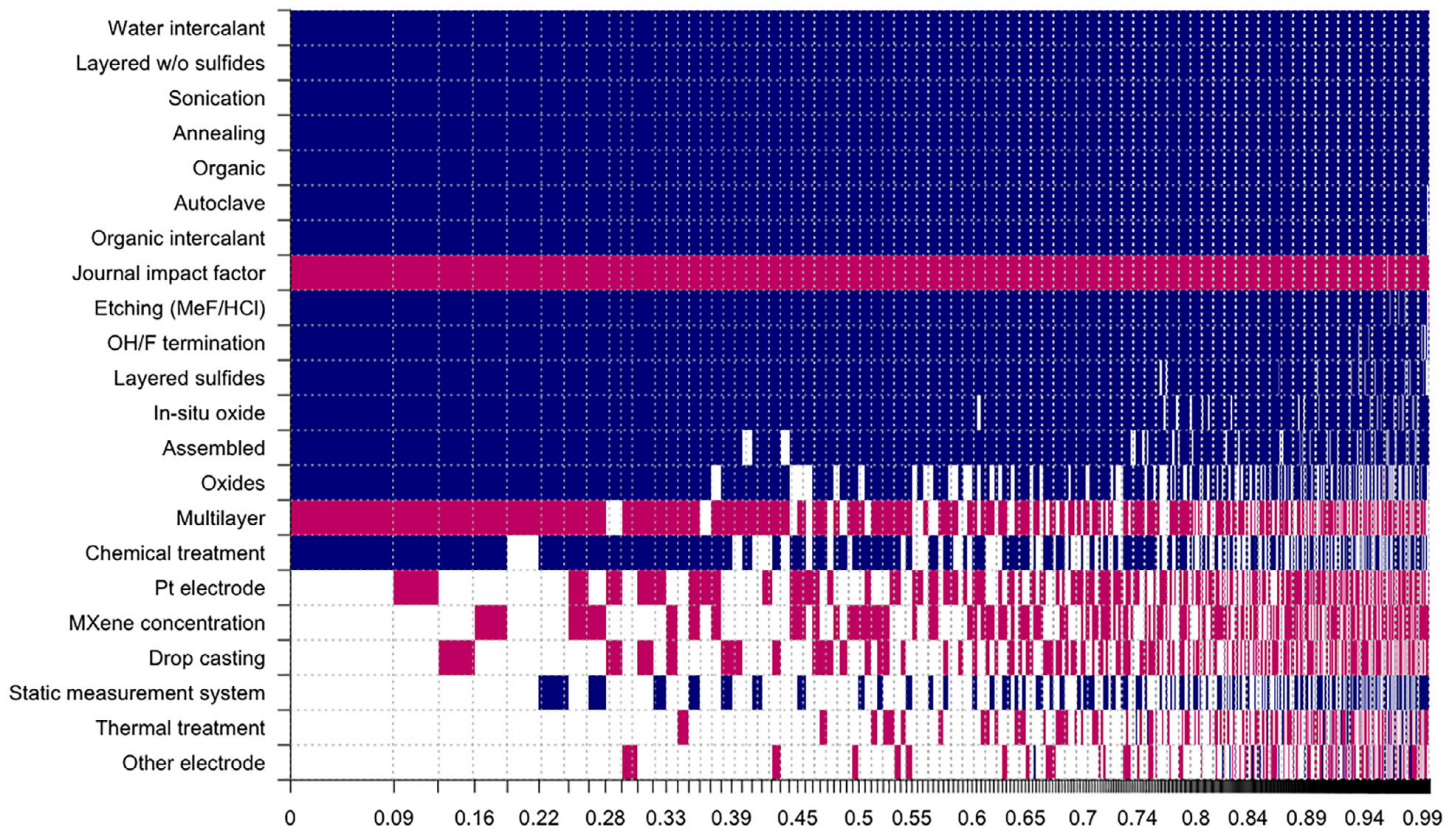
Bayesian model averaging heatmap. The figure shows the results of the Bayesian model averaging to identify the key drivers of heterogeneity among sensor responses (record level). The columns represent the individual models, with the log of the sensor response normalized by the NO_2_ level as the response variable. The moderator variables (explanatory variables) are sorted by their inclusion probability in descending order, indicating the likelihood of their inclusion in the model. The horizontal axis shows the cumulative posterior model probabilities, which are a measure of the models’ fit. Models on the left are of higher fit. The cells highlighted in blue indicate the model inclusion of a variable with a positive sign, while those in pink indicate the inclusion of a variable with a negative sign. Uncolored cells indicate that a variable is not included in the respective model. The model averaging is based on the unit information g‐prior and the dilution prior, which takes collinearity among the moderators into account. The number of observations is 756 from 35 studies. Variable definitions can be found in Table S2.

Figure 7 shows the BMA results. It is a heatmap where each row represents a moderator variable, and each column represents a regression model. The rows are sorted by their posterior inclusion probability (*PIP*), with the most relevant variables listed at the top. The columns are ordered by their posterior model probability, with the best‐fitting models appearing on the left. Cells are colored to depict the sign of the variable's coefficient: blue indicates a positive sign, while pink denotes a negative sign. Blank cells represent variables not included in a model.

In all categorical moderator groups with more than two levels (e.g., morphology, intercalants, electrodes), the model applies a dummy‐variable specification in which one category is omitted and serves as the baseline (see Table S2). Consequently, the estimated coefficients for the included categories quantify their effects relative to that reference group rather than relative to each other. Thus, for example, the coefficients for multilayer and assembled MXenes are interpreted as their effects compared to nanosheets, and the intercalant coefficients as effects relative to metal‐ion intercalation.

The BMA heatmap shows that a core set of moderators is consistently included in the highest‐probability models, indicating that these variables robustly explain the heterogeneity in sensor performance. Across the most probable regression models, the signs of their coefficients remain stable, confirming that these effects are not model‐dependent. The variables with the highest posterior inclusion probabilities (*PIP*s) predominantly relate to surface state (in situ oxide, OH/F termination, organic and water intercalants), additives (oxides, layered sulfides, and especially layered materials without sulfides), post‐synthesis treatments (sonication, autoclave, and annealing), MXene morphology (assembled and multilayer structures), and the MXene synthesis route (etching with MeF/HCl). Together, these factors form the set of predictors identified by the BMA and account for a substantial share of the observed variation in normalized sensor response.

The numerical results of our meta‐regression analysis are presented in Table 1. This table contains three distinct models. Model 1 shows the numerical BMA results of Figure 7, including the posterior mean, posterior standard deviation, and *PIP* for each moderator. To interpret our results, we use established cut‐off levels for *PIP*. Following Jeffreys [33] and Raftery [34], we consider evidence to be decisive if the *PIP* exceeds 0.99, strong if it is greater than 0.95, and positive if the *PIP* is above 0.75. To ensure robustness, we also present two alternative specifications as frequentist checks. Model 2 displays the results from an OLS meta‐regression, and Model 3 presents the results from a WLS meta‐regression; both are estimated with standard errors clustered at the study level. Both frequentist models include only the variables identified by BMA with a *PIP* greater than 0.5. WLS applies weights proportional to the number of data points per sample and humidity condition, giving greater influence to sensor–condition combinations supported by more measurements.

**TABLE 1:** Heterogeneity analysis of sensor responses via Bayesian Model Averaging (record level).

Response variable: *Log of normalized response*	(1) Bayesian model averaging			(2) OLS meta‐regression			(3) WLS meta‐regression		
	Post. mean	Post. SD	*PIP*	Coef.	*t*‐stat.	*p*‐value	Coef.	*t*‐stat.	*p*‐value
*Constant*	−3.108	NA	1	−3.651	−2.02	0.05	−4.050	−2.32	0.03
*Material*									
MXene concentration	−0.003	<0.01	0.39	—	—	—	—	—	—
*In situ phase*									
In situ oxide	1.230	0.42	0.97	1.193	1.72	0.10	1.223	1.55	0.13
*Additives*									
Oxides	0.654	0.45	0.78	0.979	1.41	0.17	1.470	1.73	0.09
Organic	2.342	0.42	1	2.586	2.28	0.03	3.707	3.22	<0.01
Layered sulfides	1.901	0.61	0.98	2.479	1.80	0.08	2.361	1.88	0.07
Layered w/o sulfides	4.436	0.39	1	4.583	7.19	<0.01	4.584	9.59	<0.01
*Morphology*									
Multilayer	−0.576	0.41	0.76	−0.922	−1.05	0.30	−0.968	−1.07	0.29
Assembled	0.960	0.40	0.94	0.884	0.87	0.39	0.401	0.42	0.68
*Surface state and synthesis*									
OH/F termination	0.908	0.24	0.99	1.061	1.59	0.12	1.153	1.91	0.06
Etching (MeF/HCl)	2.496	0.66	1	2.616	1.47	0.15	3.244	1.94	0.06
Organic intercalant	2.491	0.53	1	2.668	1.99	0.06	2.632	2.10	0.04
Water intercalant	4.141	0.66	1	4.239	2.30	0.03	4.960	2.73	0.01
*Treatment*									
Sonication	1.442	0.27	1	1.319	1.48	0.15	0.703	0.80	0.43
Autoclave	1.180	0.26	1	1.148	1.38	0.18	0.794	0.89	0.38
Annealing	1.344	0.27	1	1.267	2.48	0.02	1.047	2.12	0.04
Thermal treatment	−0.099	0.33	0.20	—	—	—	—	—	—
Chemical treatment	0.531	0.43	0.71	0.790	1.45	0.16	0.998	1.55	0.13
*Device*									
Drop casting	−0.130	0.22	0.35	—	—	—	—	—	—
*Electrode*									
Pt electrode	−0.315	0.43	0.45	—	—	—	—	—	—
Other electrodes (incl. Ag)	−0.019	0.10	0.16	—	—	—	—	—	—
*Experimental conditions*									
Static measurement system	0.129	0.26	0.29	—	—	—	—	—	—
*Publication characteristics*									
Journal impact factor	−0.073	0.02	1	−0.076	−1.70	0.10	−0.066	−1.58	0.12
*r*(No. of records)	756			756			756		
*n* (No. of studies)	35			35			35		

*Note*: This table presents the numerical results for the BMA (Figure 7). Post. Mean = Posterior Mean, Post. SD = Posterior standard deviation, *PIP* = Posterior inclusion probability, Coef. = Coefficient. The ordinary least squares (OLS) and weighted least squares (WLS) meta‐regressions are estimated using standard errors clustered at the study level. WLS applies weights proportional to the number of data points per sample and humidity condition (giving greater influence to sensor–condition combinations supported by more measurements). The OLS and WLS regressions include only the variables recognized by BMA with a *PIP* > 0.5. Variables are described in Table S2.

The BMA analysis reveals that 12 moderators (excluding the constant) show strong or decisive evidence of influencing the log‐normalized response (*PIP* > 0.95), indicating that these factors play a central role in explaining the variation in sensor performance. These moderators consistently appear in the most probable regression models, exhibit stable coefficient signs, and are corroborated in direction and magnitude by the OLS and WLS checks. These frequentist models are estimated with study‐clustered standard errors and are thus deliberately conservative; where individual coefficients fall short of conventional significance under clustering, the posterior evidence and the stability of signs across specifications remain the primary basis for inference.

#### 2.5.1 In Situ Oxidation and Additives

The choice of material modification (whether intrinsic or extrinsic) emerges as the most decisive driver of sensor performance in the multivariate analysis. Layered non‐sulfide materials exhibit the strongest positive effect among all categories (posterior mean of 4.436, *PIP* = 1). This translates to a predicted response increase of roughly 98‐fold (exp(4.584)) in the robust WLS model relative to sensors without a layered non‐sulfide additive, confirming that heterojunctions with these materials are a highly effective strategy; this effect remains highly significant in every specification. Organic additives are similarly powerful (posterior mean of 2.342, *PIP* = 1) and statistically significant in both frequentist models. The effect size is substantial, with the WLS coefficient (3.707) implying a 41‐fold enhancement. Layered sulfides (*PIP *= 0.98) and oxides (*PIP* = 0.78) also show positive effects. Layered sulfides remain significant at the 10% level in both frequentist checks, whereas oxides do so only in the WLS specification, indicating that while generally beneficial, their consistency across different study conditions is lower than for the top‐tier additives. Moreover, in situ oxidation retains a strong positive association (posterior mean of 1.230, *PIP* = 0.97), representing a roughly 3.4‐fold increase in response, though at marginal significance in the frequentist checks. However, the explanatory power of this regression finding is limited in the case of in situ oxidation, as the study of the effect was exclusively restricted to sensing materials with 100% MXene concentration.

#### 2.5.2 Morphology

The structural form of the MXene remains relevant, though less dominant than compositional factors. The multilayer morphology exhibits a negative correlation with sensor performance (posterior mean of −0.576, *PIP* = 0.76) when compared to the reference 2D nanosheet morphology. In contrast, the effect of the assembled morphology is positive (posterior mean of 0.960, *PIP* = 0.94). These results quantitatively support the concept that enhanced sensor performance is achieved through the effective exfoliation of the bulk MXene accordion structure, followed by its subsequent 3D assembly. However, a nuance emerges in the robustness checks: neither morphological coefficient reaches conventional significance under study‐clustered inference, so while the posterior evidence supports assembled structures outperforming multilayer MXenes, the magnitude of this benefit is more study‐dependent than the compositional effects above.

#### 2.5.3 Surface State and Synthesis

The analysis highlights that specific synthesis protocols and the resulting surface chemistry are far more influential than bulk material properties. The etching method (MeF/HCl) emerged as a decisive factor (*PIP* = 1) as compared to HF etching. The WLS coefficient indicates that this etching protocol is associated with a 26‐fold higher response compared to HF etching, likely due to the resulting surface chemistry. Consistent with this, OH/F surface terminations show a robust positive effect (*PIP* = 0.99), further emphasizing the role of surface chemistry. Conversely, MXene concentration is found to be a less influential factor (*PIP* = 0.39) in the multivariate model, implying that once the effects of surface chemistry and additives are accounted for, the raw concentration of MXene is less critical.

#### 2.5.4 Intercalation and Treatment

Intercalation and post‐synthesis treatments are among the most powerful determinants of performance identified. Water intercalants show a strong, robust positive effect (posterior mean of 4.141, *PIP* = 1). The WLS coefficient suggests an extraordinary increase in response of 143× relative to metal‐ion‐intercalated samples (the reference category), confirming the vital role of adsorbed water in gas interaction with the sensing surface and charge transfer. Additionally, the presence of water may lead to surface oxidation over time. Organic intercalants are also a top‐tier factor (posterior mean of 2.491, *PIP* = 1), with a significant positive effect in the WLS model and marginal significance in OLS (*p* = 0.06), corresponding to a roughly 14‐fold performance boost over the metal‐ion reference. Among post‐synthesis treatments, annealing is the most robust (*PIP* = 1), significant in both the OLS and WLS models. Other treatments like sonication (*PIP* = 1) and autoclave (*PIP* = 1) receive decisive posterior support, though their coefficients do not reach significance under clustering, which is consistent with effectiveness that varies with protocol details such as treatment time, temperature, and pressure.

#### 2.5.5 Device, Electrode, Experimental Conditions

In contrast to the bivariate subgroup analysis (Figure 4), the multivariate model reveals that device architecture and measurement conditions are not key drivers of sensor performance when controlling for material properties. The measurement system (static vs. dynamic), which appeared as a relevant factor in the bivariate analysis, has a low posterior inclusion probability (*PIP* = 0.29) and was not selected in the final regression models, suggesting its apparent influence was confounded by other study characteristics. Similarly, sensing element production method (e.g., drop casting) (*PIP* = 0.35) and most electrode materials do not appear as relevant factors in the multivariate setting. The only exception is Pt electrodes, which show a moderate negative association (posterior mean of −0.315, *PIP* = 0.45) compared to Au, though this effect is not decisive. This overall pattern suggests that high sensor performance is driven more by the sensing material's intrinsic chemistry and processing than by the device assembly or measurement setup.

#### 2.5.6 Publication Characteristics

The journal impact factor shows a consistent negative association (*PIP* = 1), suggesting that studies published in higher‐impact journals report lower normalized responses after controlling for all other factors, in notable contrast to the positive bivariate impact‐factor pattern in the subgroup analysis (Figure 4), which reverses once material and synthesis factors are held constant. This finding serves as a proxy for methodological rigor and possibly for the selective placement of high‐performing results. Higher‐impact journals likely enforce more stringent testing protocols and require greater validation, which filters out optimistic artifacts and leads to more conservative estimates. The result is stable in magnitude across all specifications, and its persistence with a consistent sign in every model, together with a *PIP* of 1, marks it as a robust pattern rather than a specification artifact.

The stability of our core findings was tested through two sensitivity analyses. Figure S10 demonstrates that the implied relative importance of the core set of influential explanatory variables remains stable across alternative BMA prior specifications, confirming that the dominance of additives and intercalants is driven by the data rather than prior model assumptions. Furthermore, Figure S11 assesses robustness using a maximized dataset of 1170 observations from 55 studies. This model achieves a larger sample size by excluding variables with incomplete records (primarily the detailed synthesis and surface chemistry markers, for example, specific etching agents or termination types). In this coarser but broader model, the signs and high *PIP*s for the key additives, intercalants, and publication factors remain consistent, reinforcing the validity of our main conclusions. However, a notable deviation is observed for MXene concentration and generic thermal treatment, which appear more influential in this reduced‐variable specification. This suggests that when detailed controls for synthesis quality (such as specific etching protocols) are unavailable, these broader material parameters act as proxies for material quality, whereas in the fully specified model, the precise compositional factors are the true drivers of performance. Finally, it is important to acknowledge that these variations may also be driven by the different, broader database itself, which includes a wider array of studies with diverse experimental conditions that were excluded from the primary analysis.

### 2.6 Best Practice Estimates

To move beyond simply identifying the most influential factors, we can use our meta‐regression model as a predictive tool to estimate the implied effects of specific design choices. This approach allows us to quantify the practical significance of key moderator variables by comparing sensor performance under different hypothetical configurations.

The method involves first defining a hypothetical reference sensor configuration by setting each influential moderator variable to a specific baseline level. Based on the WLS model selection (Table 1), the reference configuration serving as our high‐performance benchmark is configured as follows:

- **Material and additives**: In situ oxide is set to 1 (on). Layered non‐sulfide materials are set to 1 (on), while other additives (oxides, organic, layered sulfides) are set to 0. Note that, by construction, the in situ oxide variable is defined only for samples with 100 wt.% MXene (Table S2), so this particular combination should be read as a stylized reference point rather than an observed configuration; because the model is linear in logs without interaction terms, each contrast below equals exp(*β*) for the switched variable and is invariant to the values at which the remaining moderators are held.
- **Morphology**: Assembled morphology is set to 1 (on), while multilayer is set to 0. Nanosheets are the omitted baseline category.
- **Surface and synthesis**: OH/F termination is set to 1 (on), and MeF/HCl etching is set to 1 (on). Water intercalant is set to 1 (on), while organic intercalants are set to 0, and metal ion is the omitted baseline category.
- **Treatment**: Sonication, autoclave, annealing, and chemical treatments are all set to 1 (on).
- **Publication**: The journal impact factor is held constant at the sample median.

**FIGURE 8:**
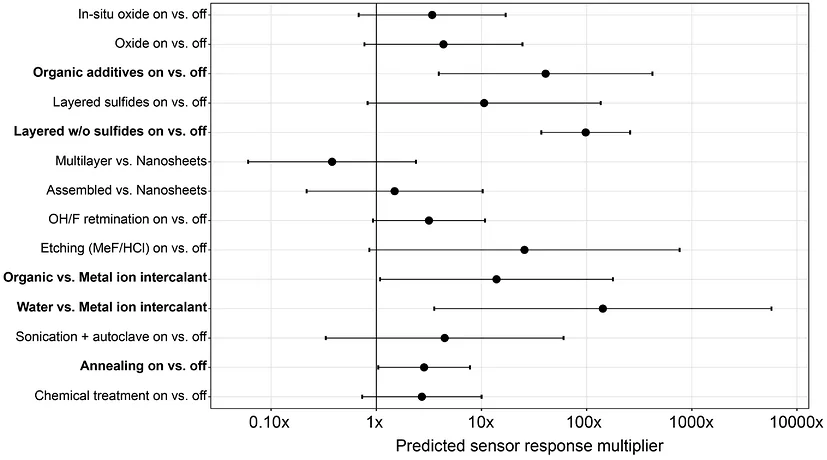
Predicted normalized responses assuming best‐practice sample conditions. Points show model‐based contrasts in predicted sensor response (normalized by NO_2_ concentration) from the WLS meta‐regression in Table 1. Each row compares the predicted sensor response from two models; the multiplier is the ratio of the predicted response for the model shown to the left of “versus” divided by the response to the right. This ratio is calculated by taking the exponent of the difference between the two log‐transformed predicted responses. A multiplier > 1× indicates a higher predicted response for the left‐hand configuration, while 1× denotes no difference. Horizontal lines are 95% confidence intervals, using cluster‐robust (study‐level) standard errors. Predictions hold all other moderators at the ‘best‐practice’ values. Variables in bold are statistically significant at the 5% level.

From this benchmark, we systematically vary a single moderator at a time to create a series of contrasts, each representing a different hypothetical experimental setup. This allows us to quantify the practical significance of our findings. The results are shown in Figure 8, which visualizes the multiplicative effects, or ratios, of specific design choices on the predicted sensor response. This method works by calculating the log difference between two predicted responses and then exponentiating this value to get the predicted response multiplier. The vertical line at 1× represents the null effect, indicating no difference between the two configurations. Multipliers greater than 1× indicate a positive effect, meaning the modified configuration is predicted to have a higher sensor response, while multipliers less than 1× indicate a negative effect. The horizontal lines represent the 95% confidence intervals; if an interval does not cross the vertical line at 1×, the effect is considered statistically significant. For example, if the point for a contrast like organic additives on versus off is at around 41×, it means a sensor with organic additives is predicted to have a response that is 41 times higher than a sensor without them, holding all other variables constant at the values explained above. Because each contrast isolates the effect of switching a single category while holding all other moderators fixed at their baseline levels, contrasts involving multi‐level variables (morphology and intercalants) should be interpreted as comparisons against their omitted reference group (i.e., nanosheets or metal‐ion intercalants), rather than as direct comparisons between the non‐baseline categories themselves. Five contrasts are statistically significant at the 5% level under study‐clustered inference: organic additives, layered non‐sulfide additives, organic and water intercalation, and annealing.

The results of this analysis reveal that synthesis and modification strategies are the most powerful levers for enhancing performance, vastly outperforming morphological adjustments.

The biggest performance gains are predicted for specific additive classes. Layered non‐sulfide materials offer a massive boost, with a predicted multiplier of roughly 98× compared to otherwise identical sensors without them. Organic additives also show a substantial practical impact, with a predicted 41× increase in response. The presence of in situ oxide shows a consistent positive role; contrasting the on versus off state reveals a multiplier of roughly 3.4×. In all three cases, the increase in sensing performance is commonly associated with improved electron transfer between components of the sensor's surface and adsorbed gas. The electron donor components, however, differ from case to case. Furthermore, it is commonly believed that such composition modifications increase the concentration of surface adsorbed oxygen radicals, which plays a crucial role in NO_2_ oxidation [35, 36, 37].

Specific synthesis protocols provide the next tier of performance enhancements. The use of MeF/HCl etching is predicted to yield a response 26× higher than alternative (HF) etching methods. We propose that the pronounced contrast in sensing response may be related to the surface chemistry of the obtained materials, specifically the presence of Me ions on the MXene surface acting as a potential source of free electrons. Water intercalants are predicted to offer a pronounced 143× improvement over metal ion intercalants, while organic intercalants yield a predicted 14× improvement. In both cases, the intercalation offers better access of the gas molecules to the surface of the MXene flakes; however, we believe that the main factor could be associated with improved electron transfer due to partial auto‐oxidation and providing additional surface functionality [38]. This is in line with the effect of OH/F surface terminations, which provide a further enhancement of roughly 3.2×.

While less dominant than the chemical factors, morphological choices remain practically relevant, though not statistically distinguishable in this framework. Assembled morphology is predicted to yield a response roughly 1.5× higher than nanosheets, whereas multilayer stacks are predicted to underperform (0.38×). Combined treatments of sonication and autoclave result in a predicted 4.5× increase, while annealing contributes a roughly 2.8× improvement, the latter being statistically significant. These results indicate that increasing surface area (sonication), electron transfer (assembled morphology), and surface oxidation/activation (sonication, autoclave, and annealing) are important, easy‐to‐perform techniques that can be used to improve the performance of the MXene gas sensors.

Although the subgroup comparisons (Sections 2.3 and 2.4) are more immediately intuitive, we treat the multivariate model as the statistically stronger basis for inference because it accounts for covariation among factors and yields more reliable effect estimates. In contrast to previous subgroup analyses, device parameters and bulk concentration are not primary differentiators in this multivariate framework. Instead, these best‐practice estimates suggest that the path to highly efficient MXene‐based NO_2_ sensors lies in precise chemical engineering, specifically through water/organic intercalation and the creation of heterojunctions with layered non‐sulfide or organic additives.

To ensure these predictive insights are not artifacts of the weighting scheme, we replicated the prediction analysis using the standard OLS model (Figure S12). While the exact magnitudes of the multipliers vary, typically being more conservative for some factors in the OLS specification (e.g., water intercalation: 70× vs. = 143×), the qualitative conclusions remain identical. In the OLS specification, four contrasts remain statistically significant (organic additives, layered non‐sulfide additives, water intercalation, and annealing), while organic intercalation falls just short of the 5% threshold. The dominance of the additive and intercalant effects is therefore robust across both specifications, although the wider intervals in the unweighted model leave the organic‐intercalant contrast less certain.

In conclusion, these best‐practice estimates implied from the meta‐regression model offer an actionable summary of our meta‐analysis findings, translating the statistical coefficients into tangible performance improvements, providing a data‐driven blueprint for designing and fabricating highly efficient MXene‐based NO_2_ gas sensors. However, given the current constraints of the aggregated literature, the recommendations for highly specialized application scenarios, such as wearable electronics or specific industrial waste gas monitoring, are currently not possible. Consequently, the optimized configurations proposed here should be viewed as general performance guidelines; delineating the specific design requirements for these niche operational environments remains a task for future research.

### 2.7 Limitations

Unlike meta‐analysis in disciplines such as medicine, psychology, economics, or business, our unit of analysis is an experimental measurement rather than a statistical estimate with a sampling variance, so the standard error entering the FAT‐PET/PEESE tests (Table S1) is a proxy from sample‐level regression fits, and the funnel‐type diagnostics should be read as indicative rather than formal. The plausible selection mechanism also differs from the significance‐driven selection these tests were built for [31, 32], which in economics, empirical finance, and the social sciences more broadly operates through *p*‐value‐based selection and low statistical power [39, 40, 41]. Engineering studies tend to report their best‐performing samples, selecting on effect magnitude rather than *p*‐values, a mechanism only recently formalized [42]. The skewness we document is consistent with such magnitude‐based reporting, but also with genuine cross‐design heterogeneity, which our tests cannot separate.

The framework also assumes a log‐linear outcome with additive moderator effects. The log transform is strongly supported by the distributional evidence (Section 2.2), but the proportionality embedded in the normalized response is not itself relaxed, and interactions such as additive‐by‐humidity remain beyond the current evidence base. The moderators are observational codings of design choices, and unobserved study‐level factors such as laboratory skill may correlate with both choices and outcomes. BMA and study‐level clustering address model uncertainty and within‐study dependence, but not causal identification [27]. Accordingly, we read the journal‐impact‐factor coefficient as a proxy for study rigor and/or the selective placement of high‐performing results rather than a causal venue effect, and the within‐sample comparison in Figure S9, which isolates humidity within individual sensors, is the closest our data come to an internally controlled contrast. Consistent with this, the positive bivariate association between humidity and response does not persist within sensors: after demeaning within sample and adjusting for NO_2_ concentration, the humidity slope is −0.009 per 10%RH and is not statistically significant. The apparent effect is therefore carried by differences between sensors (that is, by which sensors were tested under humid conditions) rather than by a within‐sensor response to increasing humidity, and should be read as a confounded between‐study signal rather than a causal moderator.

Sample‐level power is a further constraint. The breakpoint, sensitivity, and mean‐normalized‐response outcomes are defined per sample rather than per record, and several subgroup contrasts rest on a few samples. These estimates are therefore reported as descriptive, with independent effects established only where the multivariate analysis concurs.

The best‐practice benchmark (Section 2.6) combines settings that are individually well supported but jointly sparse; in situ oxide, in particular, is defined only for 100 wt.% MXene samples, making the full configuration a stylized reference point. Because the model is linear in logs, each multiplier equals exp(*β*) for a single switched factor and is baseline‐invariant, so the multipliers should be read as one‐factor‐at‐a‐time contrasts within the observed support, not predictions for jointly untested configurations. Finally, our quantitative conclusions apply to Ti_3_C_2_T*_x_*‐based sensors at room temperature (20°C–30°C). We do not extend these empirical findings to other MXene chemistries, whose distinct surface terminations and electronic structure may alter the response in ways our data cannot capture (the studies identified for those systems are listed in Table S4). The temperature window precludes statements about heated or sub‐room‐temperature operation, and the kinetic metrics (response time, recovery time, and the stability slope) are reported descriptively rather than modeled, because their measurement and reporting conventions vary too widely across studies to support meta‐regression. Selectivity is not synthesized quantitatively at all. The analytical framework and reporting recommendations, however, are not chemistry‐specific and apply to gas‐sensor performance synthesis more broadly. As all inputs are published results, systematic errors shared across the literature, such as common conventions for extracting responses from figures, would propagate to our estimates.

## 3 Conclusion

This study performed the first comprehensive meta‐analysis of peer‐reviewed scientific papers on MXene‐based room‐temperature NO_2_ gas sensors. Given the relative novelty of MXene as a class of sensing materials, the available literature is limited in scope and highly varied in research approaches, material modifications, treatment methods, and sensor designs. This variety creates a significant challenge in defining the most promising research directions and best technological practices. We addressed this by conducting a rigorous, data‐driven analysis of the available literature, providing actionable guidance for future sensor development.

Based on our analysis, we defined normalized sensing response, breakpoint, sensitivity, and mean normalized sensing response as the key parameters for characterizing sensor quality, as these can be consistently derived from the reported measurement data across studies. Our systematic analysis identified 32 explanatory variables that varied widely across studies. Using statistical and meta‐regression analysis, we isolated drivers responsible for massive, orders‐of‐magnitude changes in performance. The results consistently indicate that the most influential factors are defined by materials’ composition and surface chemistry: specific additives (layered non‐sulfides, organics), synthesis approach (etching protocols, surface terminations), and intercalants (water, organics). In contrast, device‐level parameters such as electrode material were found to be statistically overshadowed and thus secondary drivers of performance.

We also identified patterns consistent with selective reporting. The distribution of reported sensitivities is heavily skewed on the raw scale and shows a pronounced small‐study effect in the regression‐based bias tests on that scale, in line with a tendency to report exceptional, high‐performance results, although genuine cross‐design heterogeneity likely contributes as well. The distribution of saturation points (breakpoints), by contrast, is only moderately skewed. This divergence suggests that selective reporting, if present, concerns primarily the headline performance metrics, whereas the physical saturation limits of the sensor materials appear to be documented more evenhandedly across the literature.

Based on our analysis, we move beyond simple recommendations to propose a data‐driven blueprint for development, focusing on the factors with the strongest statistical support for high performance:

- **Modification**: rioritize MXene‐based materials modified with layered non‐sulfide and organic additives, as these materials demonstrate high surface area, favorable conductivity, and the highest positive effects in the meta‐regression.
- **Synthesis**: Optimize the synthesis process by utilizing etching methods based on in situ HF formation (MeF/HCl—MILD method) instead of direct HF etching, or develop and apply new methods for the effective etching of the MAX phase.
- **Treatment**: Apply combined post‐synthesis treatments (e.g., sonication, followed by autoclaving or annealing) to achieve optimal exfoliation and controlled assembly of the MXene structure.
- **Surface activation**: Strategically employ methods that promote water intercalation and controlled in situ oxidation of the MXene surface to enhance surface reactivity and the number of active sites.
- **Morphology**: Target 3D‐assembled MXene architectures to maximize the exposed specific surface area and facilitate efficient gas diffusion and rapid adsorption/desorption kinetics.

These research directions are not exhaustive but serve as a guideline. Some other potential directions, however, can be excluded based on our findings. For instance, while oxide modifiers show a positive effect, they are statistically less robust and consistent than organic or layered non‐sulfide alternatives. Thermal treatments other than annealing (such as plasma or laser) showed negligible effects in the multivariate analysis, indicating that these complex and expensive modifications do not yield a noticeable improvement over established routes. In contrast, while chemical treatments demonstrated a directionally positive effect, their impact was highly imprecise and less consistent than the leading alternatives. Furthermore, it was shown that although the electrode material may influence sensing properties, it is not a determining factor. Thus, it is important to note that specific material choices and engineering solutions should be adapted to the particular needs of the application. For example, due to the high cost of Pt and Au, Ag electrodes could be a good alternative for prototyping or for sensors where exceptionally high sensitivity is not a primary requirement, while using Au and Pt may be considered for gaining some additional sensitivity and corrosion stability for specific applications.

By analyzing a wide and complex variety of parameters and moderator variables, this study identified several critical research gaps and opportunities for enhancing reporting practices in the field of MXene‐based NO_2_ sensors and gas sensors in general.

- **Low‐concentration NO_2_ detection**: Most of the research focuses on sensing characteristics in a concentration range of 10–300 times higher than the maximum safe NO_2_ concentrations defined by the WHO. This focus limits the potential application of these sensors for air quality control, confining them primarily to industrial or technological process monitoring. A key gap exists in developing sensors capable of detecting concentrations below 1 ppm.
- **Exploring MXenes beyond Ti_3_C_2_T*_x_***: While Ti_3_C_2_T*_x_* is the most common MXene material due to its simple synthesis and stability, other MXenes or combinations of different MXenes have the potential to outperform it as sensing materials.
- **Novel materials and compositions**: A related research gap is the development of new MXene‐based hybrid and composite materials. Combining MXenes with other phases can create synergetic effects that enable enhanced sensing performance beyond pure MXenes or MXenes with known modifiers.
- **Low‐temperature sensing performance**: The sensing characteristics of MXene‐based NO_2_ sensors have been studied almost exclusively at room temperature and, in some cases, at elevated temperatures. This leaves a significant gap in understanding their behavior at temperatures below room temperature and, particularly, below the freezing point of water.
- **Alternative sensing principles**: Only a few studies report on sensors operating on physical principles other than the direct or indirect measurement of resistance changes. Exploring other physical effects and principles holds significant potential for advancing the sensing concentration range, detection time, recovery time, and selectivity of these devices.
- **Multigas and multifunctional sensors**: Due to the relative novelty of this field, there is a clear lack of research on the development of multigas sensing platforms and multifunctional sensors that can monitor several environmental parameters. These would be highly valuable for civil and technological applications.
- **Standardized measurement protocols**: Despite significant research efforts, there is no standardized approach for sensor characterization, making a direct comparison of published results and experiment replication more challenging. This lack of standardization applies not only to setup configurations but also to the measurement of response time, stability, and selectivity. The development of a standardized framework for sensing measurements would be highly beneficial to the field. For instance, we suggest that full response/recovery profiles, with sensors reaching apparent full saturation and recovery, should be reported. The selectivity, as well as stability, should be reported as full response/recovery profiles for a selected broad range of concentrations and gas mixtures. The latter is important for the assessment of the sensor performance for real‐world applications.

We also propose several improvements to current results reporting practices, which would enhance data comparison, replication, and facilitate future research:

- **Report all data, not just the best**: Authors should report data for all samples investigated, not just the best‐performing ones. This practice would provide a more complete understanding of research results and help avoid unnecessary experiment replication.
- **Include key material parameters**: It is crucial to report the specific surface area and sensing material mass of the samples. These are fundamental parameters for comparing the characteristics of different materials and the sensors based on them.
- **Full saturation data**: Reported sensing time intervals are often cut short of the saturation point. Reporting sensing and recovery times at or near full saturation would allow for a more accurate assessment and comparison of sensor characteristics across different studies.
- **Publish full data sets**: Publishing a full set of raw data, methods for data treatment, and final processed data is a good practice for all scientific research. It fosters progress, enhances communication among researchers, and increases trust in published results.

This meta‐analysis delivers a comprehensive, data‐driven assessment of the current state‐of‐the‐art in MXene‐based NO_2_ gas sensors. By systematically identifying performance trends, knowledge gaps, and critical deficiencies in reporting practices, it establishes clear priorities for future research and standardization. Beyond consolidating the field, this work provides a robust methodological framework that is transferable to other emerging materials systems, enabling more reproducible, comparable, and application‐relevant sensor research across disciplines.

## 4 Methodology

### 4.1 Literature Search

The data collection process followed standard guidelines for meta‐analysis [26, 28, 29]. At first, a systematic search of the Web of Science database was conducted using a combination of (“gas sensor” OR “detection” OR “sensing” OR “monitoring”) AND (“NO_2_” OR “nitrogen dioxide”) AND (“MXene” OR “Ti_3_C_2_”) as our keyword string. The search yielded 199 records after unrelated entries (e.g., studies focused on SnO_2_ or non‐gas sensing applications) were excluded. An additional four records were identified by inspecting the reference list of a recent review article by Reddy and Aich [7]. After removing duplicates, 203 unique articles remained for screening. Based on their titles and abstracts, 111 records were excluded as irrelevant (e.g., studies focused on non‐MXenes, etc.), leaving 92 full‐text articles to be assessed for eligibility. From these full‐text articles, studies were selected based on the following inclusion/exclusion criteria:

- **Material specification**: Only studies focusing on the MXene type Ti_3_C_2_T_x_ were included. This restriction ensured a homogeneous dataset, as Ti_3_C_2_T*_x_* is the most common and well‐characterized MXene for gas sensing, while the number of papers available on other types was too small to facilitate a meaningful, cross‐study comparison of performance metrics for different MXenes.
- **Target gas**: Only records detecting NO_2_ were included. This choice minimized heterogeneity arising from different redox and adsorption mechanisms across analytes. NO_2_ was also one of the most frequently studied oxidizing gas for MXenes, with widely used exposure limits and test protocols that support consistent benchmarking of performance metrics.
- **Sensitivity/response type**: Studies had to report sensitivity or responses in terms of resistance, current, or voltage. These parameters are directly related through Ohm's law and are, therefore, comparable for quantitative analysis. Studies that used other physical principles, such as frequency changes [43], could not be directly compared with those based on electrical parameters. Due to the limited number of such papers, their inclusion would have made a meaningful statistical comparison impossible.
- **Operating temperature**: Only records reporting sensing response measured at room temperature (<30°C) were included. This restriction minimizes heterogeneity of the data set, originating from a wide range of operating temperature regimes, and is justified by the fact that room‐temperature operation is a defining feature of MXene‐based sensors, and the majority of the reported studies were performed at room temperature.
- **Study type**: To be included in the analysis, publications were required to be original, peer‐reviewed articles reporting primary experimental data. Consequently, all non‐experimental works, such as descriptive review articles and computational studies, were excluded.
- **Comparability**: Studies reporting contradictory data, using inconsistent measurement scales, or with MXene concentrations below 10% were excluded. The concentration threshold ensured that the observed sensing effect could be primarily attributed to the MXene material.

Following these criteria, 61 studies published up to October 3, 2025, were retained for the meta‐analysis (Figure S13, Table S3). Studies excluded at the full‐text stage on material grounds are listed in Table S4. These cover the main alternative MXene systems reported for NO_2_ sensing, including Ti_2_CT*_x_*, Mo_2_CT*_x_*, Nb_2_CT*_x_*, and V_2_CT*_x_*, none of which was represented by more than three studies, with several further systems appearing in a single study only. The data coded from these studies were structured hierarchically on three levels: studies, samples, and records (Figure S14). A study referred to a single published article. Within each study, one or more samples were reported, where a sample was a specific sensing material with unique properties. Each sample, in turn, provided one or more records, where a record was a single measurement of sensor performance at a specific combination of operating conditions (e.g., NO_2_ concentration, humidity, temperature). In total, the 61 studies provide 195 samples and 1243 records.

### 4.2 Data Extraction and Standardization

The meta‐analysis data were manually extracted from tables and text within the primary studies. When response data were presented only graphically, PlotDigitizer (https://plotdigitizer.com/app) was used for extraction. Additionally, some data were retrieved directly from the Supporting Information. For one study [44], data were obtained directly from the authors.

Following the data extraction, the dataset was systematically standardized to ensure the consistency and comparability of all data points. All reported NO_2_ concentrations were converted to parts per million (ppm), and the sensing response was recalculated where necessary, as detailed below. To ensure comparability and minimize temperature‐induced performance variations, the analysis was restricted to sensors operating at room temperature without external heating, that is, data recorded at 18°C–30°C. The sensing response (*S*) for all studies was standardized to the percentage change in resistance, calculated as:

(1) $$S\left(\%\right)=\left(R_{g}-R_{a}\right)/R_{a\;}\times 100,$$

where *R_a_* represents the sensor's resistance in air, and *R_g_* denotes its resistance in the tested gas environment. When studies reported the response using different formulas, the values were converted to conform to Equation (1). For example, responses reported as a direct ratio (*S* = *R_g_*/*R_a_*) were recalculated using the formula *S*(%) = (*S* − 1) × 100.

When the MXene content was not explicitly stated, it was calculated based on the sample synthesis descriptions. For studies involving quantum dot‐modified MXenes, where MXene content was not calculable, we assumed a 99% MXene content, under the assumption that the quantum dots represent a minor surface modification and that MXene constitutes the bulk of the active material. In the case of in situ oxides, the oxide was classified as present only if it was intentionally obtained during material synthesis or post‐treatment. Conversely, MXene‐derived oxide materials, lacking a distinct MXene crystalline phase, were excluded. For three records, where sensing response was not specified in the text, and data reading from the figures was impossible due to image scale and low value for the respective sensors, the corresponding responses were set to 0.0001, a value chosen to be below the smallest reported response in the sample. Because the analysis uses a log‐transformed outcome, we verified that removing these three records leaves the meta‐regression coefficients in Table 1 unchanged in sign and significance.

When synthesis methods, material treatments, or sensor characteristics were not directly described but were referenced as analogous to those previously reported in another study, the referenced study was cross‐checked to extract the relevant details.

### 4.3 Effect Sizes

In a meta‐analysis, an effect size is a standardized metric that quantifies the magnitude of a phenomenon, allowing findings from different studies to be directly compared and synthesized [45]. From the raw sensor response data, we derived seven effect sizes to capture distinct aspects of performance: normalized sensor response, breakpoint (saturation point) (Figure S15A), sensitivity (slope) (Figure S15B), mean normalized response (pre‐breakpoint average response), stability slope, response time, and recovery time. Of these, the first four (normalized response, breakpoint, sensitivity, and mean normalized response) form the core of the analysis, while the stability slope, response time, and recovery time were retained for additional analyses.

#### 4.3.1 Normalized Sensor Response

This effect size was calculated by dividing each standardized *S* by its corresponding NO_2_ concentration. The resulting normalized sensor response is a measure of sensitivity (% response per ppm (%/ppm)) that enables a direct comparison of sensor efficiency across different studies and concentration ranges.

#### 4.3.2 Breakpoint

The breakpoint identifies a critical NO_2_ concentration where the sensor's response behavior transitions toward saturation. Breakpoints were estimated using a segmented (piecewise linear) bivariate regression model with an initial guess at the mean NO_2_ concentration. A breakpoint was accepted if

- the segmented model provided a statistically significant improvement over a simple linear model (ANOVA, *p* < 0.05),
- the estimated breakpoint fell within the observed NO_2_ concentration range of the sample, and
- at least three data points were available below the estimated breakpoint.

If no valid breakpoint meeting these criteria could be identified (e.g., too few total points, non‐significant segmented fit, breakpoint outside observed range, or insufficient points below it), the breakpoint value was recorded as missing. Fits whose points below the breakpoint are numerically collinear were also rejected (relative standard error below 10^−^^6^), because a zero‐residual fit reflects digitization rounding rather than genuine measurement precision. Samples for which no slope could be estimated, and samples whose estimated slope was negative, were excluded from the sample‐level analyses, since a negative response–concentration slope is not interpretable as a sensitivity under the response definition in Equation (1). Together with the outlier exclusion in Section 4.4, this reduces the 195 coded samples to the 144 samples reported in Table S2.

#### 4.3.3 Sensitivity

The sensitivity quantifies the change in sensing response of a sensor over changing NO_2_ concentration. For samples with a valid breakpoint, the sensitivity was estimated as a slope from a bivariate regression fitted to the data below the breakpoint. If no valid breakpoint was identified, the sensitivity was instead taken from a simple linear regression fitted to the entire response curve. A higher sensitivity indicates greater specific sensitivity to NO_2_ concentration changes.

#### 4.3.4 Mean Normalized Response

This effect size reflects the average magnitude of the sensor's response within its primary operational range. For samples with a valid breakpoint, it was calculated as the mean of the normalized sensor responses below the breakpoint. For samples without a breakpoint, it was calculated as the mean across all observed concentrations.

#### 4.3.5 Stability Slope

This variable reflects the sensor's endurance and reliability over time. It was quantified based on the total change in sensing response at the specific NO_2_ concentration over time reported in the study. A lower slope absolute value indicates better long‐term performance and robustness over time. The stability slope was calculated as a linear fit of the reported data on sensing response at a fixed NO_2_ concentration over time in days.

#### 4.3.6 Response Time

The response time quantifies the speed at which the sensor reaches a stable response after the introduction of the target gas. The reported response time was converted into seconds for all studies. A lower response time indicates faster detection capabilities. It should be mentioned, however, that the protocols and reporting conventions for the response time are rarely specified and may vary significantly across studies.

#### 4.3.7 Recovery Time

The recovery time quantifies the speed at which the sensor returns to its initial state after the target gas has been removed. The reported recovery time was converted into seconds for all studies. A lower recovery time indicates faster recovery and readiness for subsequent measurements. The protocols and reporting conventions for the recovery time are rarely specified and may vary significantly across studies.

### 4.4 Outlier Analysis

As a crucial preliminary step to ensure the robustness of our findings, we conducted an outlier analysis. The presence of outliers, while potentially representing genuinely high‐performing sensors, can disproportionately skew summary statistics and meta‐regression analysis. Based on a qualitative assessment of highly anomalous behavior and inconsistencies with underlying physical mechanisms, 63 records from 5 studies [46, 47, 48, 49, 50] were identified as highly influential outliers and subsequently excluded from further analysis. The respective authors confirmed that these responses most likely reflect gas‐flow effects arising from the measurement setup rather than the intrinsic characteristics of the sensing materials. Figure S16 presents boxplots illustrating the distribution of these key sensor metrics, with observations from the five omitted studies in blue.

### 4.5 Moderator Variables

The vast and methodologically diverse body of literature on MXene‐based NO_2_ sensors necessitates a systematic approach to identify the study characteristics that explain variations in reported performance. Thirty‐two explanatory variables were identified for the analysis. These variables were manually extracted from the primary studies and through cross‐referencing cited publications for missing details. Many of these study characteristics are categorical. These are therefore represented as dummy variables, where a value of 1 indicates the presence of a specific feature, and 0 indicates its absence. If there is no information given on the presence or absence of a factor, it is coded as ‘NA’.

Table S2 provides a list of variables and summary statistics, including the mean, standard deviation, and number of observations for each. The distribution of the categorical moderator variables across individual samples is visualized in Figure S17.

#### 4.5.1 Material

This group describes basic characteristics of the sensing material, including the percentage of MXene in the sensing composite (wt.%), its specific surface area (m^2^/g), and the mass of sensing material (g).

#### 4.5.2 In Situ Oxide

A binary variable indicating whether an in situ oxide phase was intentionally formed during synthesis or post‐treatment. For the current meta‐analysis, sampling was limited to the cases where the MXene concentration is 100 wt.%.

#### 4.5.3 Additives

These variables record whether oxides, organic components, or sulfides were added to the sensing material. Layered additives are further distinguished as layered sulfides or layered non‐sulfides.

#### 4.5.4 Morphology

This category captures the macroscopic morphology of MXene, distinguishing multilayer, assembled, and nanosheet forms.

#### 4.5.5 Surface State and Synthesis

Given the key role of surface chemistry in MXene sensing, this group records whether OH/F termination was present, the etching method used (MeF/HCl vs. HF), and the type of intercalant applied for delamination (metal‐ion, organic, or water only).

#### 4.5.6 Treatment

Post‐synthesis modifications of the material or sensor are represented here, including sonication, autoclave treatment, annealing, thermal treatment, and chemical treatment.

#### 4.5.7 Device

This category specifies aspects of sensor fabrication, such as whether drop casting was used, if the sensor was substrate‐based, and whether the substrate was inorganic.

#### 4.5.8 Electrode

This group identifies the type of electrode materials employed (gold, silver, platinum, or other). For multi‐material electrodes, grouping was determined by the higher element content when proportions were specified. If proportions were not specified, the electrode was grouped by the material listed first. In the multivariate meta‐regression and Bayesian model averaging, the silver‐electrode category was merged into “other” owing to its small number of samples; it is reported separately only in the bivariate subgroup analysis.

#### 4.5.9 Experimental Conditions

These variables describe study‐specific operating conditions, including whether the measurement system was static or dynamic, the tested NO_2_ concentration range, and the relative humidity during measurement. Operating temperature was coded but is not used as a moderator, since the inclusion criteria restrict the sample to <30°C.

#### 4.5.10 Publication Characteristics

This category records the journal impact factor of the publication, serving as a proxy for study quality and visibility, using the Web of Science Journal Citation Reports (JCR) Impact Factor for the journal in the paper's year of publication; for papers published in 2025, we used the 2024 JCR Impact Factor because 2025 scores were not yet available.

### 4.6 Subgroup Analysis

To better understand which of the explanatory variables (also referred to as moderator variables) from Table S2 drive differences in observed sensor performance across samples, we apply subgroup analyses across the four outcome variables (normalized response, breakpoint, sensitivity, and mean of normalized response). The subgroup mean values were derived using weighted least squares (WLS) regression. WLS estimates the mean effect for each subgroup, using a weighting scheme that gives greater influence to samples supported by a larger number of measurements [51]. 95% confidence intervals were calculated using cluster‐robust standard errors (CR2) to account for within‐study dependence. The results of this analysis provide preliminary insights into bivariate effects by comparing sensor performance across single moderator categories.

### 4.7 Meta‐Regression Analysis and Bayesian Model Averaging

Subgroup meta‐analysis provides insights into bivariate effects by comparing sensor performance across single moderator categories. Therefore, it cannot account for the simultaneous influence of multiple factors. To analyze the impact of the various moderator variables from Table S2 on the sensor performance while disentangling these complex and interconnected relationships among the moderators, we employ meta‐regression analysis [52].

The primary meta‐regression and BMA were applied to the log‐transformed normalized sensor response, our only outcome variable at the record level (*r* = 1180). After restricting to records with complete data on all included moderators, the estimation sample comprises 756 records from 35 studies (Section 2.5). The meta‐regression framework models the variation in the record‐level outcome according to the following general equation:

(2) $$Y_{\mathrm{ij}}=\beta_{0}+\sum_{k=1}^{K}\beta_{k}\;X_{\mathrm{ijk}}+\varepsilon_{\mathrm{ij}}$$

where *Y_ij_* denotes the log‐transformed normalized response *i* reported in study *j*. The vector *X_ijk_* represents the set of *K* moderator variables for that observation, and *ε_ij_* represents the error term. The intercept, *β_0_*, reflects the mean log‐transformed normalized response when all moderators are zero or at their reference levels (for categorical variables). The coefficients, *β_k_*, quantify how specific study characteristics or contextual factors influence the reported sensor response.

Ideally, we would regress the sensor responses on all moderators *X* described in Table S2. However, given the large number of regressors, it is highly probable that many variables are redundant. This compromises the precision of parameter estimates for the more important regression variables, leading to model uncertainty and overly optimistic confidence intervals [53]. To address this, we follow the established best practice approach of Bayesian Model Averaging (BMA) to identify the most robust and influential moderators [25, 27, 54, 55]. For this, we used the bms package for R by Zeugner & Feldkircher [56], which walks only through the most likely models. Given *K* moderator variables, the model space comprises 2*^K^* regressions, each consisting of a unique subset of the explanatory variables. The method then computes weighted averages of the estimated coefficients, known as posterior means, across all these models. The weighting is based on posterior model probabilities, which reflect each model's fit to the data. This process yields two key outputs for each moderator: the posterior mean and the *PIP*. The *PIP* indicates the posterior probability that a given moderator belongs in the model, serving as a measure of its importance. A *PIP* value close to 1 suggests a variable has high explanatory power, while a value near 0 indicates low relevance.

### 4.8 Model Specification

In traditional meta‐analysis, weighting is typically based on the inverse variance of the effect size to give greater influence to more precise estimates [51, 57]. As this study analyzes individual experimental measurements rather than statistical estimates, there is no variance for each observed sensor response. Therefore, we use the number of data points per sample at a given humidity condition (i.e., the number of sensor‐response records sharing the same sample and relative‐humidity level), giving greater influence to more densely measured sensor–condition combinations. This assumes that effect sizes derived from sensor–condition combinations with a larger number of measurements are more reliable and thus should be given a greater weight in the meta‐regression.

To account for the fact that multiple samples originate from the same study, we follow an established best practice in meta‐regression analysis [53, 55, 58, 59, 60] and apply cluster‐robust standard errors and 95% confidence intervals around the estimated mean values, thus correcting for potential within‐study dependencies. This procedure provides more reliable statistical inference by correcting for intra‐study correlation [61, 62].

## Author Contributions

**Andreas Rathgeber**: supervision, writing – review and editing. **Alexander Khort**: conceptualization, data curation, formal analysis, writing – original draft, methodology, investigation, funding acquisition, visualization. **Jerome Geyer‐klingeberg**: conceptualization, methodology, data curation, investigation, formal analysis, visualization, writing – original draft. **Suelen Barg**: supervision, writing – review and editing, resources, funding acquisition.

## Conflicts of Interest

The authors declare no conflicts of interest.

## Supporting information




**Supporting File**: advs77530‐sup‐0001‐SuppMat.pdf.

## Data Availability

The data that support the findings of this study are available at https://myweb.rz.uni‐augsburg.de/~geyerkje/research%20files/MXene‐Data.xlsx.
