# Supplementary material for: MXene‐Based Room‐Temperature NO2 Gas Sensors: A Meta‐Analysis
Source: Adv Sci (Weinh). 2026 Sep 10:e77530. Online ahead of print. doi: 10.1002/advs.77530 (PMC13562839; doi:10.1002/advs.77530)
Supplement: Supplementary file 1 [file ADVS-9999-e77530-s001.pdf]

Supplementary Materials for

MXene-Based Room-Temperature NO_2 Gas Sensors: a Meta-Analysis

Alexander Khort[a], Jerome Geyer-Klingeberg[b], Andreas Rathgeber[b], Suelen Barg[*b]

[a] *Department of Chemistry, KTH Royal Institute of Technology, Stockholm, Sweden*

[b] *Institute of Materials Resource Management, University of Augsburg, Augsburg, Germany*

** Corresponding author: SB, email: suelen.barg@uni-a.de*

List of contents

Table S1. Publication bias tests
Table S2. Definition and summary statistics of variables
Table S3. List of primary studies
Table S4. List of studies identified for MXene systems other than $Ti_3C_2T_x$

Figure S1. Distribution of sensor responses per NO_2 concentration bin
Figure S2. FAT–PET meta-regression scatter plot for sensor sensitivity
Figure S3. Relationship among effect sizes
Figure S4. Distributions of additional record-level parameters
Figure S5. Correlations of additional parameters with the normalized response
Figure S6. Subgroup analyses across outcome variables
Figure S7. Relationship between annealing temperature and the log of the mean normalized response
Figure S8. Distribution of modifiers over time
Figure S9. Within-sensor humidity effect
Figure S10. Robustness test of Bayesian Model Averaging with alternative priors
Figure S11. Bayesian Model Averaging heatmap for moderators without missing values
Figure S12. Predicted normalized responses assuming best-practice conditions using OLS results
Figure S13. PRISMA flow chart of the literature search process
Figure S14. Hierarchical structure of our metadata set
Figure S15. Breakpoint identification and sensitivity estimation
Figure S16. Outlier analysis for key sensor metrics
Figure S17. Distribution of categorical moderator variables in the sample

References cited in Table S3. List of primary studies
Additional references cited in the supplement

Table S1. Publication bias tests

	(1) Raw scale	(2) Log scale
Panel A. FAT-PET		
Bias-corrected mean: β_0	0.002**	0.956***
	(2.03)	(4.05)
Publication bias: β_1	17.377***	1.791
	(8.23)	(0.32)
Panel B. PEESE		
Bias-corrected mean: γ_0	0.006	0.997***
	(1.10)	(3.81)
Publication bias: γ_1	0.012	-1.458
	(0.47)	(-0.48)
Mean sensitivity without bias correction	137.721	2.259
Mean sensitivity of top 10% precision	0.059	2.767
m (No. of samples)	144	144
n (No. of studies)	53	53

Complementing the FAT–PET scatter plot in Figure S2, this table reports regression-based tests of publication bias. Panel A shows FAT–PET regressions (*z* on *precision*) for the level model $Sensitivity = \beta_0 + \beta_1 \cdot SE$, where the intercept ($\beta_1$) indicates small-study effects (publication bias) and the coefficient on precision (β_0) estimates the bias-corrected mean effect. Panel B shows PEESE regressions for the level model $Sensitivity = \gamma_0 + \gamma_1 \cdot SE^2$, estimated as *z* on *precision and SE* without an intercept, so that γ_0 is the bias-corrected mean and γ_1 the publication-bias term. *SE* is the standard error of the individual sensitivity estimate from the sample-level regression fit. Model 1 (raw scale) uses the original sensitivity ($z = Sensitivity/SE$; $precision = 1/SE$). Model 2 (log scale) uses log-transformed sensitivity to account for the strictly positive nature of the estimates. Within each model the two bias-corrected means (β_0 and γ_0) are directly comparable; across models they are not, and the two publication-bias terms are not comparable either, since β_1 multiplies SE whereas γ_1 multiplies SE^2. In Model 2 the precision terms are built from $SE_{log} = SE / Sensitivity$, the relative standard error, which is the first-order (delta-method) standard error of the log sensitivity. Because the dependent variable in Model 2 is the log sensitivity, β_0 and γ_0 are expressed in log units and correspond to bias-corrected geometric means of exp(0.956) = 2.60 %/ppm and exp(0.997) = 2.71 %/ppm, directly comparable with the 2.259 reported below. Reported are coefficient estimates and *t*-statistics (in parentheses), computed with cluster-robust standard errors clustered at the study level. Mean sensitivity without bias correction is the arithmetic mean on the raw scale and the geometric mean on the log scale. The mean sensitivity among the 10% most precise estimates is reported on the same basis and, in each model, ranks samples by that model's own precision measure (*1/SE* on the raw scale, *Sensitivity/SE* on the log scale), so the two columns are computed from different subsets of samples.

*** $p < 0.01$, ** $p < 0.05$, * $p < 0.10$

Table S2. Definition and summary statistics of the variables

Variable	Description	Mean	Std. Dev.	Obs.
Effect size at record level				
Norm response	Response normalized by NO_2 concentration (%/ppm)	164.77	1359.85	1,180
Stability slope	Drift rate of response during a fixed-concentration stability test	-0.63	3.11	49
Response time	Time to reach 90% of the final response after a NO_2 step (s)	93.52	159.07	348
Recovery time	Time to recover 90% toward baseline after NO_2 removal (s)	84.14	140.24	289
Effect sizes at sample level				
Breakpoint	Per-sample estimated breakpoint of the response curve (ppm)	34.45	72.76	61
Sensitivity	Per-sample estimated slope of the response curve (%/ppm)	137.72	1028.84	144
Mean of norm response	Per-sample mean of norm response before breakpoint if breakpoint exists (%/ppm)	138.78	618.91	144
Material				
MXene concentration	Per-sample percentage of MXene in the sensing material (wt.%)	76.28	27.33	143
Specific surface area	Per-sample specific surface area (m² per g)	58.54	80.96	55
Sensing material mass	Per-sample mass of the active sensing material (g)	0.02	0.06	62
In-situ oxide				
In-situ oxide	= 1 if in-situ oxide is present and MXene conc. = 100 wt.%, 0 otherwise	0.09	0.29	144
Additives				
Oxides	= 1 if oxides are present, 0 otherwise	0.21	0.41	144
Organic	= 1 if organic components are present as additives, 0 otherwise	0.19	0.40	144
Layered sulfides	= 1 if layered sulfide component is present, 0 otherwise	0.12	0.33	144
Layered w/o sulfides	= 1 if layered (non-sulfide) component is present, 0 otherwise	0.08	0.28	144
Morphology				
Multilayer	= 1 if MXene form is multilayer, 0 otherwise	0.30	0.46	144
Assembled	= 1 if MXene form is assembled, 0 otherwise	0.17	0.37	144
Nanosheet*	= 1 if MXene form is nanosheet, 0 otherwise	0.52	0.50	144
Surface state and synthesis				
OH/F termination	= 1 if OH/F surface termination is present, 0 if other termination is present	0.75	0.43	133
Etching (MeF/HCl)	= 1 if etching with MeF/HCl was used, 0 if HF was used	0.65	0.48	142
Metal ion intercalant*	= 1 if a metal ion was present as an intercalant, 0 otherwise	0.60	0.49	144
Organic intercalant	= 1 if an organic intercalant was present, 0 otherwise	0.09	0.29	144
Water intercalant	= 1 if only H_2O was present as intercalant, 0 otherwise	0.30	0.46	144
Treatment				
Sonication	= 1 if sonication was performed, 0 otherwise	0.39	0.49	132
Autoclave	= 1 if autoclave treatment was performed, 0 otherwise	0.27	0.45	143
Annealing	= 1 if annealing was performed, 0 otherwise	0.15	0.36	143
Thermal treatment	= 1 if thermal treatment was performed, 0 otherwise	0.11	0.32	144
Chemical treatment	= 1 if chemical treatment was performed, 0 otherwise	0.08	0.28	144
Device				
Drop casting	= 1 if drop casting was used for production, 0 otherwise	0.61	0.49	133
Substrate sensor	= 1 if sensor was built on a substrate, 0 otherwise	0.93	0.26	141
Inorganic substrate	= 1 if substrate is inorganic, 0 otherwise	0.64	0.48	112
Electrode				
Au electrode*	= 1 if gold electrode was used, 0 otherwise	0.71	0.46	128
Ag electrode**	= 1 if silver electrode was used, 0 otherwise	0.05	0.23	128
Pt electrode	= 1 if platinum electrode was used, 0 otherwise	0.13	0.34	128
Other electrode	= 1 if another electrode type was used, 0 otherwise	0.23	0.42	128
Experimental conditions				
Static measurement system	= 1 if the measurement system is static, 0 if it is dynamic	0.23	0.42	130
NO_2 range	Per-sample maximum minus minimum NO_2 concentration (ppm)	75.27	144.78	144
Humidity	Per-sample relative humidity (%) during the measurement	23.30	25.70	125
Publication characteristics				
Journal impact factor	Journal impact factor of the publication from Web of Science Journal Citation Reports	10.42	6.04	144

Obs. = number of observations: the number of records (*r*) for the record-level effect sizes (normalized response, stability slope, response time, recovery time) and the number of samples (*m*) for the sample-level effect sizes and all moderator variables. Std. Dev. = standard deviation. * Omitted baseline category in the meta-regression. ** It is reported separately in the bivariate subgroup analysis but merged into 'Other electrode' in the meta-regression and BMA (as stated in Section 4.5).

Table S3. List of primary studies

ID	Authors (Pub Year)	Title	Journal	Impact Factor	*m*	*r*
1	Cai et al. (2025)	Single-atom Pd confined in Ti_3C_2 nanosheet for boosted room-temperature trace NO_2 monitoring	Nano Research	9.0	1	15
2	Cao et al. (2024)	$Ti_3C_2T_x$/MoO_3 composite as an ultrasensitive and selective sensing material for a room-temperature nitrogen dioxide sensor	Applied Surface Science	6.9	5	13
3	Choi et al. (2020)	In Situ Formation of Multiple Schottky Barriers in a Ti_3C_2 MXene Film and its Application in Highly Sensitive Gas Sensors	Advanced Functional Materials	18.8	3	18
4	Das et al. (2024)	Facile Design of $Ti_3C_2T_X$ (MXene) Modified Spherical $S_rT_iO_3$ Structure toward Highly Sensitive Room Temperature Detection of NO_2	ACS Applied Electronic Materials	4.7	1	15
5	Devara et al. (2024)	Enhanced sensitivity of zero-bias-operated MXene chemiresistive sensor via lignin hybridization	EcoMat	12.6	2	8
6	Dhariwal et al. (2024)	Engineering an Ultrafast Ambient NO_2 Gas Sensor Using Cotton-Modified $LaFeO_3$/MXene Composites	ACS Sensors	9.1	1	36
7	Ding et al. (2025)	MOF-Derived NiO–SnO_2/$Ti_3C_2T_x$ Composite Materials for Detection of ppb-Level NO_2 at Room Temperature	ACS Applied Nano Materials	5.6	2	7
8	Fan et al. (2022)	Fast and recoverable NO_2 detection achieved by assembling ZnO on $Ti_3C_2T_x$ MXene nanosheets under UV illumination at room temperature	Nanoscale	6.7	2	12
9	Fan et al. (2022)	Synthesis and characterization of an ultra-thin BiOCl/MXene heterostructure for the detection of NO_2 at room temperature with enhanced moisture resistance	Journal of Materials Chemistry A	11.9	4	86
10	Feng et al. (2023)	DFT investigation and Polypyrrole sensitization mechanism of a selective NO_2 sensor for room temperature application based on MXene@PPy heterojunction	Surfaces and Interfaces	6.0	4	16
11	Gasso and Mahajan (2022)	Development of Highly Sensitive and Humidity Independent Room Temeprature NO_2 Gas Sensor Using Two Dimensional $Ti_3C_2T_x$ Nanosheets and One Dimensional WO_3 Nanorods Nanocomposite	ACS Sensors	8.9	1	1
12	Gasso and Mahajan (2022)	MXene decorated tungsten trioxide nanocomposite-based sensor capable of detecting NO_2 gas down to ppb-level at room temperature	Materials Science in Semiconductor Processing	4.1	5	13
13	Gasso and Mahajan (2023)	Self-Powered Wearable Gas Sensors Based on L-Ascorbate-Treated MXene Nanosheets and SnO_2 Nanofibers	ACS Applied Nano Materials	5.5	3	18
14	Gasso et al. (2022)	MXene modulated SnO_2 gas sensor for ultra-responsive room-temperature detection of NO_2	Sensors and Actuators B: Chemical	8.4	5	16
15	Guo et al. (2023)	A room-temperature NO_2 sensor based on $Ti_3C_2T_X$ MXene modified with sphere-like CuO	Sensors and Actuators B: Chemical	7.7	4	28
16	Hermawan et al. (2025)	Achieving high-performance NO_2 sensors via vertically-aligned Ti_3C_2T nanoarchitectures	Sensors and Actuators B: Chemical	7.7	2	12
17	Hilal et al. (2024)	Tailoring MXene Thickness and Functionalization for Enhanced Room-Temperature Trace NO_2 Sensing	Nano-Micro Letters	36.3	3	60

18	Hu et al. (2024)	Plasmonic photothermal driven MXene-based gas sensor for highly sensitive NO_2 detection at room temperature	Sensors and Actuators B: Chemical	7.6	5	12
19	Jian et al. (2021)	The prior rules of designing $Ti_3C_2T_x$ MXene-based gas sensors	Frontiers of Chemical Science and Engineering	4.8	4	12
20	Kim et al. (2023)	Three-Dimensional MoS_2/MXene Heterostructure Aerogel for Chemical Gas Sensors with Superior Sensitivity and Stability	ACS Nano	16.1	5	35
21	Kim et al. (2025)	Surface functionalized MXene ink-enabled washable smart e-textiles with exceptional gas sensing properties	Materials Today	22.0	2	10
22	Le et al. (2022)	Flexible and high-sensitivity sensor based on Ti_3C_2-MoS_2 MXene composite for the detection of toxic gases	Chemosphere	8.8	6	18
23	Li et al. (2024)	$Ti_3C_2T_x$ Nanosheet/Bi_2S_3 Nanobelt Composites for NO_2 Gas Sensing	ACS Applied Nano Materials	5.3	4	14
24	Li et al. (2025)	Epitaxy Growth of Hofmann MOF on Monolayer MXene for ppb-Level Room-Temperature NO_x Sensing	Advanced Functional Materials	19.0	1	7
25	Liu et al. (2022)	Enhanced room-temperature NO_2 sensing performance of SnO_2/Ti_3C_2 composite with double heterojunctions by controlling co-exposed {221} and {110} facets of SnO_2	Sensors and Actuators B: Chemical	8.4	2	15
26	Liu et al. (2023)	ZnO/Ti_3C_2 composite with oxygen vacancies and Schottky barrier for effective detection of ppb-level NO_2 at room temperature	Applied Surface Science	6.9	3	21
27	Liu et al. (2024)	2D/2D {001}TiO_2-/Ti_3C_2T composite with ultra-high sensing performance for NO_2 at room temperature	Sensors and Actuators B: Chemical	7.6	4	28
28	Liu et al. (2021)	Enhanced NO_2 gas-sensing performance of 2D Ti_3C_2/TiO_2 nanocomposites by in-situ formation of Schottky barrier	Applied Surface Science	7.4	6	16
29	Liu et al. (2024)	Flexible resistive NO_2 gas sensor of SnO_2@$Ti_3C_2T_x$ MXene for room temperature application	Ceramics International	5.6	4	24
30	Liu et al. (2024)	Synergistic Etching and Hydrogen Bonding-Induced Self-Assembly of MXene/MOF Hybrid Aerogel for Flexible Room-Temperature Gas Sensing	ACS Sensors	9.1	6	56
31	Liu et al. (2022)	A 2D/2D/2D $Ti_3C_2T_x$@TiO_2@MoS_2 heterostructure as an ultrafast and high-sensitivity NO_2 gas sensor at room-temperature	Journal of Materials Chemistry A	11.9	2	23
32	Peng et al. (2024)	Gas-sensitive performance of metal-organic framework-derived CuO NPs/$Ti_3C_2T_X$ MXene heterostructures for efficient NO_2 detection at room temperature	Journal of Alloys and Compounds	6.3	3	37
33	Quan et al. (2023)	Fully Flexible MXene-based Gas Sensor on Paper for Highly Sensitive Room-Temperature Nitrogen Dioxide Detection	ACS Sensors	9.1	4	52
34	Quan et al. (2024)	Quantum Confinement and End-Sealing Effects for Highly Sensitive and Stable Nitrogen Dioxide Detection: Homogeneous Integration of $Ti_3C_2T_x$-Based Flexible Gas Sensors	ACS Sensors	9.1	7	40
35	Sardana et al. (2023)	WS_2 nanosheets decorated multi-layered MXene based chemiresistive sensor for efficient detection and discrimination of NH_3 and NO_2	Sensors and Actuators B: Chemical	7.7	3	15
36	Ta et al. (2022)	Enhanced Gas Sensing Performance of ZnO/$Ti_3C_2T_x$ MXene Nanocomposite	Micromachines	3.4	3	10

37	Ta et al. (2024)	In situ growth of TiO_2 on $Ti_3C_2T_x$ MXene for improved gas-sensing performances	Ceramics International	5.6	4	11
38	Ta et al. (2021)	Highly surface-active Si-doped $TiO_2/Ti_3C_2T_x$ heterostructure for gas sensing and photodegradation of toxic matters	Chemical Engineering Journal	16.7	3	6
39	Tang et al. (2024)	Molten salt-modified $Ti_3C_2T_x$ MXene with tunable oxygen-functionalized surfaces for effective detection of NO_2 at room temperature	Ceramics International	5.6	2	15
40	Tang et al. (2025)	Room Temperature Resistive NO_2 Sensor Based on SnS_2 Quantum Dots/$Ti_3C_2T_x$ MXene Heterostructures	ACS Applied Nano Materials	5.6	3	18
41	Thanh et al. (2022)	Improved NO_2 gas sensing performance of 2D $MoS_2/Ti_3C_2T_x$ MXene nanocomposite	Applied Surface Science	6.7	1	12
42	Tran et al. (2021)	rGO/$Ti_3C_2T_x$ heterostructures for the efficient, room-temperature detection of multiple toxic gases	Materials Chemistry and Physics	4.8	6	13
43	Tseng et al. (2023)	Synthesis of $Ti_3C_2T_x$/ZnO composites decorated with PEDOT:PSS for NO_2 gas sensors	The International Journal of Advanced Manufacturing Technology	3.1	2	8
44	Wang et al. (2021)	Multifunctional poly(vinyl alcohol)/Ag nanofibers-based triboelectric nanogenerator for self-powered MXene/tungsten oxide nanohybrid NO_2 gas sensor	Nano Energy	19.1	6	21
45	Wang et al. (2023)	d-Band Center Optimization of $Ti_3C_2T_x$ MXene Nanosheets for Ultrahigh NO_2 Gas Sensitivity at Room Temperature	ACS Applied Materials & Interfaces	8.2	2	25
46	Wang et al. (2023)	Atomic Plasma Grafting: Precise Control of Functional Groups on $Ti_3C_2T_x$ MXene for Room Temperature Gas Sensors	ACS Applied Materials & Interfaces	8.2	4	27
47	Wu et al. (2022)	Fabrication of SnO_2-TiO_2-$Ti_3C_2T_x$ hybrids with multiple-type heterojunctions for enhanced gas sensing performance at room temperature	Applied Surface Science	6.7	3	18
48	Xia et al. (2021)	MXene/WS_2 hybrids for visible-light-activated NO_2 sensing at room temperature	Chemical Communications	6.1	2	2
49	Xu et al. (2022)	Black phosphorus quantum dots modified monolayer $Ti_3C_2T_x$ nanosheet for field-effect transistor gas sensor	Sensors and Actuators B: Chemical	8.4	2	30
50	Yamunasree et al. (2024)	Ultrasensitive and highly selective NO_2 gas sensing of porous MXene nanoribbon assemblies	Carbon	11.6	2	6
51	Yan et al. (2022)	2H-$MoS_2/Ti_3C_2T_x$ MXene composites for enhanced NO_2 gas sensing properties at room temperature	Sensors and Actuators Reports	5.9	2	20
52	Yang et al. (2022)	Self-Assembly 3D Porous Crumpled MXene Spheres as Efficient Gas and Pressure Sensing Material for Transient All-MXene Sensors	Nano-Micro Letters	26.6	2	13
53	Yang et al. (2021)	Flexible resistive NO_2 gas sensor of three-dimensional crumpled MXene $Ti_3C_2T_x$/ZnO spheres for room temperature application	Sensors and Actuators B: Chemical	9.2	1	5
54	Yang et al. (2022)	The Introduction of Defects in $Ti_3C_2T_x$ and $Ti_3C_2T_x$‐Assisted Reduction of Graphene Oxide for Highly Selective Detection of ppb‐Level NO_2	Advanced Functional Materials	19.0	8	48
55	Zhang et al. (2022)	High-Performance Ppb Level NO_2 Gas Sensor Based on Colloidal SnO_2 Quantum Wires/Ti_3C_2Tx MXene Composite	Nanomaterials	5.3	4	4
56	Zhang et al. (2023)	Laser Processing of Crumpled Porous Graphene/MXene Nanocomposites for a Standalone Gas Sensing System	Nano Letters	9.1	1	8

57	Zhang et al. (2023)	Au nanoparticles decorated flower-like SnS_2/Ti_3C_2 MXene microcomposite for high performance NO_2 sensing at room temperature	Applied Surface Science	6.9	1	11
58	Zhang et al. (2024)	Enhanced Room-Temperature NO_2 Sensing through Deep Functional Group Hybridization in Nitrogen-Doped Monolayer $Ti_3C_2T_x$	Sensors and Actuators B: Chemical	7.6	4	22
59	Zhang et al. (2024)	Key to unlocking NO_2 sensing performance of monolayer $Ti_3C_2T_x$: Regulating OH/F functional groups	Chemical Engineering Journal	13.2	2	16
60	Zhao et al. (2021)	Enhanced Blocking Effect: A New Strategy to Improve the NO_2 Sensing Performance of $Ti_3C_2T_x$ by γ-Poly(l-glutamic acid) Modification	ACS Sensors	9.6	4	51
61	Zhou et al. (2023)	Passivation of MXene via atomic layer deposition of SnO_2 to achieve improved NO_2 sensing	Applied Physics Letters	3.6	2	14

This table lists the 61 studies included in the systematic search, providing 195 unique samples and 1243 records. A sample (*m*) is a specific sensing material with unique properties; each sample provides one or more records (*r*), where a record is a single measurement of sensor performance at a specific combination of operating conditions. Journal impact factors are from the Web of Science Journal Citation Reports (as retrieved). Bibliographic details of the studies are given in the supplement's reference list.

Table S4. List of studies identified for MXene systems other than $Ti_3C_2T_x$

Authors (Pub Year)	MXene
Chen et al. (2022)[1]	$TiC_{0.5}N_{0.5}$
Choi et al. (2022)[2]	Mo_2CT_x
Kumar & Pal (2022)[3]	Nb_2CT_x
Li et al. (2024)[4]	$Mo_2TiC_2T_x$
Rathi et al. (2022)[5]	Nb_2CT_x
Simonenko et al. (2023)[6]	Ti_2CT_x
Simonenko et al. (2024)[7]	Ti_2CT_x/TiO_2
Simonenko et al. (2024)[8]	$Ti_{0.2}V_{1.8}CT_x$
Simonenko et al. (2024)[9]	Ti_2CT_x
Wang et al. (2024)[10]	Mo_2CT_x
Zhang et al. (2021)[11]	V_2CT_x
Zhang et al. (2024)[12]	V_2CT_x
Zhao et al. (2022)[13]	$Mo_2TiC_2T_x$

This table lists the studies excluded at the full-text stage of the systematic search under the material specification criterion because the sensing material was based on an MXene phase other than $Ti_3C_2T_x$. This heterogeneity, together with the small number of NO_2-specific quantitative reports per chemistry, is why non-$Ti_3C_2T_x$ MXenes could not be included in the meta-analysis.

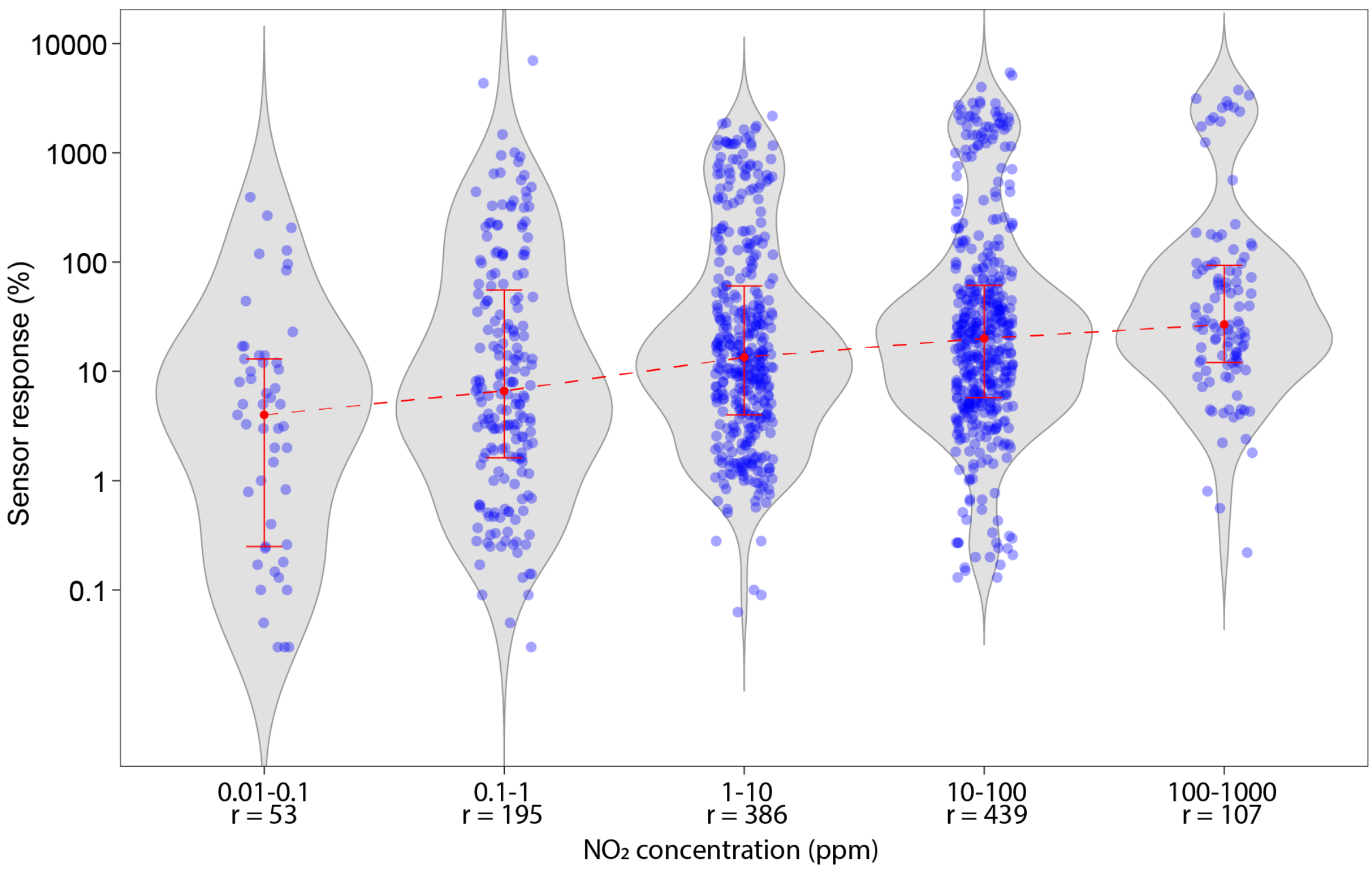

Figure S1. Distribution of sensor responses per NO_2 concentration bin: Violin plots show the density of sensor responses (record level; r = 1180 total) within five decadal NO_2 concentration bins (0.01–0.1, 0.1–1, 1–10, 10–100, and 100–1000 ppm; per-bin r shown on the axis). Blue points are individual measurements; red points and bars denote the per-bin median and interquartile range, and the dashed line connects the medians across bins.

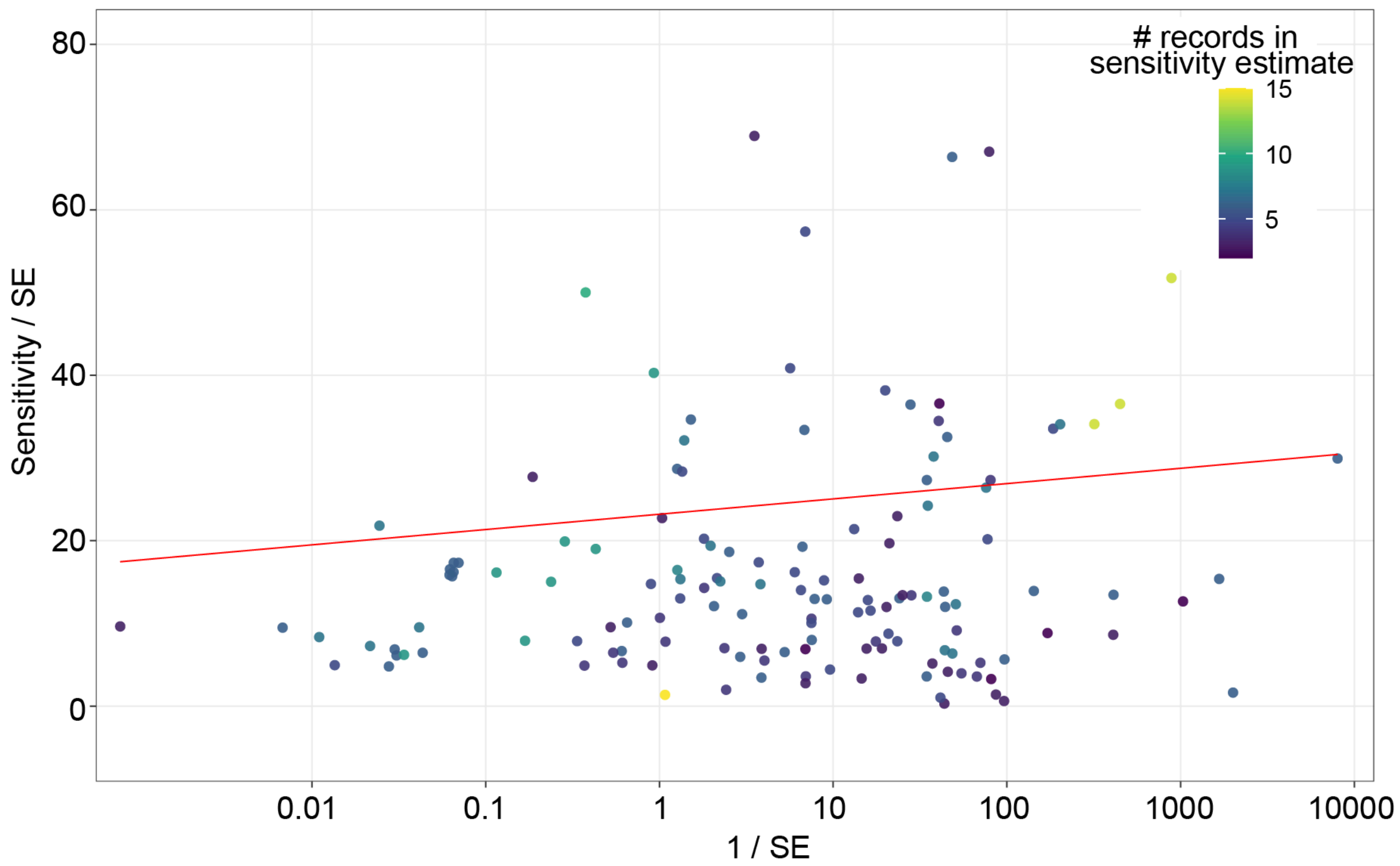

Figure S2. FAT–PET meta-regression scatter plot for sensor sensitivity: Standardized sensitivity (*z* = *sensitivity/SE*) versus precision (*1/SE*), where SE is the standard error of the individual sample-level sensitivity estimate. The x-axis is log-scaled, and points are colored by the number of records contributing to each estimate. The red line is the FAT–PET regression of *z* on precision. In this parametrization, the coefficient on precision (β_0) is the precision-effect (PET) term, estimating the bias-corrected mean effect, while the intercept (β_1) is the funnel-asymmetry (FAT) term, reflecting small-study/publication-bias effects.

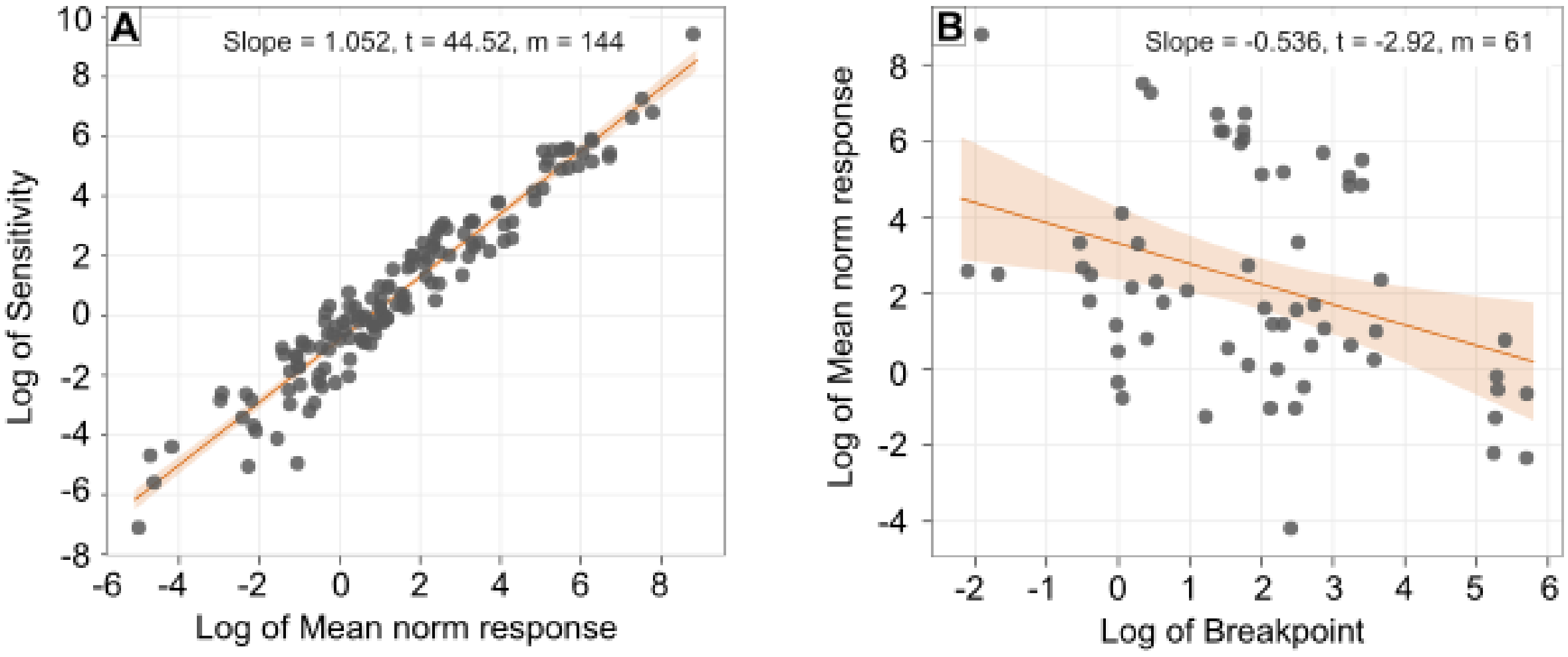

Figure S3. Relationship among effect sizes: (A) Natural logarithm of sensitivity vs. natural logarithm of mean normalized response. (B) Natural logarithm of mean normalized response vs. natural logarithm of breakpoint. Points are individual samples; the solid line is an OLS fit with a 95% confidence band, and the inset reports the fitted slope, *t*-statistic, and sample size.

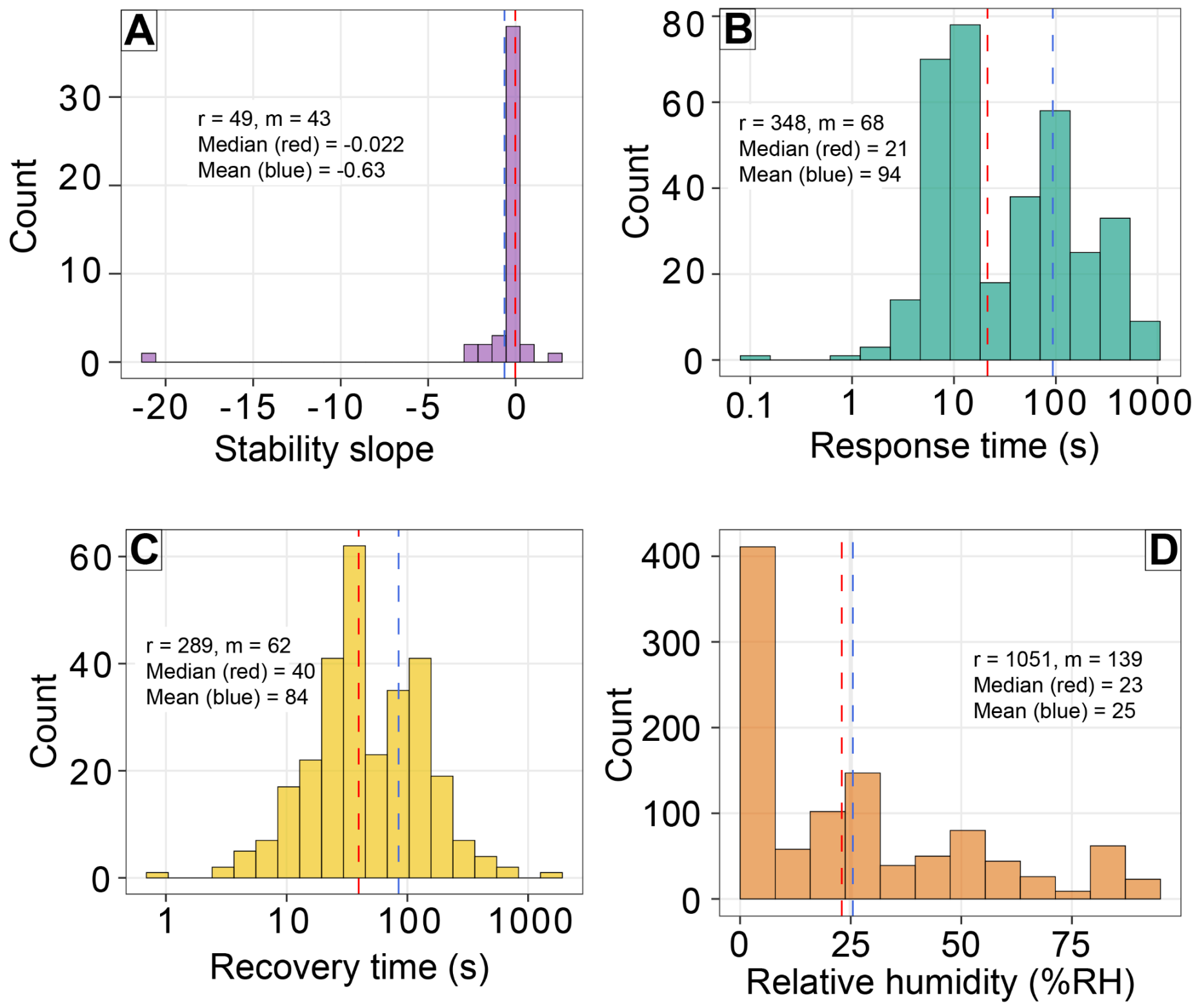

Figure S4. Distributions of additional record-level parameters: (A) stability slope, (B) response time, (C) recovery time, and (D) relative humidity. All panels are at the record level; each panel subtitle reports the record (*r*) and sample (*m*) counts together with the median and mean. Red dashed lines mark medians; blue dashed lines mark arithmetic means. (B) and (C) use a logarithmic x-axis; (A) and (D) are linear.

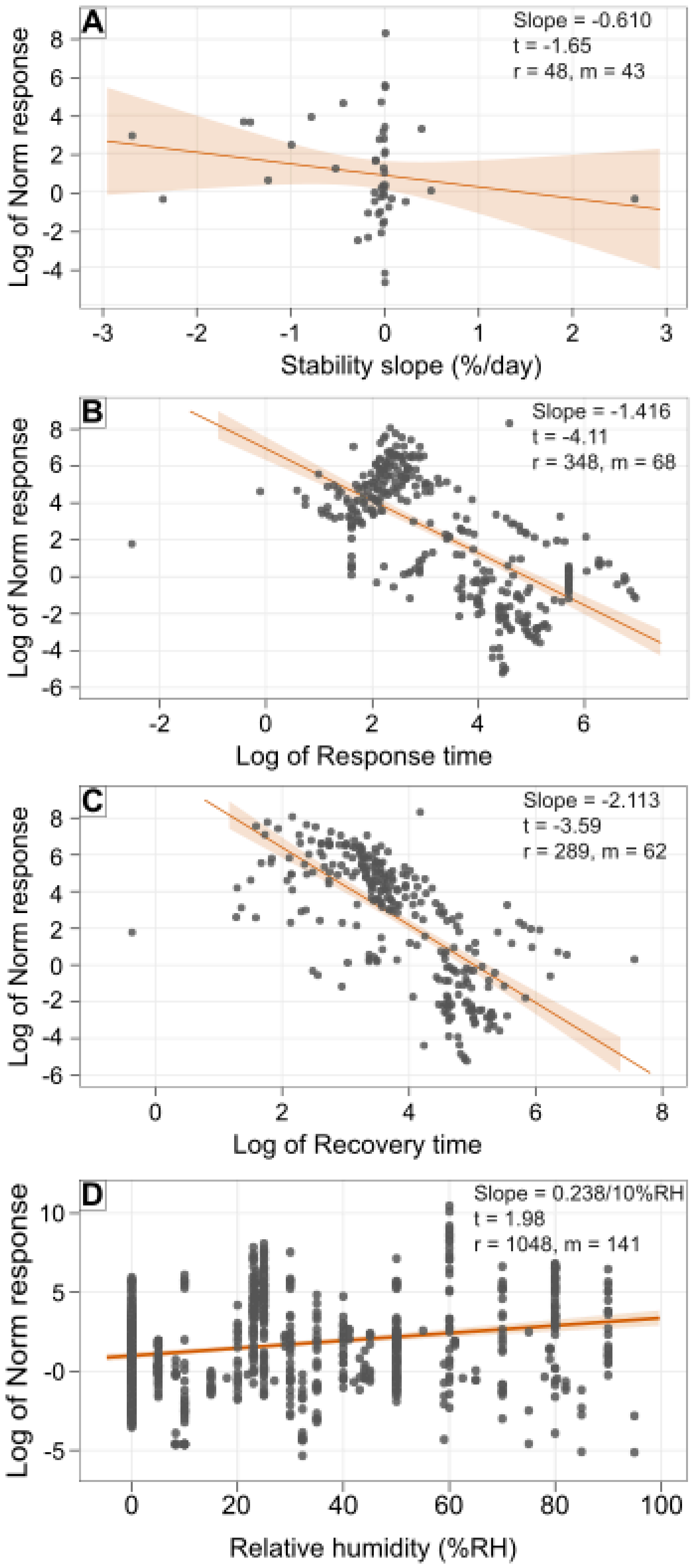

Figure S5. Correlations of additional parameters with the normalized response: Log of normalized response versus (A) stability slope, (B) log response time, (C) log recovery time, and (D) relative humidity. Points are individual records; the solid line is an OLS fit with a 95% confidence band, and the inset reports the fitted slope, *t*-statistic, and the record (*r*) and sample (*m*) counts. Standard errors are clustered at the study level. In (A), one record with a stability slope of −21.4 is excluded. In (D), the slope is expressed per 10% RH.

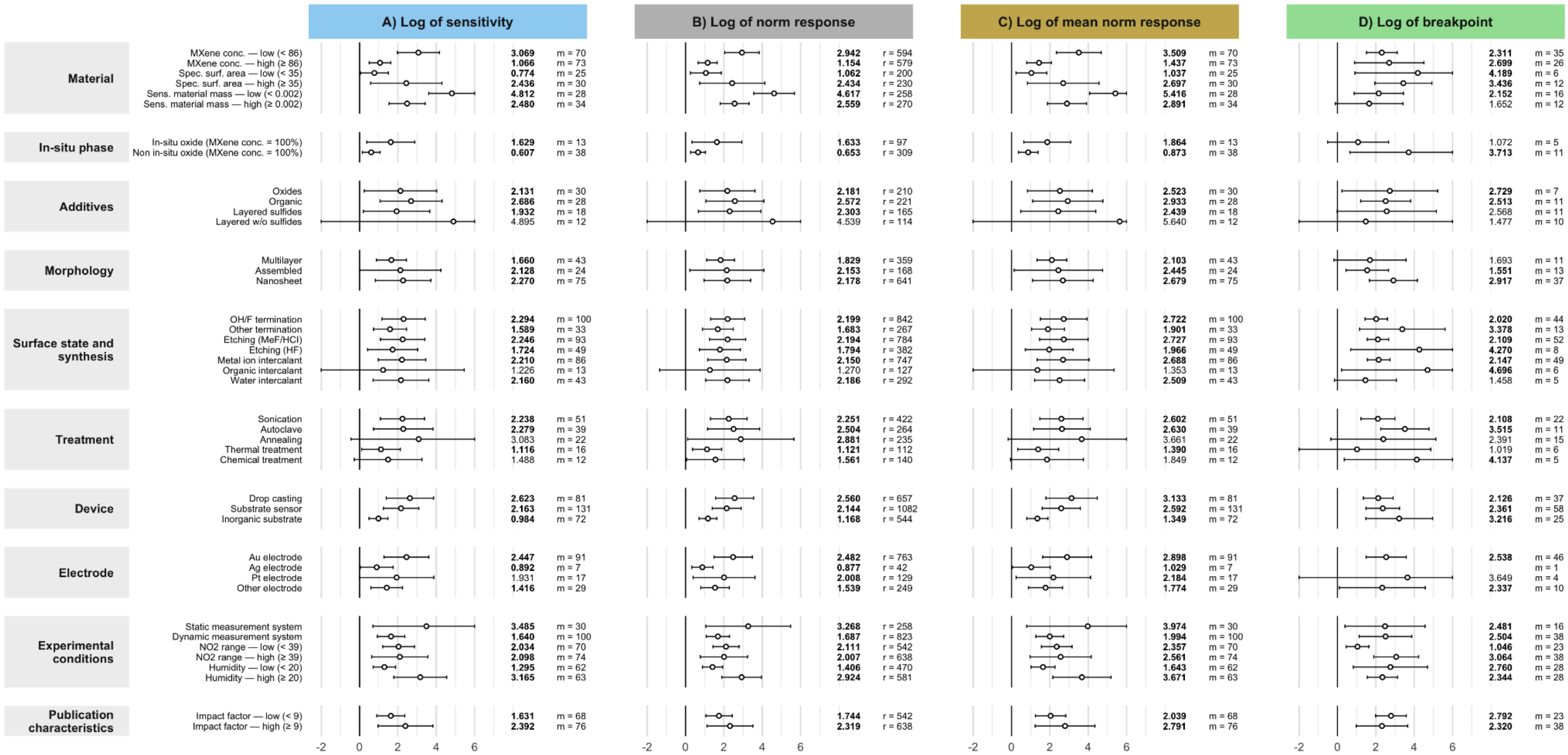

Figure S6. Subgroup analyses across outcome variables: The figure illustrates the estimates of the subgroup means for the natural logarithm of (A) sensitivity, (B) normalized response, (C) mean normalized response, and (D) breakpoint. Normalized response is analyzed at the record level (r); sensitivity, mean normalized response, and breakpoint at the sample level (m). The mean values were calculated using weighted least squares with weights proportional to the number of data points per sample (giving greater influence to observations from larger samples). 95% confidence intervals are truncated to −2 to 6 and calculated with cluster-robust standard errors that account for within-study correlations. Subgroups with fewer than three observations were excluded. Estimated mean values are shown next to the graphs. Bold values are statistically significant. Variable descriptions can be found in Table S2.

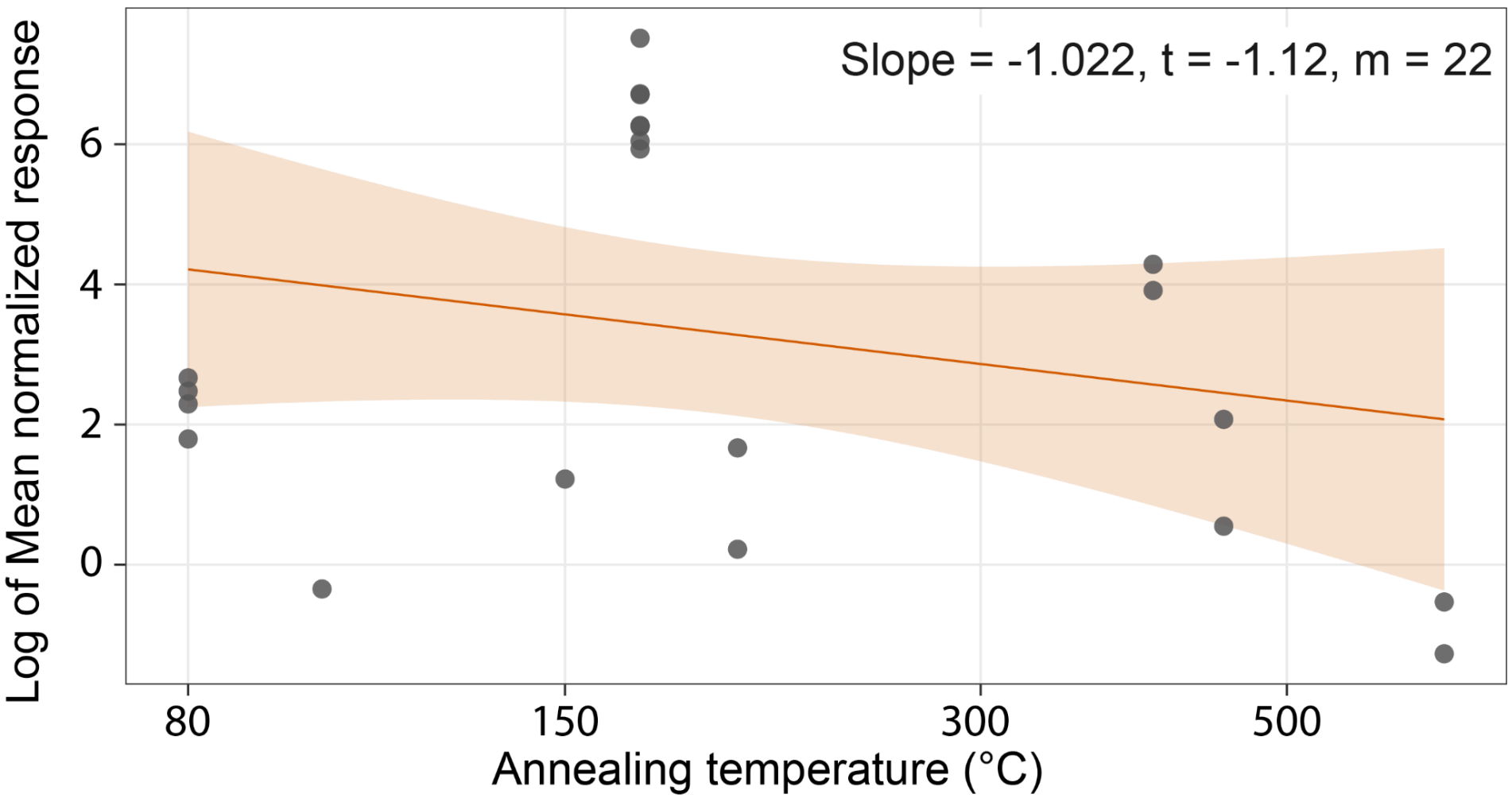

Figure S7. Relationship between annealing temperature and the log of the mean normalized response: Each point represents a single sample; annealing temperature is shown on a logarithmic scale. The solid line is an OLS fit with a 95% confidence band.

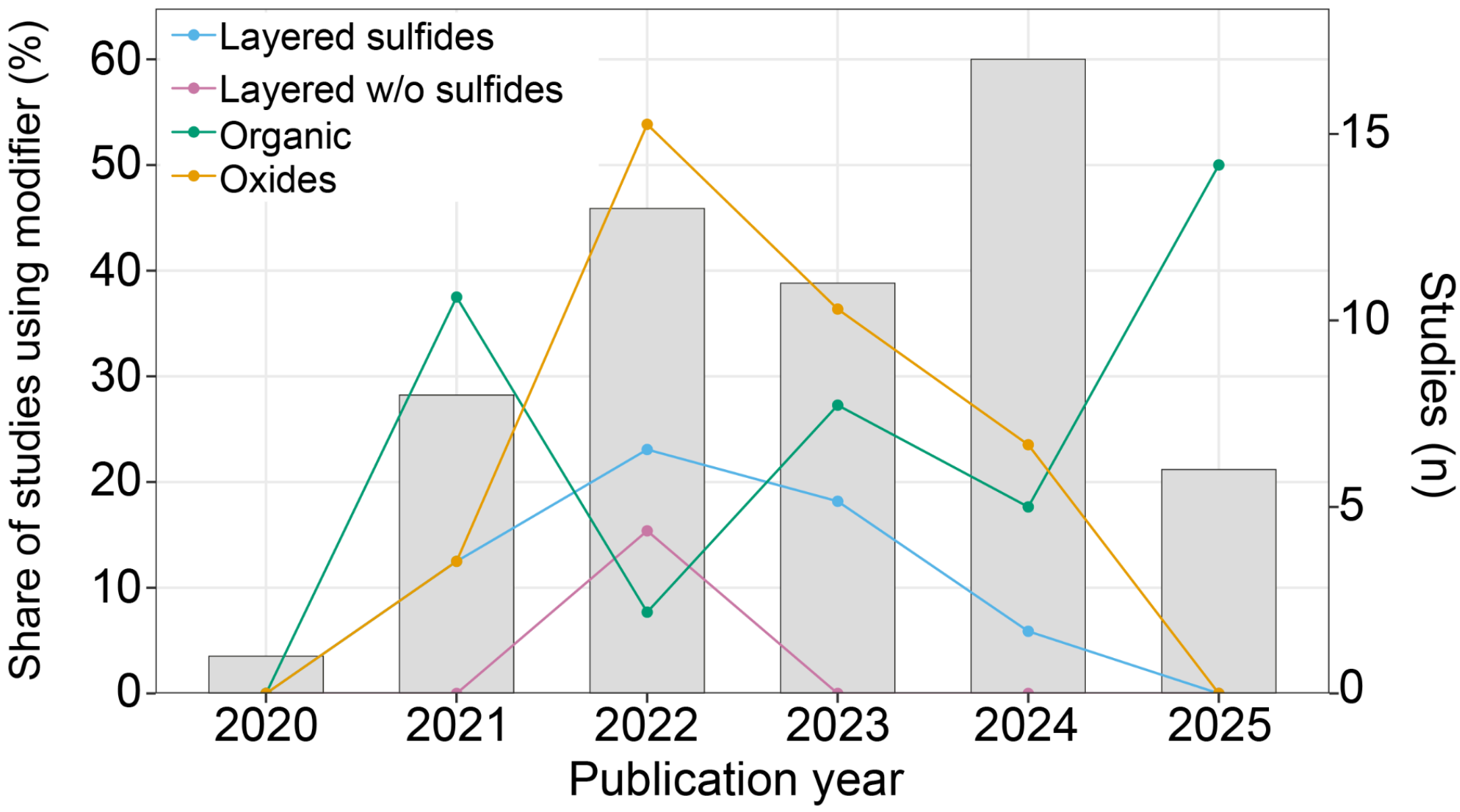

Figure S8. Distribution of modifiers over time: Annual share (%) of all published MXene sensor studies that used each modifier group. Light gray bars show the total number of studies per year (secondary axis).

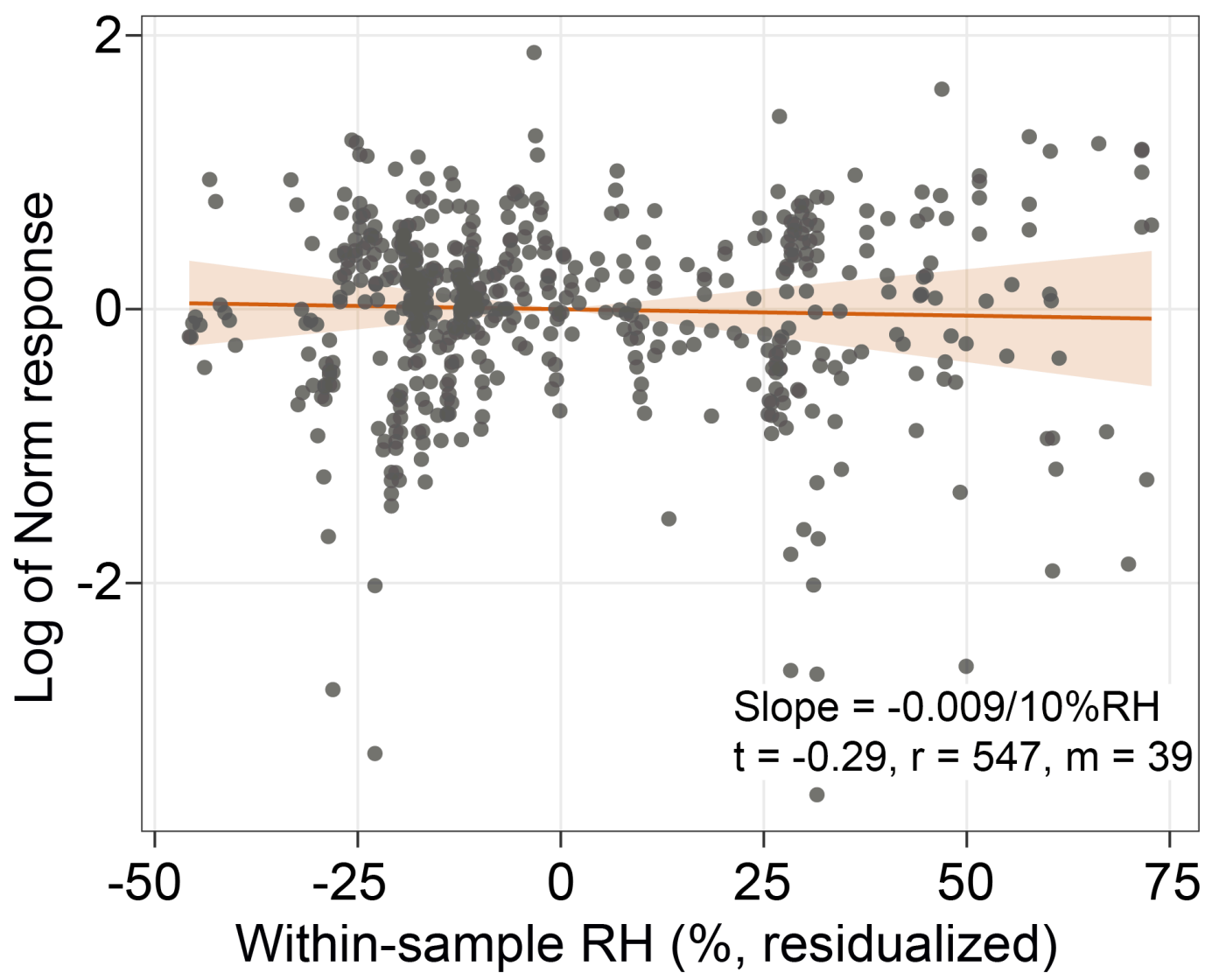

Figure S9. Within-sensor humidity effect: Plot of log-normalized response against relative humidity, each demeaned within sample and residualized on ln NO_2 concentration, for records from samples measured at more than one humidity level. The red line is the sample fixed-effects slope with its 95% confidence interval (cluster-robust standard errors).

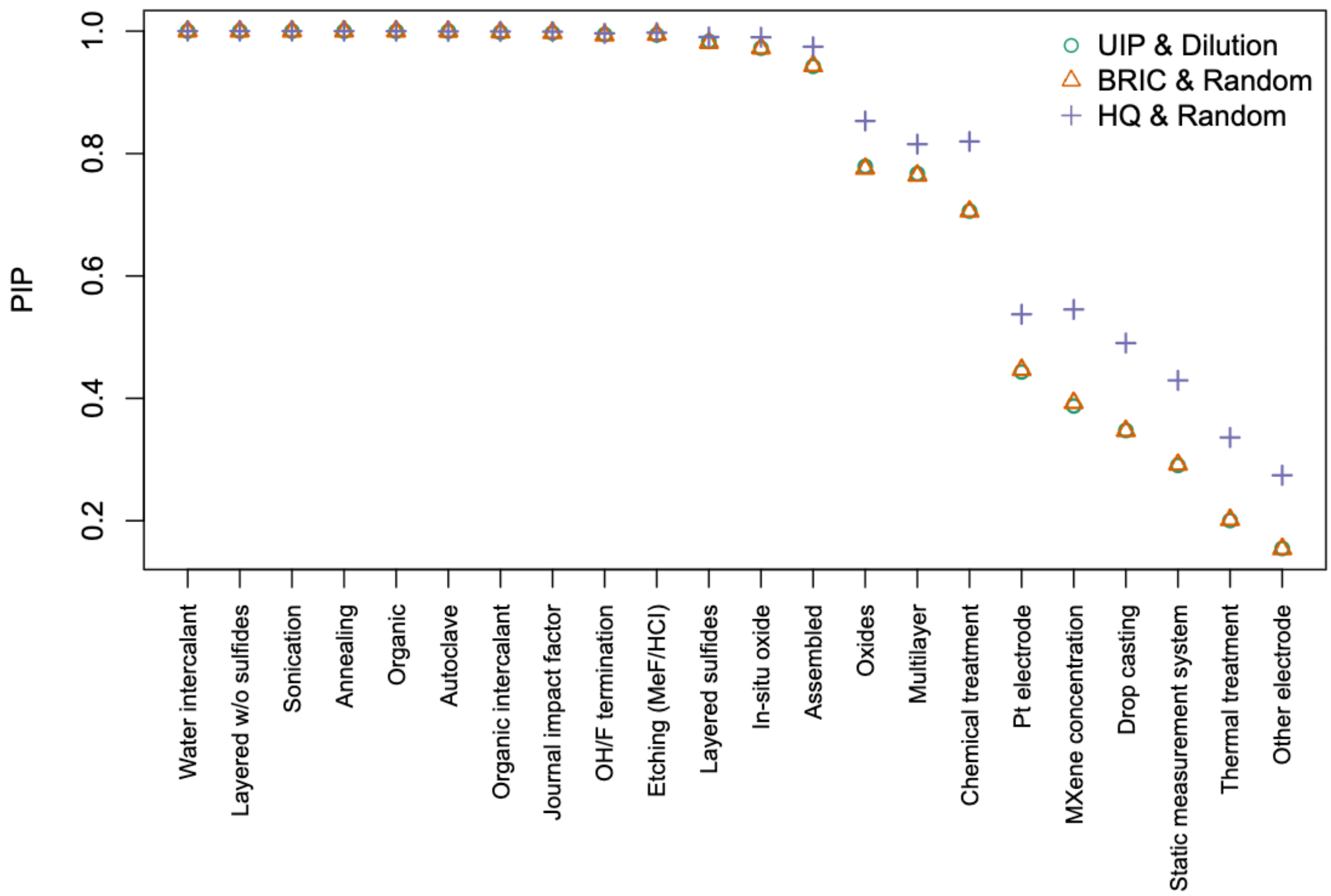

Figure S10. Robustness test of Bayesian Model Averaging with alternative priors: Posterior inclusion probabilities (PIPs) for all moderator variables under three alternative BMA prior choices. UIP and Dilution = Unit-information g-prior for parameters with a dilution model prior on the model space to penalize redundant regressors. BRIC and Random = Benchmark g-prior for parameters with the beta-binomial model prior. HQ and Random = Asymptotically mimics the Hannan-Quinn criterion. Consistent PIPs across priors indicate robust variable selection.

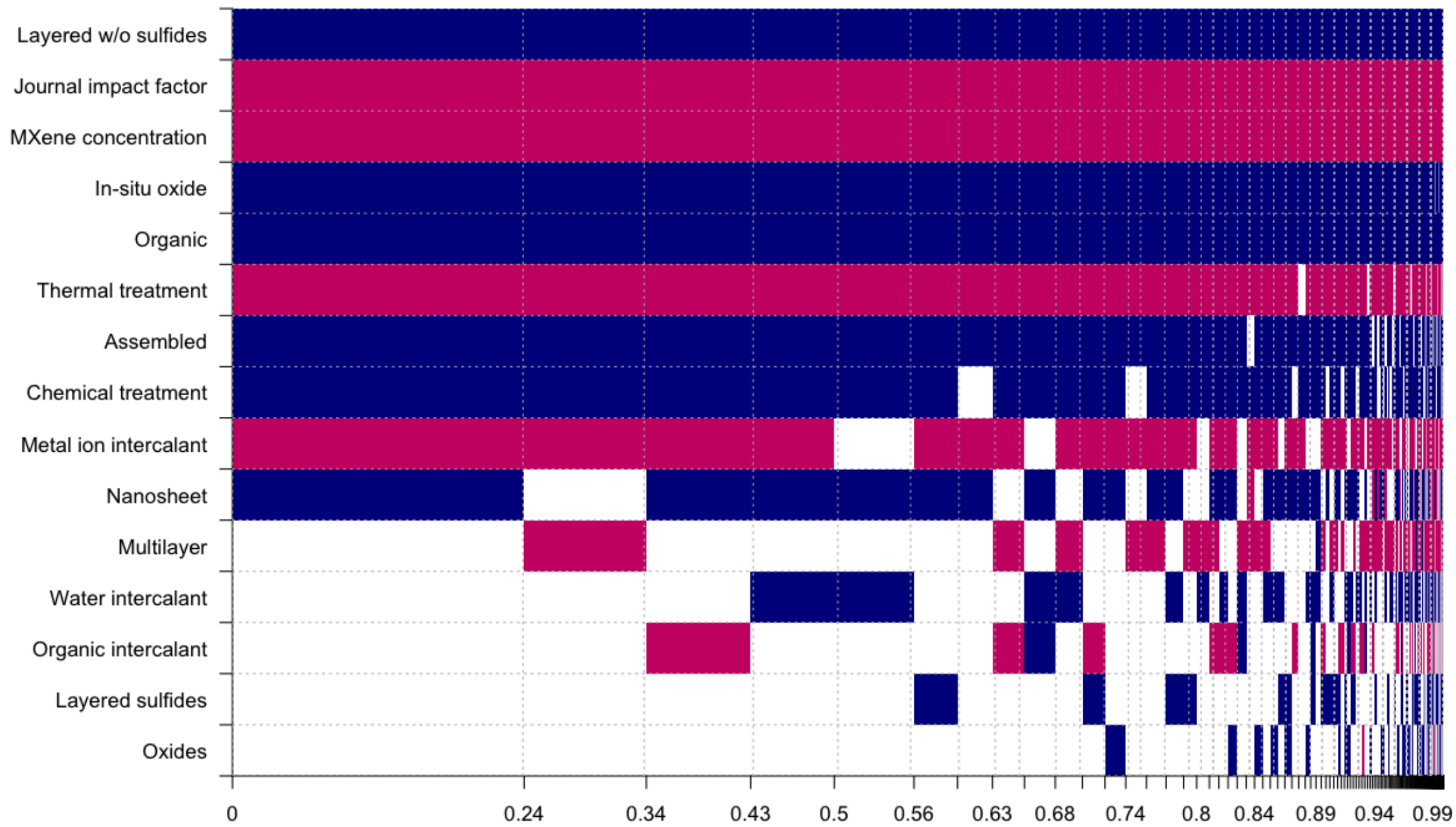

Figure S11. Bayesian Model Averaging heatmap for moderators without missing values: The figure shows the results of the Bayesian model averaging, limiting the number of moderator variables to complete-case moderator variables (without missing values). The number of observations is r = 1170 from n = 55 studies. Variable definitions can be found in Table S2.

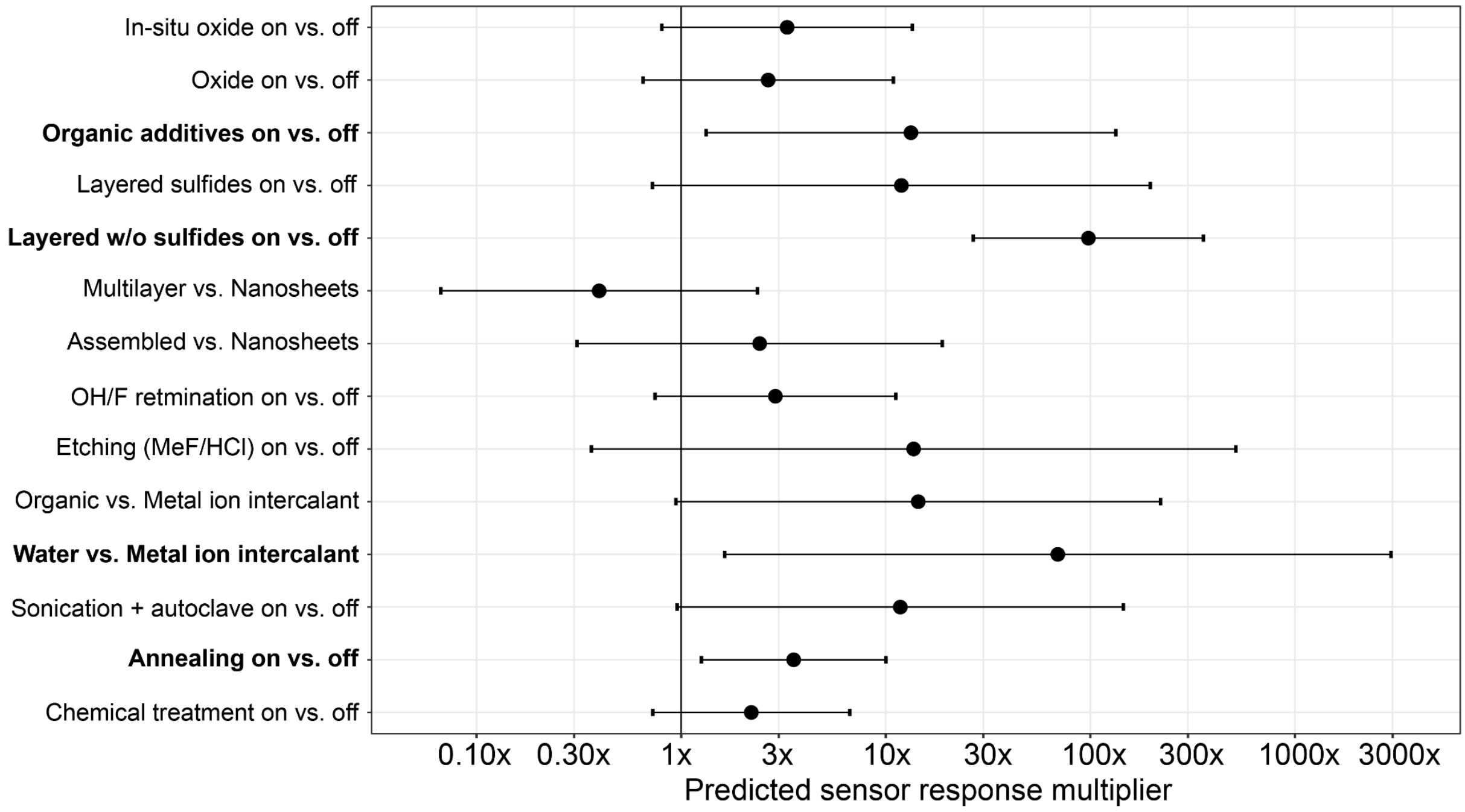

Figure S12. Predicted normalized responses assuming best-practice conditions using OLS results: Points show model-based contrasts in predicted sensor response (normalized by NO_2 concentration) from the OLS meta-regression in Table 1 (Main manuscript). Each row compares the predicted sensor response from two models; the multiplier is the ratio of the predicted response for the model shown to the left of "vs." divided by the response to the right. This ratio is calculated by taking the exponent of the difference between the two log-transformed predicted responses. A multiplier > 1x indicates a higher predicted response for the left-hand configuration, while 1x denotes no difference. Horizontal lines are 95% confidence intervals, using cluster-robust (study-level) standard errors. Predictions hold all other moderators at the 'best-practice' values. Variables in bold are statistically significant at the 5% level.

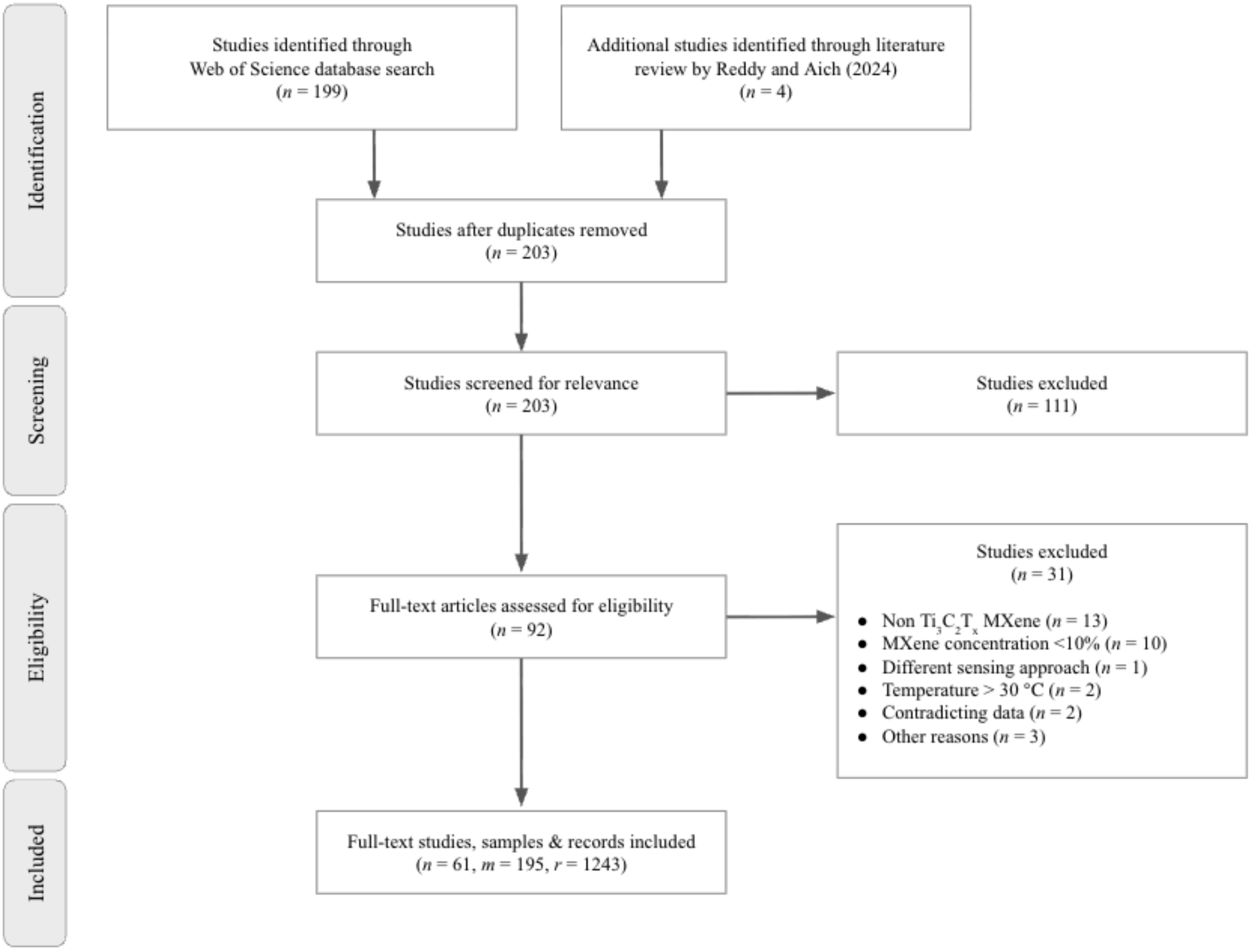

Figure S13. PRISMA flow chart of the literature search process: The diagram follows the Preferred Reporting Items for Systematic Reviews and Meta-Analyses (PRISMA) guidelines and summarizes how studies were identified, screened, and assessed for eligibility to arrive at the final sample used in the meta-analysis.

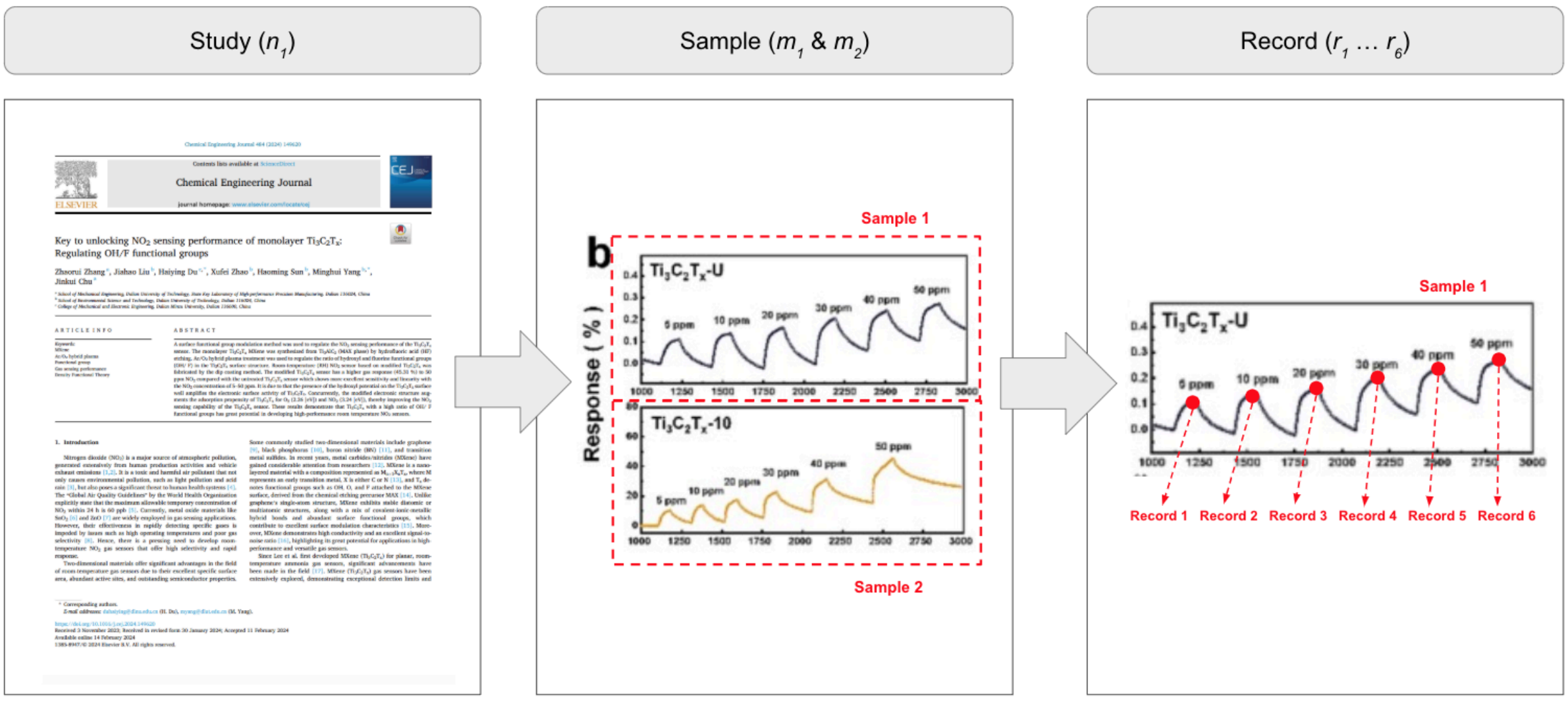

Figure S14. Hierarchical structure of our metadata set: The figure illustrates the differentiation between studies, samples, and records for the study by Zhang et al. (2024, ID 59 in Table S3). Each study (*n*) may include one or multiple samples (*m*), and each sample may contain one or multiple sensor response records (*r*).

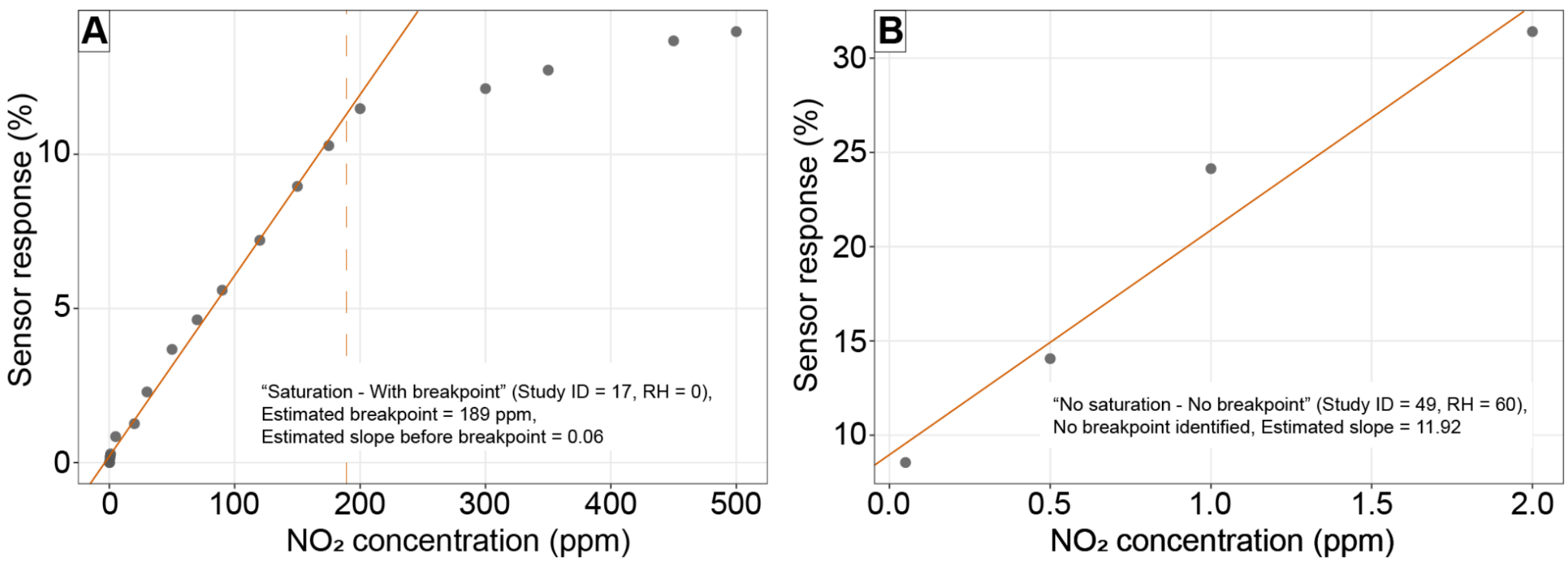

Figure S15. Breakpoint identification and sensitivity estimation: The figure shows two typical response curve types found in samples collected from the primary studies. Panel (A) illustrates a saturation curve with an identifiable breakpoint, where the response initially increases linearly with NO_2 concentration but then plateaus or shows a diminishing rate of increase after a certain concentration (the estimated breakpoint); the example is a sample from Study 17 (Hilal et al., 2024) measured at 0% RH. Panel (B) is the case where a linear relationship without saturation is observed, and therefore, no breakpoint is identified; the example is a sample from Study 49 (Xu et al., 2022) measured at 60% RH. The breakpoint is statistically identified using a segmented regression approach, and the sensitivity (curve slope) is estimated for the linear segment prior to the breakpoint using a linear regression model.

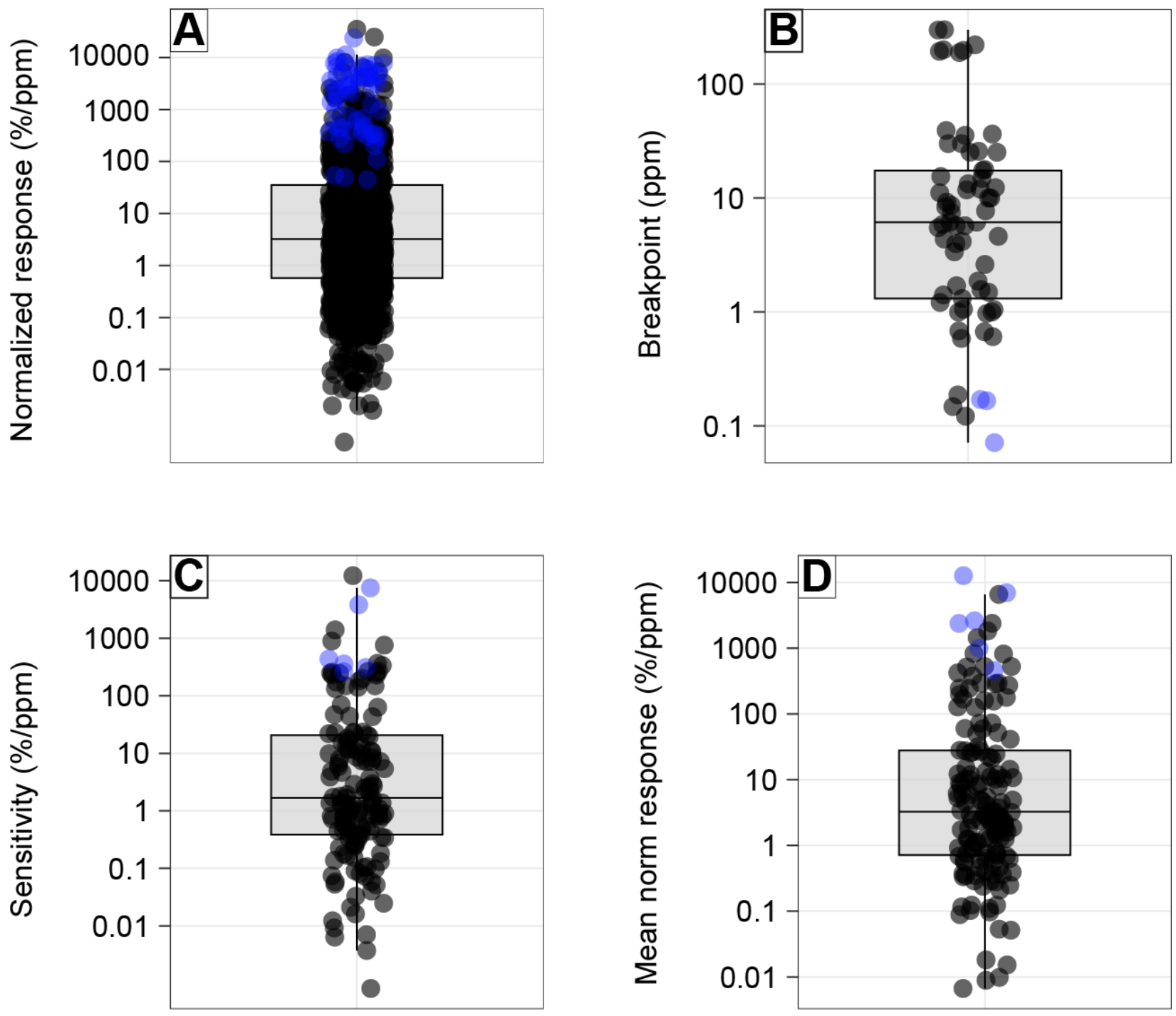

Figure S16. Outlier analysis for key sensor metrics: For normalized response, all sensor responses (record level) are shown. For breakpoint, sensitivity, and mean normalized response, only one value per sample is shown, following the effect size estimation described in Section 4.3. Data points from the five excluded studies are marked in blue: Study 11 (Gasso and Mahajan, 2022), Study 12 (Gasso and Mahajan, 2022), Study 13 (Gasso and Mahajan, 2023), Study 14 (Gasso et al., 2022), and Study 35 (Sardana et al., 2023).

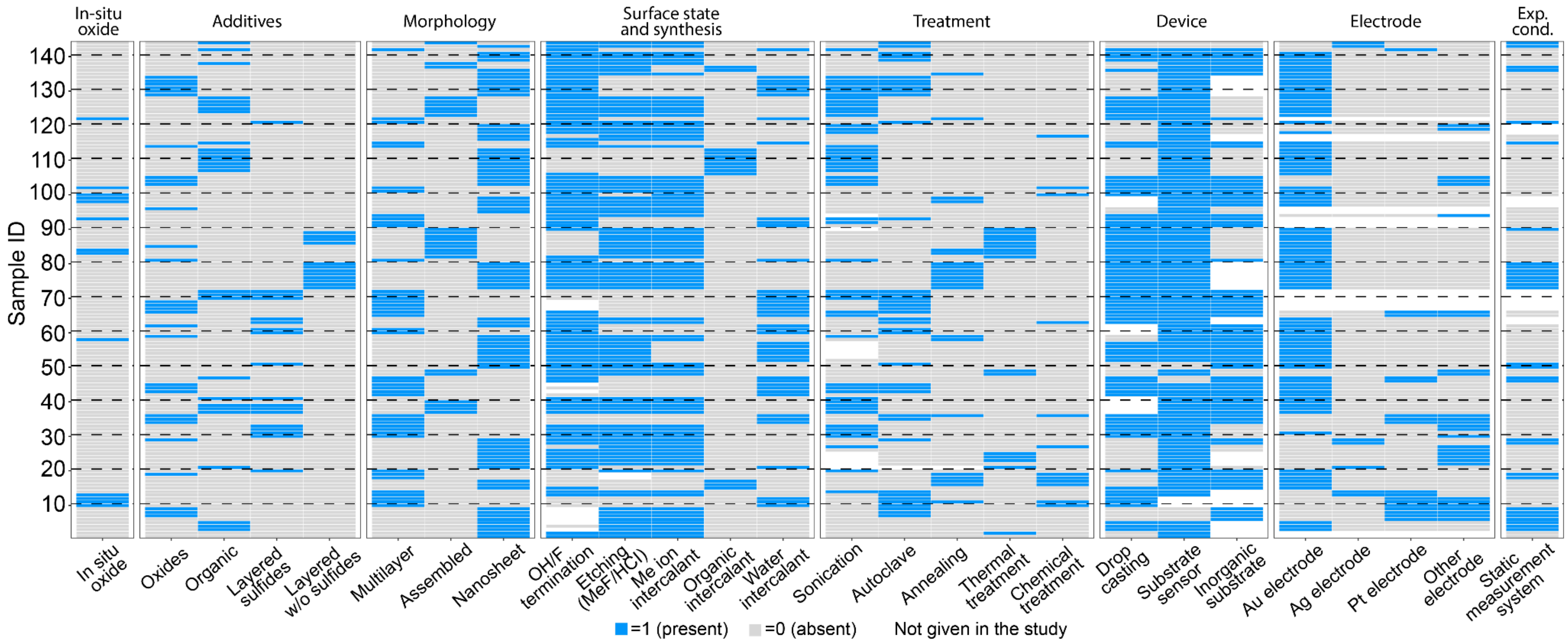

Figure S17. Distribution of categorical moderator variables in the sample: Heatmap showing the presence or absence of the study characteristics and experimental features (moderator variables) that are coded as dummy variables for each sample in the dataset. Samples are listed on the y-axis; moderator variables are grouped by major clusters (following Table S2). Blue indicates the feature is present for a given sample, gray indicates absence, and white indicates that there is no information given in the study.

References cited in Table S3. List of primary studies

1. Cai, Y. *et al.* Single-atom Pd confined in Ti3C2 nanosheet for boosted room-temperature trace NO2 monitoring. *Nano Research* **18**, 94907285 (2025).
2. Cao, L. C. T. *et al.* Ti3C2Tx/MoO3 composite as an ultrasensitive and selective sensing material for a room-temperature nitrogen dioxide sensor. *Applied Surface Science* **676**, 161025 (2024).
3. Choi, J. *et al.* In Situ Formation of Multiple Schottky Barriers in a Ti3C2 MXene Film and its Application in Highly Sensitive Gas Sensors. *Adv Funct Materials* **30**, 2003998 (2020).
4. Das, A., Maji, B., Sahoo, S. J., Singh, J. & Dash, P. Facile Design of Ti3C2TX (MXene) Modified Spherical SrTiO3 Structure toward Highly Sensitive Room Temperature Detection of NO2. *ACS Appl. Electron. Mater.* acsaelm.4c00783 (2024).
5. Devara, I. K. G., Kwon, M. J., Cho, S., Kwon, D. & Park, J. H. Enhanced sensitivity of zero‐bias‐operated MXene chemiresistive sensor via lignin hybridization. *EcoMat* **6**, e12453 (2024).
6. Dhariwal, N. *et al.* Engineering an Ultrafast Ambient NO2 Gas Sensor Using Cotton-Modified LaFeO3/MXene Composites. *ACS Sens.* **9**, 6800–6814 (2024).
7. Ding, Y. *et al.* MOF-Derived NiO–SnO2/Ti3C2Tx Composite Materials for Detection of ppb-Level NO2 at Room Temperature. *ACS Appl. Nano Mater.* **8**, 8927–8936 (2025).
8. Fan, C. *et al.* Fast and recoverable NO2 detection achieved by assembling ZnO on Ti3C2Tx MXene nanosheets under UV illumination at room temperature. *Nanoscale* **14**, 3441–3451 (2022).
9. Fan, J. *et al.* Synthesis and characterization of an ultra-thin BiOCl/MXene heterostructure for the detection of NO2 at room temperature with enhanced moisture resistance. *J. Mater. Chem. A* **10**, 25714–25724 (2022).
10. Feng, G. *et al.* DFT investigation and Polypyrrole sensitization mechanism of a selective NO2 sensor for room temperature application based on MXene@PPy heterojunction. *Surfaces and Interfaces* **40**, 103099 (2023).
11. Gasso, S. & Mahajan, A. Development of Highly Sensitive and Humidity Independent Room Temeprature NO2 Gas Sensor Using Two Dimensional Ti3C2Tx Nanosheets and One Dimensional WO3 Nanorods Nanocomposite. *ACS Sens.* **7**, 2454–2464 (2022).
12. Gasso, S. & Mahajan, A. MXene decorated tungsten trioxide nanocomposite-based sensor capable of detecting NO2 gas down to ppb-level at room temperature. *Materials Science in Semiconductor Processing* **152**, 107048 (2022).
13. Gasso, S. & Mahajan, A. Self-Powered Wearable Gas Sensors Based on l-Ascorbate-Treated MXene Nanosheets and SnO2 Nanofibers. *ACS Appl. Nano Mater.* **6**, 6678–6692 (2023).
14. Gasso, S., Sohal, M. K. & Mahajan, A. MXene modulated SnO2 gas sensor for ultra-responsive room-temperature detection of NO2. *Sensors and Actuators B: Chemical* **357**, 131427 (2022).
15. Guo, F. *et al.* A room-temperature NO2 sensor based on Ti3C2TX MXene modified with sphere-like CuO. *Sensors and Actuators B: Chemical* **375**, 132885 (2023).
16. Hermawan, A. *et al.* Achieving high-performance NO2 sensors via vertically-aligned Ti3C2Tx nanoarchitectures. *Sensors and Actuators B: Chemical* **433**, 137509 (2025).
17. Hilal, M., Yang, W., Hwang, Y. & Xie, W. Tailoring MXene Thickness and Functionalization for Enhanced Room-Temperature Trace NO2 Sensing. *Nano-Micro Lett.* **16**, 84 (2024).
18. Hu, J., Wang, X., Lei, H., Luo, M. & Zhang, Y. Plasmonic photothermal driven MXene-based gas sensor for highly sensitive NO2 detection at room temperature. *Sensors and Actuators B: Chemical* **407**, 135422 (2024).
19. Jian, Y. *et al.* The prior rules of designing Ti3C2Tx MXene-based gas sensors. *Front. Chem. Sci. Eng.* **15**, 505–517 (2021).
20. Kim, S. *et al.* Three-Dimensional MoS2/MXene Heterostructure Aerogel for Chemical Gas Sensors with Superior Sensitivity and Stability. *ACS Nano* **17**, 19387–19397 (2023).
21. Kim, S. *et al.* Surface functionalized MXene ink-enabled washable smart e-textiles with exceptional gas sensing properties. *Materials Today* **88**, 251–262 (2025).
22. Le, V. T. *et al.* Flexible and high-sensitivity sensor based on Ti3C2–MoS2 MXene composite for the detection of toxic gases. *Chemosphere* **291**, 133025 (2022).
23. Li, C. *et al.* Ti3C2Tx Nanosheet/Bi2S3 Nanobelt Composites for NO2 Gas Sensing. *ACS Appl. Nano Mater.* **7**, 13062–13070 (2024).
24. Li, D. *et al.* Epitaxy Growth of Hofmann MOF on Monolayer MXene for ppb‐Level Room‐Temperature NOx Sensing. *Adv Funct Materials* **e11574** (2025).
25. Liu, S. *et al.* Enhanced room-temperature NO2 sensing performance of SnO2/Ti3C2 composite with double heterojunctions by controlling co-exposed {221} and {110} facets of SnO2. *Sensors and Actuators B: Chemical* **365**, 131919 (2022).

26. Liu, S. *et al.* ZnO/Ti3C2 composite with oxygen vacancies and Schottky barrier for effective detection of ppb-level NO2 at room temperature. *Applied Surface Science* **610**, 155440 (2023).
27. Liu, S. *et al.* 2D/2D {001} TiO2-x/Ti3C2Tx composite with ultra-high sensing performance for NO2 at room temperature. *Sensors and Actuators B: Chemical* **404**, 135258 (2024).
28. Liu, S. *et al.* Enhanced NO2 gas-sensing performance of 2D Ti3C2/TiO2 nanocomposites by in-situ formation of Schottky barrier. *Applied Surface Science* **567**, 150747 (2021).
29. Liu, X., Zhang, H., Shen, T. & Sun, J. Flexible resistive NO2 gas sensor of SnO2@Ti3C2Tx MXene for room temperature application. *Ceramics International* **50**, 2459–2466 (2024).
30. Liu, Z. *et al.* Synergistic Etching and Hydrogen Bonding-Induced Self-Assembly of MXene/MOF Hybrid Aerogel for Flexible Room-Temperature Gas Sensing. *ACS Sens.* **9**, 3641–3651 (2024).
31. Liu, Z. *et al.* A 2D/2D/2D Ti3C2Tx@TiO2@MoS2 heterostructure as an ultrafast and high-sensitivity NO2 gas sensor at room-temperature. *J. Mater. Chem. A* **10**, 11980–11989 (2022).
32. Peng, H. *et al.* Gas-sensitive performance of metal-organic framework-derived CuO NPs/Ti3C2Tx MXene heterostructures for efficient NO2 detection at room temperature. *Journal of Alloys and Compounds* **980**, 173657 (2024).
33. Quan, W. *et al.* Fully Flexible MXene-based Gas Sensor on Paper for Highly Sensitive Room-Temperature Nitrogen Dioxide Detection. *ACS Sens.* **8**, 103–113 (2023).
34. Quan, W. *et al.* Quantum Confinement and End-Sealing Effects for Highly Sensitive and Stable Nitrogen Dioxide Detection: Homogeneous Integration of Ti3C2Tx-Based Flexible Gas Sensors. *ACS Sens.* **9**, 4578–4590 (2024).
35. Sardana, S., Debnath, A. K., Aswal, D. K. & Mahajan, A. WS2 nanosheets decorated multi-layered MXene based chemiresistive sensor for efficient detection and discrimination of NH3 and NO2. *Sensors and Actuators B: Chemical* **394**, 134352 (2023).
36. Ta, Q. T. H., Thakur, D. & Noh, J.-S. Enhanced Gas Sensing Performance of ZnO/Ti3C2Tx MXene Nanocomposite. *Micromachines* **13**, 1710 (2022).
37. Ta, Q. T. H., Sreedhar, A., Tri, N. N. & Noh, J.-S. In situ growth of TiO2 on Ti3C2Tx MXene for improved gas-sensing performances. *Ceramics International* **50**, 27227–27236 (2024).
38. Ta, Q. T. H., Tran, N. M., Tri, N. N., Sreedhar, A. & Noh, J.-S. Highly surface-active Si-doped TiO2/Ti3C2Tx heterostructure for gas sensing and photodegradation of toxic matters. *Chemical Engineering Journal* **425**, 131437 (2021).
39. Tang, L. *et al.* Molten salt-modified Ti3C2Tx MXene with tunable oxygen-functionalized surfaces for effective detection of NO2 at room temperature. *Ceramics International* **50**, 21619–21629 (2024).
40. Tang, L. *et al.* Room Temperature Resistive NO2 Sensor Based on SnS2 Quantum Dots/Ti3C2Tx MXene Heterostructures. *ACS Appl. Nano Mater.* **8**, 4636–4648 (2025).
41. Thanh Hoai Ta, Q., Ngoc Tri, N. & Noh, J.-S. Improved NO2 gas sensing performance of 2D MoS2/Ti3C2Tx MXene nanocomposite. *Applied Surface Science* **604**, 154624 (2022).
42. Tran, N. M., Ta, Q. T. H. & Noh, J.-S. rGO/Ti3C2Tx heterostructures for the efficient, room-temperature detection of multiple toxic gases. *Materials Chemistry and Physics* **273**, 125087 (2021).
43. Tseng, S.-F., Lin, Y.-H., Zhou, M.-H., Hsu, S.-H. & Hsiao, W.-T. Synthesis of Ti3C2Tx/ZnO composites decorated with PEDOT:PSS for NO2 gas sensors. *Int J Adv Manuf Technol* **126**, 2269–2281 (2023).
44. Wang, D. *et al.* Multifunctional poly(vinyl alcohol)/Ag nanofibers-based triboelectric nanogenerator for self-powered MXene/tungsten oxide nanohybrid NO2 gas sensor. *Nano Energy* **89**, 106410 (2021).
45. Wang, Y., Fu, J., Hu, H. & Ho, D. d-Band Center Optimization of Ti3C2Tx MXene Nanosheets for Ultrahigh NO2 Gas Sensitivity at Room Temperature. *ACS Appl. Mater. Interfaces* **15**, 40846–40854 (2023).
46. Wang, Y., Fu, J., Xu, J., Hu, H. & Ho, D. Atomic Plasma Grafting: Precise Control of Functional Groups on Ti3C2Tx MXene for Room Temperature Gas Sensors. *ACS Appl. Mater. Interfaces* **15**, 12232–12239 (2023).
47. Wu, X. *et al.* Fabrication of SnO2-TiO2-Ti3C2Tx hybrids with multiple-type heterojunctions for enhanced gas sensing performance at room temperature. *Applied Surface Science* **581**, 152364 (2022).
48. Xia, Y. *et al.* MXene/WS2 hybrids for visible-light-activated NO2 sensing at room temperature. *Chem. Commun.* **57**, 9136–9139 (2021).
49. Xu, Q., Zong, B., Yang, Y., Li, Q. & Mao, S. Black phosphorus quantum dots modified monolayer Ti3C2Tx nanosheet for field-effect transistor gas sensor. *Sensors and Actuators B: Chemical* **373**, 132696 (2022).
50. Yamunasree, B. *et al.* Ultrasensitive and highly selective NO2 gas sensing of porous MXene nanoribbon assemblies. *Carbon* **228**, 119364 (2024).
51. Yan, H. *et al.* 2H-MoS2/Ti3C2Tx MXene composites for enhanced NO2 gas sensing properties at room temperature. *Sensors and Actuators Reports* **4**, 100103 (2022).

52. Yang, Z. *et al.* Self-Assembly 3D Porous Crumpled MXene Spheres as Efficient Gas and Pressure Sensing Material for Transient All-MXene Sensors. *Nano-Micro Lett.* **14**, 56 (2022).
53. Yang, Z. *et al.* Flexible resistive NO2 gas sensor of three-dimensional crumpled MXene Ti3C2Tx/ZnO spheres for room temperature application. *Sensors and Actuators B: Chemical* **326**, 128828 (2021).
54. Yang, Z. *et al.* The Introduction of Defects in Ti3C2Tx and Ti3C2Tx‐Assisted Reduction of Graphene Oxide for Highly Selective Detection of ppb‐Level NO2. *Adv Funct Materials* **32**, 2108959 (2022).
55. Zhang, B. *et al.* High-Performance Ppb Level NO2 Gas Sensor Based on Colloidal SnO2 Quantum Wires/Ti3C2Tx MXene Composite. *Nanomaterials* **12**, 4464 (2022).
56. Zhang, C. *et al.* Laser Processing of Crumpled Porous Graphene/MXene Nanocomposites for a Standalone Gas Sensing System. *Nano Lett.* **23**, 3435–3443 (2023).
57. Zhang, W. *et al.* Au nanoparticles decorated flower-like SnS2/Ti3C2 MXene microcomposite for high performance NO2 sensing at room temperature. *Applied Surface Science* **637**, 157957 (2023).
58. Zhang, Z. *et al.* Enhanced Room-Temperature NO2 Sensing through Deep Functional Group Hybridization in Nitrogen-Doped Monolayer Ti3C2Tx. *ACS Sens.* **9**, 4134–4142 (2024).
59. Zhang, Z. *et al.* Key to unlocking NO2 sensing performance of monolayer Ti3C2Tx: Regulating OH/F functional groups. *Chemical Engineering Journal* **484**, 149620 (2024).
60. Zhao, Q. *et al.* Enhanced Blocking Effect: A New Strategy to Improve the NO2 Sensing Performance of Ti3C2Tx by γ-Poly(l-glutamic acid) Modification. *ACS Sens.* **6**, 2858–2867 (2021).
61. Zhou, L., Hu, Y., Li, S., Liu, X. & Zhang, J. Passivation of MXene via atomic layer deposition of SnO2 to achieve improved NO2 sensing. *Applied Physics Letters* **123**, 161902 (2023).

Additional references cited in the supplement

1. Chen, W. *et al.* In-situ doping nickel single atoms in two-dimensional MXenes analogue support for room temperature NO2 sensing. *Nano Res.* **15**, 9544–9553 (2022).
2. Choi, J. *et al.* N–p-Conductor Transition of Gas Sensing Behaviors in Mo2CTx MXene. *ACS Sens.* **7**, 2225–2234 (2022).
3. Kumar, A. N. & Pal, K. Amine-functionalized stable Nb_2CT_x MXene toward room temperature ultrasensitive NO_2 gas sensor. *Mater. Adv.* **3**, 5151–5162 (2022).
4. Li, X. *et al.* MXene-activated graphene oxide enhancing NO2 capture and detection of surface acoustic wave sensors. *Sensors and Actuators B: Chemical* **401**, 135006 (2024).
5. Rathi, K., Arkoti, N. K. & Pal, K. Fabrication of Delaminated 2D Metal Carbide MXenes (Nb_2CT_x) by CTAB‐based NO_2 Gas Sensor with Enhanced Stability. *Adv Materials Inter* **9**, 2200415 (2022).
6. Simonenko, E. P. *et al.* Gas-Sensitive Properties of ZnO/Ti2CTx Nanocomposites. *Micromachines* **14**, 725 (2023).
7. Simonenko, E. P. *et al.* Preparation and Chemosensory Properties of Nanocomposite Obtained by Hydrothermal Modification of Ti2CTx with Hierarchically Organized Co(CO3)0.5(OH)·0.11H2O. *Russ. J. Inorg. Chem.* **69**, 1291–1300 (2024).
8. Simonenko, E. P. *et al.* Chemosensory Properties of Nanocomposite Ti0.2V1.8CTx–V2O5–SnO2. *Russ. J. Inorg. Chem.* **69**, 1454–1462 (2024).
9. Simonenko, E. P. *et al.* Preparation and Chemosensory Properties of Composite Material Ti2CTx–10 mol % SnO2. *Russ. J. Inorg. Chem.* **69**, 1587–1595 (2024).
10. Wang, B. *et al.* Room-temperature ppb-level NO2 sensor based on three-dimensional Mo2CTx nano-crumpled spheres. *Sensors and Actuators B: Chemical* **399**, 134790 (2024).
11. Zhang, Y. *et al.* Highly sensitive and selective NO2 sensor of alkalized V2CT MXene driven by interlayer swelling. *Sensors and Actuators B: Chemical* **344**, 130150 (2021).
12. Zhang, Y. *et al.* Synergistic effect of charge transfer and interlayer swelling in V2CTx/SnS2 driving ultrafast and highly sensitive NO2 detection at room temperature. *Sensors and Actuators B: Chemical* **411**, 135788 (2024).
13. Zhao, Q. *et al.* Edge‐Enriched $Mo_2TiC_2T_x/MoS_2$ Heterostructure with Coupling Interface for Selective NO_2 Monitoring. *Adv Funct Materials* **32**, 2203528 (2022).
